# A new and efficient procedure for the synthesis of hexahydropyrimidine-fused 1,4-naphthoquinones

**DOI:** 10.3762/bjoc.11.137

**Published:** 2015-07-22

**Authors:** Marcelo Isidoro P Reis, Vinícius R Campos, Jackson A L C Resende, Fernando C Silva, Vitor F Ferreira

**Affiliations:** 1 Universidade Federal Fluminense, Departamento de Química Orgânica, Campus do Valonguinho, 24020-150, Niterói, RJ, Brazil; 2 Universidade Federal Fluminense, Departamento de Química Inorgânica, Campus do Valonguinho, 24020-150, Niterói, RJ, Brazil

**Keywords:** hydrobenzo[*g*]quinazoline, microwave, naphthoquinone, 1,3-quinazoline, 1,3,5-triazinane

## Abstract

A new and efficient method for the synthesis of hexahydropyrimidine-fused 1,4-naphthoquinones in one step with high yields from the reaction of lawsone with 1,3,5-triazinanes was developed.

## Introduction

**Figure 1:**
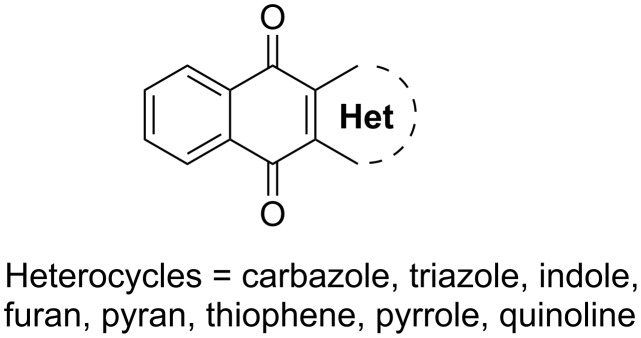
General template for heterocycle-fused 1,4-naphthoquinones.

Quinones represent a diverse family of naturally occurring secondary metabolites [1–3]. Interest in these substances has intensified in recent years due to their pharmacological importance [4]. Heterocycle-fused naphthoquinones (Figure 1) such as naphtho[2,3-*b*]furan [5–14], naphtho-pyran [15–18], benzo[*f*]indole [19–24], benzo[*g*]quinolone [25], benzo[*b*]carbazole [26], naphtho[2,3-*b*]thiophene [27–33] and naphtho[2,3-*b*]]oxazole [34] have been demonstrated to display various biological activities. There are several specific and general methods reported in the literature that describe the preparation and biological activity of heterocycle-fused naphthoquinone compounds [35]. Recently, Iida et al. described a general method for the construction of pyrrole-, furan- and thiophene-fused naphthoquinones [36].

**Scheme 1:**
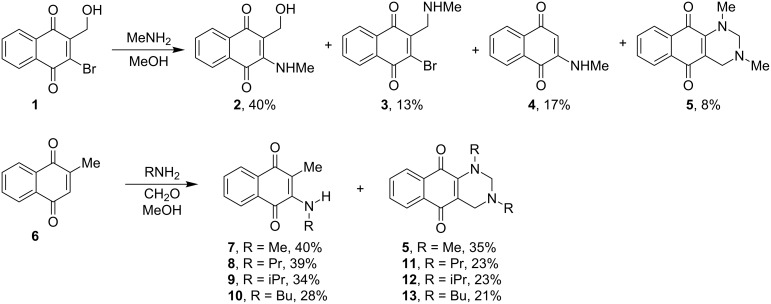
Methods for the preparation of hexahydropyrimidine-fused 1,4-naphthoquinones.

For several years, our group has been interested in developing new synthetic methods for the preparation of heterocycle-fused 1,4-naphthoquinones or heterocycle-tethered 1,4-naphthoquinones. 1,3-Quinazolines are nitrogenated heterocycles that are present in several products. However, there are only three procedures for the preparation of hexahydropyrimidine-fused 1,4-naphthoquinones available. Some of them are restrictive, troublesome and produce the hexahydropyrimidine-1,4-naphthoquinones in low yields. Möhrle and Herbruggen synthesized unsymmetrical hexahydropyrimidine-fused 1,2- and 1,4-naphthoquinones by the reaction of 4-amino-l,2-naphthoquinone and 2-amino-1,4-naphthoquinone with amines and formaldehyde in moderate yields [37]. Ohta et al. studied the nucleophilic addition reaction of methylamine to 2-bromo-3-hydroxymethyl-1,4-naphthoquinone (**1**) and obtained hexahydropyrimidine-fused 1,4-naphthoquinone (**5**) as side product (Scheme 1) [38–39]. This reaction was non-selective and formed a mixture of four products (**2–5**) that are derived from the addition of methylamine to both of the electrophilic sites of the starting material **1**. In the same publication, the authors reported another method that was more selective, which involved the addition of two equivalents of a monoalkyl amine (R-NH_2_) to menadione (**6**) to give hexahydropyrimidine-fused 1,4-naphthoquinones **5**, **11**–**13** in low yields.

## Results and Discussion

**Scheme 2:**
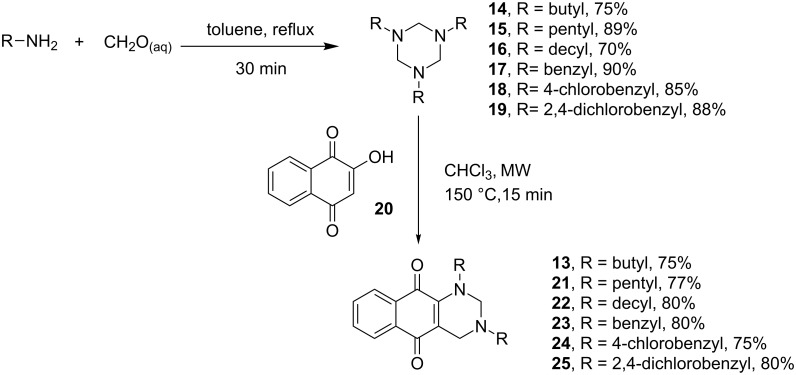
Synthesis of hexahydropyrimidine-fused 1,4-naphthoquinones **13** and **21–25**.

Herein we describe a new method for the synthesis of hexahydropyrimidine-fused 1,4-naphthoquinones (**13** and **21–25**) in high yields, from the sequential reaction of readily available 1,3,5-triazinanes **14–19** with 2-hydroxy-1,4-naphthoquinone (**20**, or lawsone) under microwave irradiation (Scheme 2).

The 1,3,5-triazinanes have several synthetic and biological applications [40]. These substances are easily prepared from commercially available amines and formaldehyde in toluene in yields ranging from 75–90%. Barluenga and coworkers [41] have previously shown that 1,3,5-triazinanes undergo fragmentation at elevated temperatures to form 3 equivalents of alkyl- or aryl-formimines in situ. The latter compounds may serve as electrophilic agents for aminoalkylation reactions. Our research group also investigated the aminoalkylation of 2-amino-1,4-naphthoquinone with formaldehyde under microwave irradiation to produce two series of *N*,*O*-acetals and *N*,*S*-acetals. These compounds were obtained in good yields, and several of them showed promising antibacterial activity [42].

**Figure 2:**
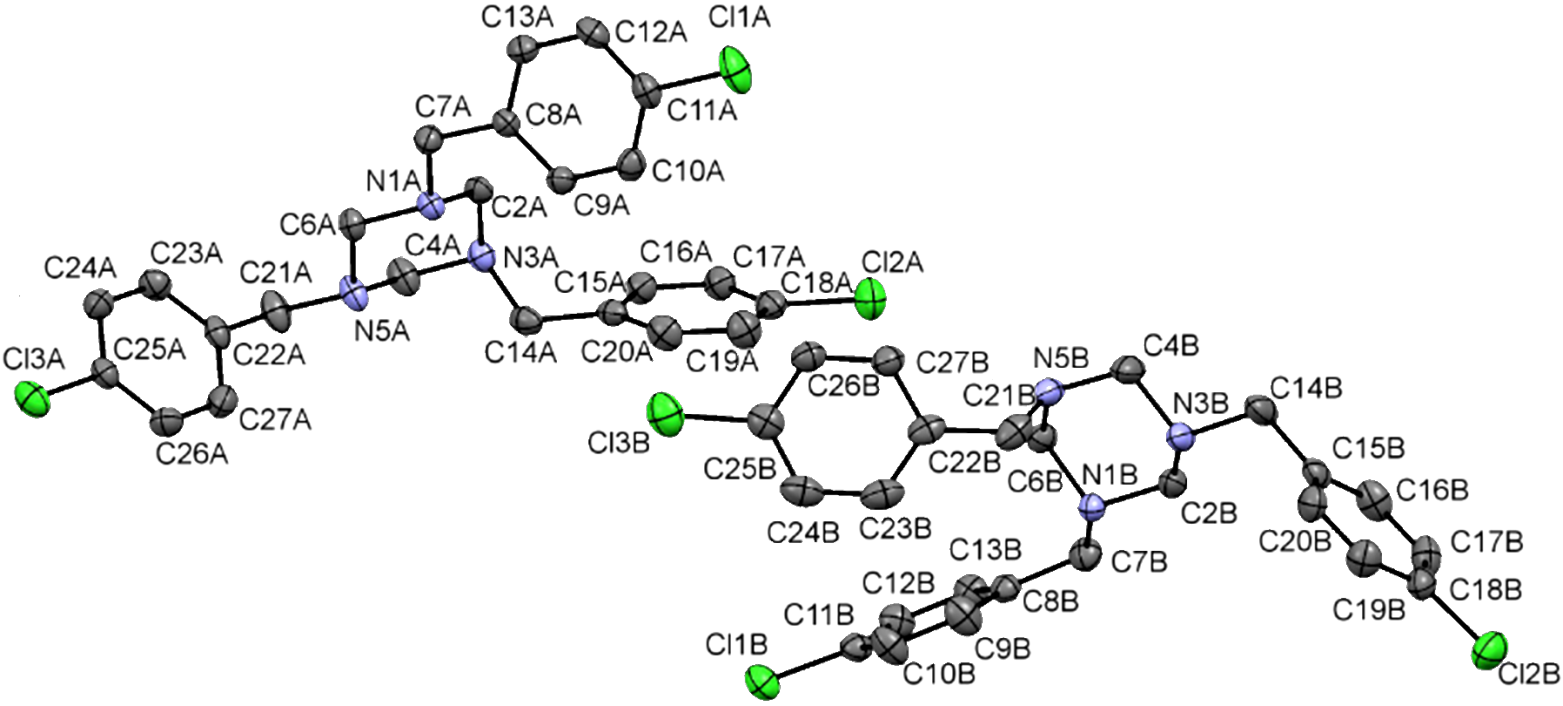
ORTEP diagram of compound **18** depicted with ellipsoids drawn at the 50% probability level and the atom-numbering scheme.

The structures of the synthesized 1,3,5-triazinanes were confirmed by spectroscopic techniques such as NMR, ^1^H and ^13^C-APT, infrared spectroscopy (FTIR) and high resolution mass spectrometry. The synthesis of compounds **14** [43] and **17** [44] has been previously reported in the literature. The structure of compound **18** was confirmed by X-ray diffraction analysis and Figure 2 shows the ORTEP diagram of this compound. The details of the crystal data and refinements are collected in Supporting Information File 1, Table S1.

The crystal structure of compound **18** contains two molecules per asymmetric unit. Two factors explain the differences between molecules A and B (Figure 2): (1) different intermolecular interactions and (2) small differences in the torsion angles of the *p*-substituted benzyl groups. The 1,3,5-triazinane rings of **18** (molecules A and B) adopt a chair conformation, with Cremer–Pople puckering parameters *q*_2_ and Φ_2_ of 0.023(9) Å and 323.0(20)º, respectively, for molecule A, and *q*_2_ and Φ_2_ of 0.016(3) Å and 2.0(25)º, respectively, for molecule B.

**Figure 3:**
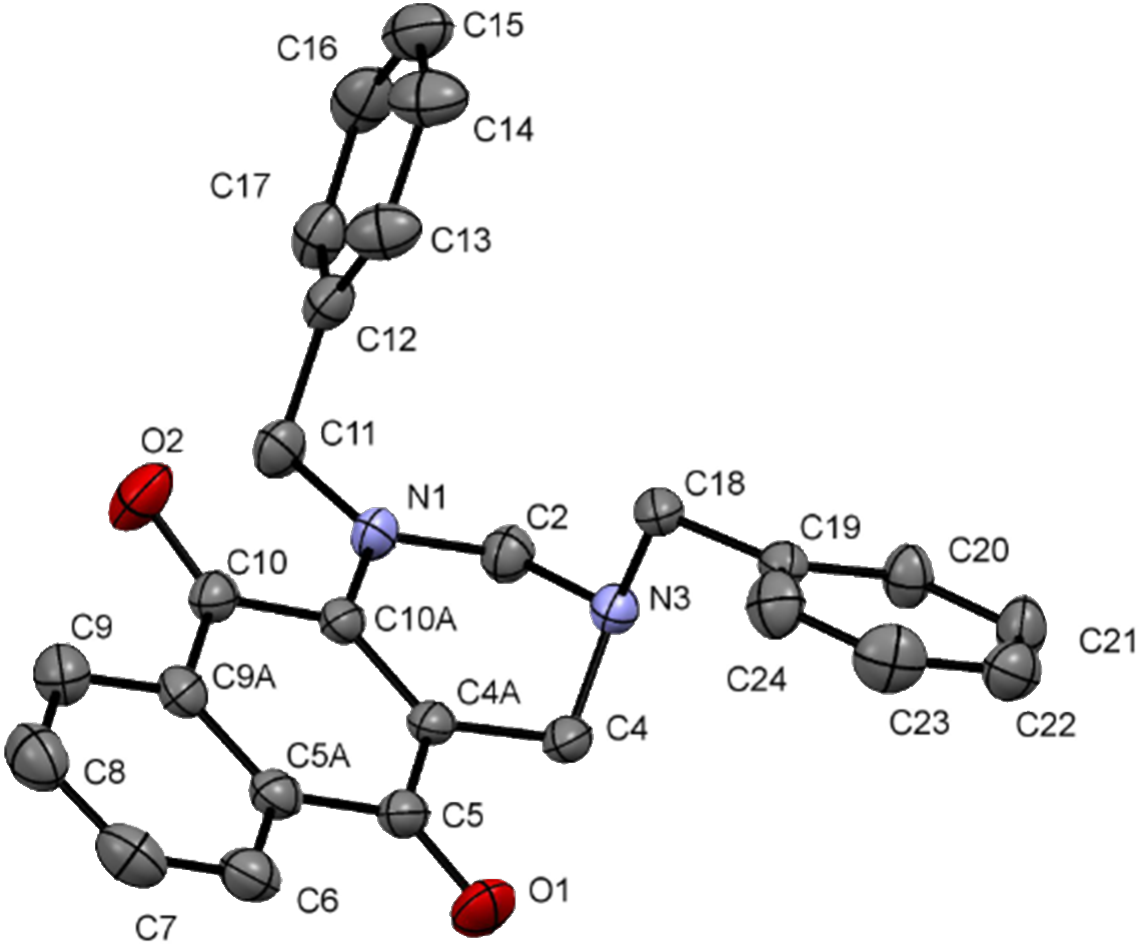
ORTEP diagram of compound **23** depicted with ellipsoids drawn at the 50% probability level and the atom-numbering scheme.

Next the reaction between 1,3,5-triazinanes **14–19** and 2-hydroxy-1,4-naphthoquinone (**20**) was developed to prepare fused hexahydropyrimidine-1,4-naphthoquinones **13** and **21–25** using in situ generated alkyl formimines. Performing the reaction without heating proceeds very slow and after 24 hours extensive degradation products were observed. By elevating the temperature and or changing the solvent some product is formed but the yields were very low. On the other hand, when the reactions were conducted in an equimolar ratio under microwave irradiation (300 Monowave model brand Aanton Paar) in chloroform for 15 minutes at a temperature of 150 °C, the desired products were obtained in good yields (75–80%, Scheme 2). All structures of the benzo-fused tetrahydroquinazolines were characterized by ^1^H NMR and ^13^C-APT, infrared spectroscopy (FTIR) and high resolution mass spectrometry. Using compound **23** as an example, it can be observed that its ^1^H NMR spectrum contains a doublet of doublets (*J* = 0.98 and 7.8 Hz) at 7.98 ppm attributed to the hydrogen H-5 of the naphthoquinone moiety, a multiplet at 7.16–7.25 ppm related to the aromatic protons on the phenyl ring (H-3”’–H, 7”’), and four singulets corresponding to the H1’ (C–C*H*_2_–N), H1” (N–C*H*_2_–N), H-1”’ (N–C*H*_2_–Ph) and H-1”” (N–C*H*_2_–Ph) methylene protons at 3.60 ppm, 3.77 ppm, 3.84 ppm and 4.75 ppm, respectively. The high resolution mass spectrum of compound **23** showed a molecular ion [M + H]^+^ at *m*/*z* 395.2062, which corresponds with the calculated mass for C_26_H_22_N_2_O_2_H [M + H]^+^ of 395.1715. The structure of compound **23** was further elucidated by X-ray diffraction analysis (Figure 3). It confirmed the insertion of two imines to form a hexahydropyrimidine ring which is coupled to a 1,4-naphthoquinone moiety. Figure 3 shows the ORTEP diagram of compound **23** and the details of the crystal data and refinements are given in Supporting Information File 1, Table S2.

In the crystal structure, the six-membered nitrogen-containing ring of **23** adopts a half boat conformation (Cremer–Pople puckering parameters [45] *q*_2_ and Φ_2_ of 0.374(9) Å and 119.0(1)) in which the N(3) atom is located at 0.641(2) Å out of the plane of the other five atoms C(2), N(1), C(4a), C(10) and C(4).

**Scheme 3:**
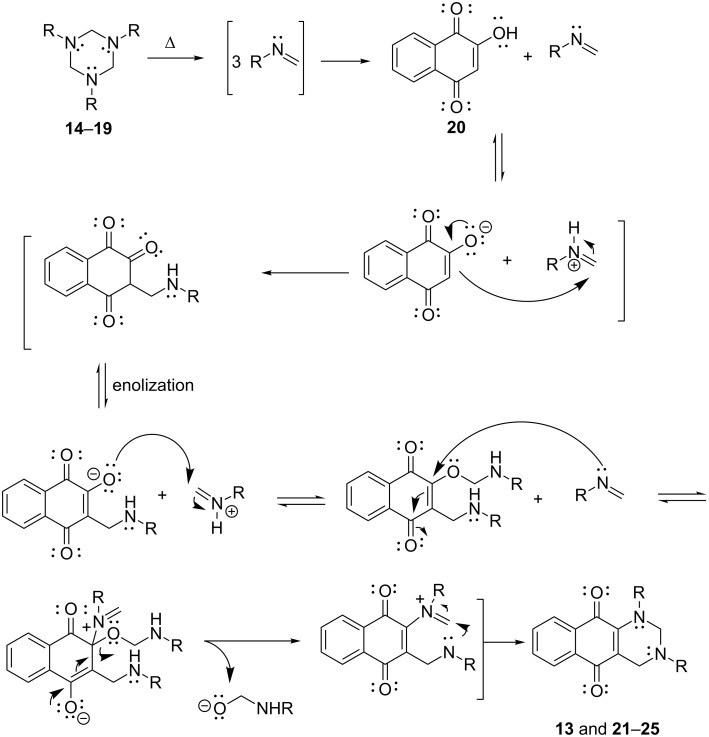
Proposed mechanism for the formation of **13** and **21–25**.

A possible mechanism that could explain the formation of the tetrahydrobenzo[*g*]quinazolines **13** and **21–25** is shown in Scheme 3. It initially involves the in situ formation of three thermally generated iminium ions or the equivalent methylformimines from the corresponding triazinane which then react with lawsone (**20**) at its two nucleophilic sites, thus forming products (Scheme 3).

## Conclusion

A new method for the synthesis of new heterocyclic hexahydropyrimidines fused to a 1,4-naphthoquinone system (**13** and **21–25**) was developed. The products were obtained in one step, under microwave irradiation and with excellent yields. This method is a more efficient alternative for the preparation of benzo-fused tetrahydroquinazolindiones than the method described in the literature. The structures of all starting 1,3,5-triazinanes and products were confirmed by spectroscopical methods and X-ray diffraction analysis.

## Supporting Information

File 1 Experimental procedures and spectral data.

## References

[1] Patai S (1974). The chemistry of the quinoid compounds.

[2] Patai S, Rappoport Z (1988). The Chemistry of the Functional Groups. The Chemistry of the Quinonoid Compounds.

[3] Thomson R H (1997). Naturally Occurring Quinones IV.

[4] Ferreira V F, da Rocha D R, da Silva F C, Ferreira P G, Boechat N A, Magalhães J L (2013). Expert Opin Ther Pat.

[5] Rao M M, Kingston D G I (1982). J Nat Prod.

[6] Cardoso M F C, Rodrigues P C, Oliveira M E I M, Gama I L, da Silva I M C B, Santos I O, Rocha D R, Pinho R T, Ferreira V F, de Souza M C B V (2014). Eur J Med Chem.

[7] Eyong K O, Kumar P S, Kuete V, Folefoc G N, Nkengfack E A, Baskaran S (2008). Bioorg Med Chem Lett.

[8] Ferreira M d P S B C, Cardoso M F d C, da Silva F d C, Ferreira V F, Lima E S, Souza J V B (2014). Ann Clin Microbiol Antimicrob.

[9] Teimouri M B, Khavasi H R (2007). Tetrahedron.

[10] da Silva A J M, Netto C D, Pacienza-Lima W, Torres-Santos E C, Rossi-Bergmann B, Maurel S, Valentin A, Costa P R R (2009). J Braz Chem Soc.

[11] Ueda S, Umemura T, Dohguchi K, Matsuzaki T, Tokuda H, Nishino H, Iwashima A (1994). Phytochemistry.

[12] Wu C, Johnson R K, Mattern M R, Wong J C, Kingston D G I (1999). J Nat Prod.

[13] Jiménez-Alonso S, Guasch J, Estévez-Braun A, Ratera I, Veciana J, Ravelo A G (2011). J Org Chem.

[14] Reichstein A, Vortherms S, Bannwitz S, Tentrop J, Prinz H, Müller K (2012). J Med Chem.

[15] Ferreira S B, da Silva F d C, Pinto A C, Gonzaga D T G, Ferreira V F (2009). J Heterocycl Chem.

[16] da Costa E C B, Amorim R, da Silva F C, Rocha D R, Papa M P, de Arruda L B, Mohana-Borges R, Ferreira V F, Tanuri A, da Costa L J (2013). PLoS One.

[17] da Rocha D R, de Souza A C G, Resende J A L C, Santos W C, dos Santos E A, Pessoa C, de Moraes M O, Costa-Lotufo L V, Montenegro R C, Ferreira V F (2011). Org Biomol Chem.

[18] Ferreira V F, Ferreira S B, da Silva F d C (2010). Org Biomol Chem.

[19] Tseng C-M, Wu Y-L, Chuang C-P (2004). Tetrahedron.

[20] Shvartsberg M S, Kolodina E A, Lebedeva N I, Fedenok L G (2009). Tetrahedron Lett.

[21] Inman M, Moody C J (2010). J Org Chem.

[22] Acosta A, de la Cruz P, De Miguel P, Diez-Barra E, de la Hoz A, Langa F, Loupy A, Majdoub M, Martin N, Sanchez C (1995). Tetrahedron Lett.

[23] Oostveen E A, Speckamp W N (1987). Tetrahedron.

[24] Comer E, Murphy W S (2003). ARKIVOC.

[25] Bala B D, Muthusaravanan S, Perumal S (2013). Tetrahedron Lett.

[26] Inoue A, Nomura Y, Kuroki N, Konishi K (1958). J Synth Org Chem, Jpn.

[27] Kobayashi K, Yoneda K, Uchida M, Matsuoka H, Morikawa O, Konishi H (2001). Heterocycles.

[28] Valderrama J A, Astudillo C, Tapia R A, Prina E, Estrabaud E, Mahieux R, Fournet A (2002). Chem Pharm Bull.

[29] Tapia R A, Alegria L, Pessoa C D, Salas C, Cortés M J, Valderrama J A, Sarciron M-E, Pautet F, Walchshofer N, Fillion H (2003). Bioorg Med Chem.

[30] Ryu C-K, Choi I H, Lee J Y, Jung S H (2005). Heterocycles.

[31] Valderrama J A, Espinoza O, Rodriguez J, Theoduloz C (2009). Lett Org Chem.

[32] Campiglia P, Aquino C, Bertamino A, De Simone N, Sala M, Castellano S, Santoriello M, Grieco P, Novellino E, Gomez-Monterrey I M (2010). Org Biomol Chem.

[33] Gomez-Monterrey I, Campiglia P, Aquino C, Bertamino A, Granata I, Carotenuto A, Brancaccio D, Stiuso P, Scognamiglio I, Rusciano M R (2011). J Med Chem.

[34] Hamdan A J, Al-Jaroudi S (2003). Arabian J Sci Eng, Sect A.

[35] Sartori M F (1963). Chem Rev.

[36] Ueda K, Yamashita M, Sakaguchi K, Tokuda H, Iida A (2013). Chem Pharm Bull.

[37] Möhrle H, Schulte Herbrüggen G (1991). Arch Pharm.

[38] Ohta S, Hinata Y, Yamashita M, Kawasaki I, Jinda Y, Horie S (1994). Chem Pharm Bull.

[39] Ohta S, Hinata Y, Yamashita M, Kawasaki I, Shoji T, Yoshikawa H, Obana Y (1994). Chem Pharm Bull.

[40] Reis M I P, Romeiro G A, Damasceno R, da Silva F d C, Ferreira V F (2013). Rev Virtual Quim.

[41] Barluenga M, Bayón A M, Campos P, Asensio G, Gonzalez-Nuñez E, Molina Y (1988). J Chem Soc, Perkin Trans 1.

[42] Jordão A K, Novais J, Leal B, Escobar A C, dos Santos Júnior H M, Castro H C, Ferreira V F (2013). Eur J Med Chem.

[43] Guillemin J C, Denis J M (1985). J Chem Soc, Chem Commun.

[44] Smith G S, Berlin K D, Zisman S A, Holt E M, Green V A, Van Der Helm D (1988). Phosphorus Sulfur Relat Elem.

[45] Cremer D, Pople J A (1975). J Am Chem Soc.

