# Supplementary material for: A new and efficient procedure for the synthesis of hexahydropyrimidine-fused 1,4-naphthoquinones
Source: Beilstein J Org Chem. 2015 Jul 22;11:1235–40. doi: 10.3762/bjoc.11.137 (PMC4578459; doi:10.3762/bjoc.11.137)
Supplement: File 1 — Experimental procedures and spectral data. [file Beilstein_J_Org_Chem-11-1235-s001.pdf]

Supporting Information

for

A new and efficient procedure for the synthesis of hexahydropyrimidine-fused 1,4-naphthoquinones

Marcelo Isidoro P. Reis[1], Vinícius R. Campos[1], Jackson A. L. C. Resende[2], Fernando C. Silva[1] and Vitor F. Ferreira[1,*]

Address: [1]Universidade Federal Fluminense, Departamento de Química Orgânica, Campus do Valonguinho, 24020-150, Niterói, RJ, Brazil and [2]Universidade Federal Fluminense, Departamento de Química Inorgânica, Campus do Valonguinho, 24020-150, Niterói, RJ, Brazil

Email: Vitor F. Ferreira - cegvito@vm.uff.br

*Corresponding author

Experimental procedures and spectral data

Table of contents

General procedure for the preparation of **14–19** S2

General procedure for the preparation of **13** and **21–25** S4

References S8

Spectral data S9

General procedure for the preparation of 1,3,5-triazinanes 14–19

To a round-bottomed flask (125 mL) equipped with a reflux condenser was added the appropriate amine (0.05 mmol), toluene (40 mL) and formaldehyde (37%, 4.1 mL). The solution was brought to reflux using an external oil bath and kept stirring for 30 min. Then, the toluene was evaporated under reduced pressure, and the residue was dissolved in ethyl acetate and washed with a saturated aqueous solution of sodium chloride. After evaporation of the solvent under reduced pressure in a rotary evaporator, the residue was purified by silica gel column chromatography using hexane/ethyl acetate 9:1 as the eluent [1].

1,3,5-Tributyl-1,3,5-triazinane (**14**) was isolated as oil in 75% yield. IR ν_{max} (cm^{-1}, film): 2955, 2929, 2862, 2791, 1684, 1466, 1374, 1304, 1261, 1240, 1192, 1141, 1109, 1012, 969, 952, 912, 869, 802, 734, 665. ^1H NMR (500 MHz, $CDCl_3$) δ (ppm): 0.84 (9H, t, *J* 7.2 Hz), 1.20-1.43 (12H, m), 2.28-2.36 (6H, m), 3.23 (6H, s). ^{13}C NMR (APT, 125 MHz, $CDCl_3$) δ (ppm): 13.8, 20.5, 29.6, 52.4, 74.6.

1,3,5-Tripentyl-1,3,5-triazinane (**15**) was isolated as oil in 89% yield. IR ν_{max} (cm^{-1}, film): 2955, 2928, 2859, 2790, 1463, 1375, 1226, 1182, 1141, 1110, 1016, 982, 920, 900, 728. ^1H NMR (500 MHz, $CDCl_3$) δ (ppm): 0.88 (9H, t, *J* 7.3 Hz), 1,26-1,34 (12H, m), 1.46 (6H, pent, *J* 7.3 Hz), 2.38-2.41 (6H, m), 3.29 (6H, s). ^{13}C NMR (APT, 125 MHz, $CDCl_3$) δ (ppm): 14.1, 20.8, 29.9, 52.7, 74.0.

1,3,5-Tridecyl-1,3,5-triazinane (**16**) was isolated as oil in 70% yield. IR ν_{max} (cm^{-1}, film): 2954, 2921, 2852, 2800, 1466, 1366, 1301, 1263, 1209, 1119, 1011, 979, 918, 721. ^1H NMR (500 MHz, DMSO-D_6) δ (ppm): 0.88 (9H, t, *J* 6.7 Hz), 1.26-1.47 (48H, m), 2.35-2.42 (6H, m), 3.29 (6H, s). ^{13}C NMR (APT, 125 MHz, DMSO-D_6) δ (ppm): 14.0, 22.6, 27.4, 27.5, 29.2, 29.4, 29.5, 31.8, 52.7, 74.6.

1,3,5-Tribenzyl-1,3,5-triazinane (**17**) was isolated as white solid in 90% yield. m.p. 44-46 °C, (lit. 43-46 °C).**Fehler! Textmarke nicht definiert.** IR ν_{max} (cm^{-1}, KBr): 3022, 2851, 2805, 2774, 1602, 1494, 1452, 1397, 1356, 1314, 1260, 1169, 1151, 1119, 1065, 1029, 1014, 920, 907, 878, 853, 736, 704, 697. ^1H NMR (500 MHz,

$CDCl_3$) δ (ppm): 3.42 (6H, s), 3.67 (6H, s), 7.18-7.32 (15H, m). ^{13}C NMR (APT, 125 MHz, $CDCl_3$) δ (ppm): 56.9, 73.6, 126.8, 128.1, 128.7, 138.2.

1,3,5-Tris(4-chlorobenzyl)-1,3,5-triazinane (**18**) was isolated as white solid oil in 85% yield. m.p. 94-95 ºC. IR ν_{max} (cm^{-1}, KBr): 2813, 1596, 1487, 1448, 1403, 1349, 1249, 1158, 1085, 1014, 976, 915, 882, 837, 801, 714, 655. ^1H NMR (500 MHz, $CDCl_3$) δ (ppm): 3.37 (6H, s), 3.59 (6H, s), 7.19-7.25 (12H, m). ^{13}C NMR (APT, 125 MHz, $CDCl_3$) δ (ppm): 56.4, 73.6, 128.6, 130.3, 133.1, 136.9.

Table S1. Crystallographic data of compound **18**:

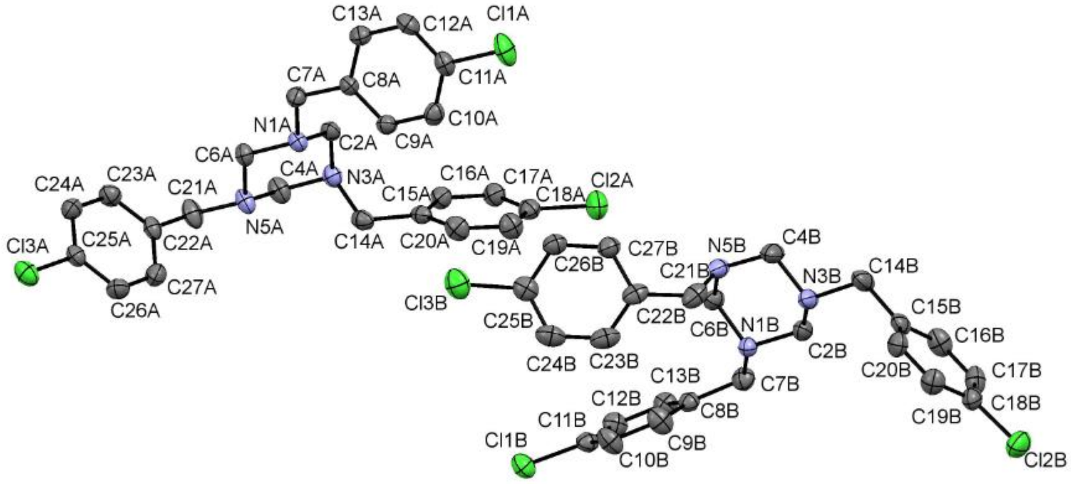

Empirical formula	$C_{24}H_{24}Cl_3N_3$
Formula weight	460.81
Temperature/K	293(2)
Crystal system	Triclinic
Space group	P-1
a/Å	9.7908(15)
b/Å	15.489(2)
c/Å	15.519(2)
α/°	83.337(5)
β/°	78.500(5)
γ/°	83.738(5)
Volume/Å^3	2281.8(6)
Z	4
ρ_{calc}g/cm^3	1.341
μ/mm^{-1}	0.418
F(000)	960.0

Crystal size/mm^3	0.60 × 0.43 × 0.12
Radiation	MoKα (λ = 0.71073)
2Θ range for data collection/°	3.96 to 51.596
Index ranges	-11 ≤ h ≤ 11, -18 ≤ k ≤ 18, -18 ≤ l ≤ 18
Reflections collected	40337
Independent reflections	8719 [R_{int} = 0.0626, R_{sigma} = 0.0435]
Data/restraints/parameters	8719/0/541
Goodness-of-fit on F^2	0.996
Final R indexes [I>=2σ (I)]	R_1 = 0.0483, wR_2 = 0.1337
Final R indexes [all data]	R_1 = 0.0925, wR_2 = 0.1628
Largest diff. peak/hole / e Å^{-3}	0.37/-0.35

1,3,5-Tris(2,4-dichlorobenzyl)-1,3,5-triazinane (**19**) was isolated as white solid in 88% yield. m.p. 102-103 ºC. IR ν_{max} (cm^{-1}, KBr): 1586, 1469, 1383, 1336, 1251, 1202, 1163, 1097, 1014, 981, 924, 855, 819, 765, 733, 657. ^{1}H NMR (500 MHz, DMSO-D_6) δ (ppm): 3.51 (6H, s), 3.76 (6H, s), 7.17 (3H, dd, *J* 1.8 and 8.2 Hz), 7.33-7.63 (6H, m). ^{13}C NMR (APT, 125 MHz, DMSO-D_6) δ (ppm): 53.3, 73.6, 127.1, 129.5, 131.3, 133.6,134.8, 135.1.

General procedure for the preparation of 1,3-quinazoline derivatives 13 and 21–25

An equimolar mixture of lawsone (**20**, 2.87 mmol) and the appropriate 1,3,5-triazinane (2.87 mmol) dissolved in chloroform (6 mL) was placed in a 10 mL microwave vial. The reaction was irradiated for 15 minutes at 150 °C in a microwave apparatus for synthesis. Afterwards TLC control indicated complete consumption of the starting materials and the formation of quinazolines **13** and **21–25**. The products were subsequently purified by column chromatography using hexane/ethyl acetate 98:2 as the eluent.

1,3-Dibutyl-1,2,3,4-tetrahydrobenzo[*g*]quinazoline-5,10-dione (**13**) was isolated as oil in 75% yield. IR ν_{max} (cm^{-1}, film): 2956, 2930, 2870, 1670, 1615, 1592, 1548, 1458, 1367, 1270, 1231, 1157, 1106, 932, 789, 721, 685; ^{1}H NMR (500 MHz, $CDCl_3$) δ (ppm): 0.86 (3H, t, *J* 7.0 Hz), 0.91 (3H, t, *J* 7.0 Hz), 1.24-1.38 (4H, m), 1.44-1.51 (2H, m), 1.54-1.65 (2H, m), 2.44-2.49 (2H, m), 3.53-3.58 (2H, m), 3.71 (2H, s), 3.96 (2H, s), 7.50 (1H, dt, *J* 1.5 and 7.6 Hz), 7.58 (1H, dt, 1H, *J* 1.5, 7.6 Hz), 7.87-7.90

(1H, m), 7.93-7.96 (1H, m). ^{13}C NMR (APT, 125 MHz, $CDCl_3$) δ (ppm): 13.7, 13.8, 20.0, 20.3, 29.7, 31.6, 48.2, 51.6, 53.0, 71.0, 113.4, 125.0, 125.9, 131.6, 131.9, 132.5, 133.6, 146.4, 180.6, 182.0. HRESIMS m/z: $(M+H)^+$ 327.2217 (Calculated for $C_{20}H_{27}N_2O_2$: 327.2073).

1,3-Pentyl-1,2,3,4-tetrahydrobenzo[*g*]quinazoline-5,10-dione (**21**) was isolated as an oil in 77% yield. IR ν_{max} (cm^{-1}, film): 2926, 2856, 1671, 1618, 1593, 1552, 1379, 1266, 1106, 722; ^{1}H NMR (500 MHz, $CDCl_3$) δ (ppm): 0.83 (3H, t, *J* 6.8 Hz), 0.86 (3H, t, *J* 6.8 Hz), 1.24-1.34 (8H, m), 1.59-1.65 (6H, m), 2.45 (2H, m), 3.55 (2H, m), 3.71 (2H, s), 3.96 (2H, s), 7.50 (1H, dt, *J* 1.5 and 7.8Hz), 7.58 (1H, dt, 1H, *J* 1.5, 7.6 Hz), 7.87-7.89 (1H, m), 7.94-7.95 (1H, m). ^{13}C NMR (APT, 125 MHz, $CDCl_3$) δ (ppm): 13.9, 22.4, 22.4, 27.3, 28.9, 29.2, 29.4, 29.6, 48.3, 51.9, 53.3, 71.0, 113.5, 125.0, 126.0, 131.6, 131.9, 132.6, 133.6, 146.4, 180.7, 182.0. HRESIMS m/z: $(M+H)^+$ 355.2592 (Calculated for $C_{22}H_{30}N_2O_2$: 355.2386).

1,3-Decyl-1,2,3,4-tetrahydrobenzo[*g*]quinazoline-5,10-dione (**22**) was isolated as an oil in 80% yield. IR ν_{max} (cm^{-1}, film): 2921, 2851,1670, 1619, 1592, 1551, 1465, 1370, 1335, 1267, 1109, 1024, 964, 843, 787, 685; ^{1}H NMR (500 MHz, $CDCl_3$) δ (ppm): 0.85-0.90 (6H, m), 1.34-1.26 (28H, m), 1.51-1.72 (4H, m), 2.49-2.54 (2H, m), 3.58-3.64 (2H, m), 3.78 (2H, s), 4.03 (2H, s), 7.56 (1H, ddd, *J* 1.4, 7.4and 8.9 Hz), 7.64 (1H, ddd, 1H, *J* 1.4, 7.4 and 8.9 Hz), 7.95 (ddd, 1H, *J* 0.5, 1.5 and 7.4 Hz), 8.01 (1H, ddd, *J* 0.5, 1.5 and 7.4 Hz). ^{13}C NMR (APT, 125.0 MHz, $CDCl_3$) δ (ppm): 14.1, 22.6, 26.9, 27.3, 27.8, 29.3, 29.4, 29.5, 29.5, 29.6, 29.6, 31.8, 48.4, 52.0, 53.4, 71.0, 113.5, 125.1, 126.0, 131.7, 132.0, 132.7, 133.7, 146.5, 180.7, 182.1. HRESIMS m/z: $(M+H)^+$ 495.4422 (Calculated for $C_{32}H_{50}N_2O_2$: 495,3951).

1,3-Dibenzyl-1,2,3,4-tetrahydrobenzo[*g*]quinazoline-5,10-dione (**23**) was isolated as a red solid in 80% yield. m.p. 117-118 °C. IR ν_{max} (cm^{-1}, KBr): 2949, 2930, 2837, 1657, 1623, 1588, 1556, 1453, 1381, 1357, 1293, 1272, 1250, 1232, 1209, 1108, 1080, 1065, 940, 751, 726, 720, 701. ^{1}H NMR (500 MHz, $CDCl_3$) δ (ppm): 3.60 (2H, s), 3.77 (2H, s), 3.84 (2H, s), 4.75 (2H, s), 7.16-7.27 (10H, m), 7.53 (1H, dt, *J* 1.5 and 7.8Hz), 7.60 (1H, dt, *J* 1.5 and 7.8 Hz), 7.91 (1H, dd, *J* 1.5 and 7.8 Hz), 7.97 (dd, 1H, *J* 1.5 and 7.8 Hz). ^{13}C NMR (APT, 125 MHz, $CDCl_3$) δ (ppm): 48.7, 54.6, 57.6, 68.9,

116.1, 125.2, 126.2, 127.3, 127.3, 127.5, 128.3, 128.5, 128.8, 132.0, 132.4, 133.7, 137.3, 137.6, 146.8, 181.2, 181.9. HRESIMS m/z: $(M+H)^+$ 395.2062 (Calculated for $C_{26}H_{22}N_2O_2$: 395.1760).

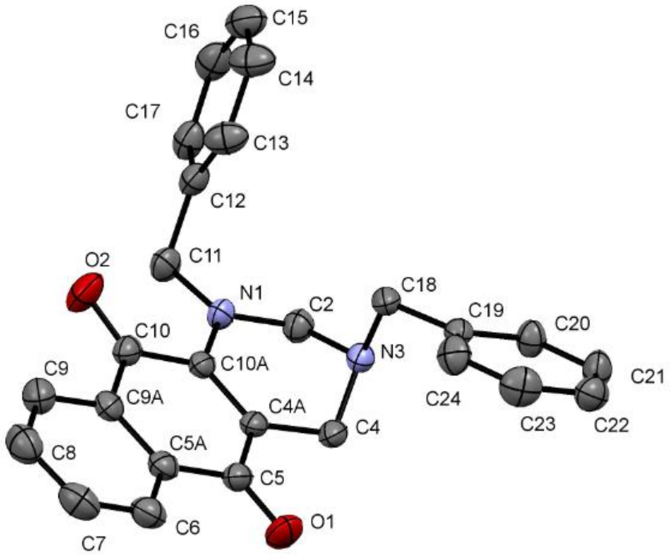

Table S2. X-Ray crystallographic data for compound **23**.

Empirical formula	$C_{26}H_{22}N_2O_2$
Formula weight	394.45
Temperature/K	293(2)
Crystal system	Monoclinic
Space group	$P2_1/c$
a/Å	16.0640(9)
b/Å	5.5113(3)
c/Å	22.5128(12)
α/°	90
β/°	93.298(2)
γ/°	90
Volume/Å^3	1989.84(19)
Z	4
ρ_{calc}g/cm^3	1.317
μ/mm^{-1}	0.084
F(000)	832.0
Crystal size/mm^3	0.45 × 0.27 × 0.11
Radiation	MoKα (λ = 0.71073)
2Θ range for data collection/°	4.304 to 50.7
Index ranges	$-19 \leq h \leq 19$, $-6 \leq k \leq 6$, $-27 \leq l \leq 27$
Reflectionscollected	55863

Independentreflections	3647 [R_{int} = 0.0624, R_{sigma} = 0.0209]
Data/restraints/parameters	3647/0/271
Goodness-of-fit on F^2	1.100
Final R indexes [I>=2σ (I)]	R_1 = 0.0429, wR_2 = 0.1065
Final R indexes [all data]	R_1 = 0.0624, wR_2 = 0.1200
Largest diff. peak/hole / e $Å^{-3}$	0.20/-0.15

1,3-Di-(4-chlorobenzyl)-1,2,3,4-tetrahydrobenzo[*g*]quinazoline-5,10-dione (**24**) wasisolated as oil in 75 % yield (995 mg). IR ν_{max} (cm^{-1}, filme): 2921, 2847, 1667, 1618, 1592, 1551, 1487, 1381, 1349, 1289, 1268, 1250, 1088, 1011, 950, 921, 838, 799, 721, 686. ^{1}HNMR (500 MHz, $CDCl_3$) δ (ppm): 3.61 (2H, s), 3.81 (2H, s), 3.84 (2H, s), 4.73 (2H, s), 7.38 (2H, d, *J* 8.6 Hz), 7.22(2H, d, *J* 8.6 Hz), 7.26 (2H, d, *J* 8.6 Hz), 7.29 (2H, d, *J* 8.6 Hz), 7.62 (1H, dt, *J* 1.4 and7.5 Hz), 7.68 (1H, dt, *J* 1.4 and 7.5 Hz), 7.98 (dd, 1H, *J* 1.4 and 7.5 Hz), 8.04 (1H, dd, *J* 1.4 and 7.5 Hz). ^{13}C NMR (APT, 125 MHz, $CDCl_3$) δ (ppm): 48.8, 54.1, 56.9, 68.6, 116.8, 125.4, 126.3, 128.6,128.7, 129.1, 130.1, 132.0, 132.3, 133.2, 133.4, 133.9, 135.8, 136.1, 146.7, 181.4, 182.0. HRESIMS m/z: $(M+H)^+$ 463.1484 (Calculated for $C_{26}H_{20}Cl_2N_2O_2$: 463.0980).

1,3-Di-(2,4-dichlorobenzyl)-1,2,3,4-tetrahydrobenzo[*g*]quinazoline-5,10-dione (**25**) was isolated as an oil in 80% yield. IR ν_{max} (cm^{-1}, film): 2948, 2821, 1677, 1587, 1471, 1384, 1337, 1257, 1202, 1163, 1138, 1098, 1045, 1014, 982, 946, 856, 824, 763, 732, 696, 659. ^{1}H NMR (500 MHz, $CDCl_3$) δ (ppm): 3.77 (2H, s), 3.83 (2H, s), 3.97 (2H, s), 4.89 (2H, s), 7.20-7.24 (2H, m), 7.33-7.39 (4H, m), 7.61 (1H, dt, 1H, *J* 1.1 and 7.6 Hz), 7.69(1H, dt, 1H, *J* 1.1 and 7.6 Hz), 7.95 (dd, 1H, *J* 1.1 and 7.6 Hz), 8.04 (1H, dd, 1H, *J* 1.1 and 7.6 Hz). ^{13}C NMR (APT, 125.0 MHz, $CDCl_3$) δ (ppm): 48.3, 52.0, 54.1, 69.9, 116.5, 125.4, 126.3, 127.0, 127.3, 129.2, 129.2, 129.3, 129.6, 130.9, 131.7, 132.2, 132.2, 133.3, 133.5, 133.6, 133.7, 133.8, 133.9, 134.6, 146.6, 181.3, 181.7. HRESIMS m/z: $(M+CH_3OH_2)^+$ 561.9746 (Calculated for $C_{27}H_{22}Cl_4N_2O_3$: 562.0385).

References

1. Barluenga, M.; Bayon, A. M.; Campos, P.; Asensio, G.; Gonzalez-Nuiiez, E.; Molina, Y. *J. Chem. Soc. Perkin Trans. 1* **1988**, 1631-1636. doi:10.1039/P19880001631

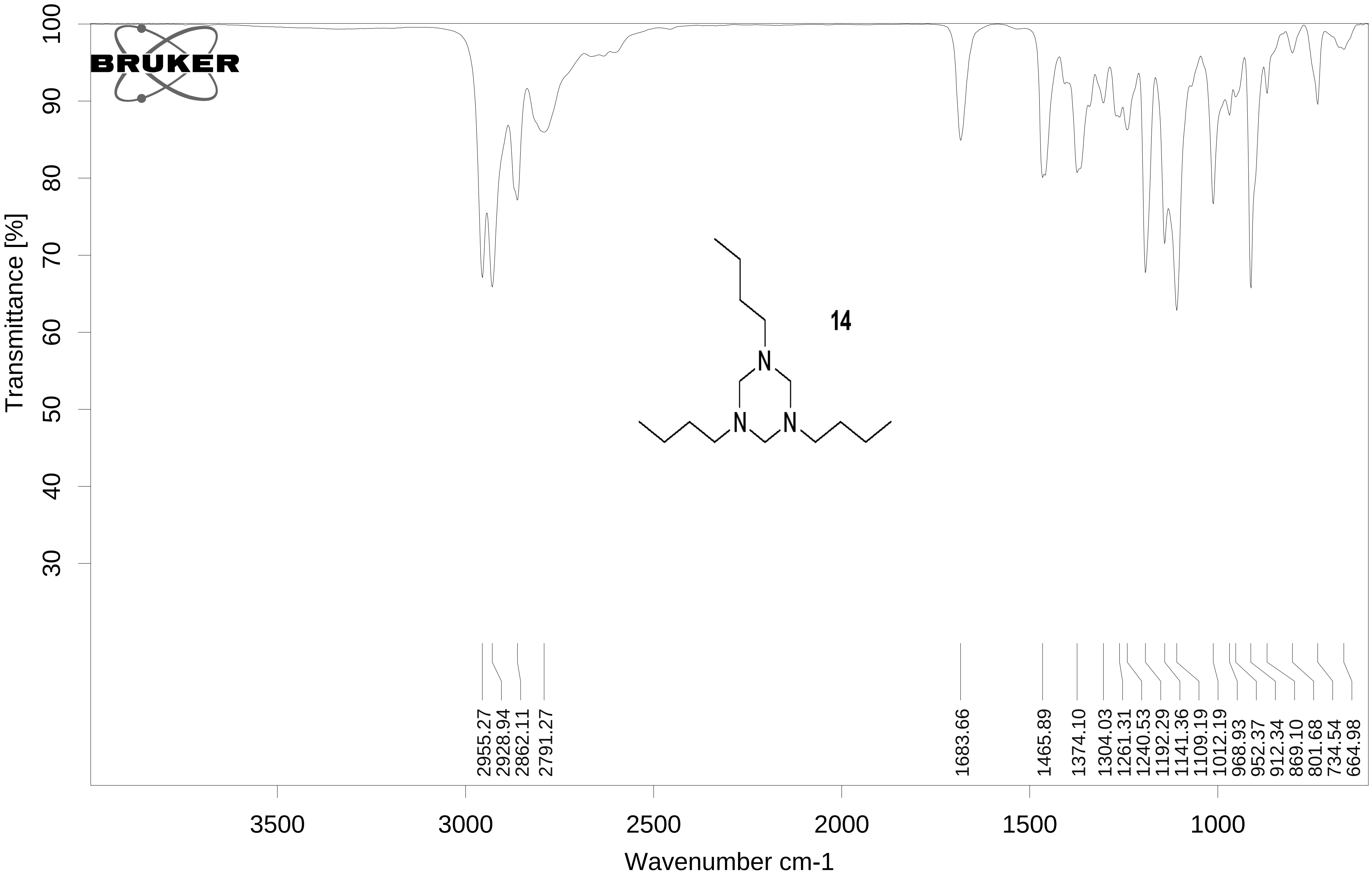

C:\OPUS_7.2.139.1294\MEAS\TBUT.0	TBUT	Instrument type and / or accessory		11/08/2014

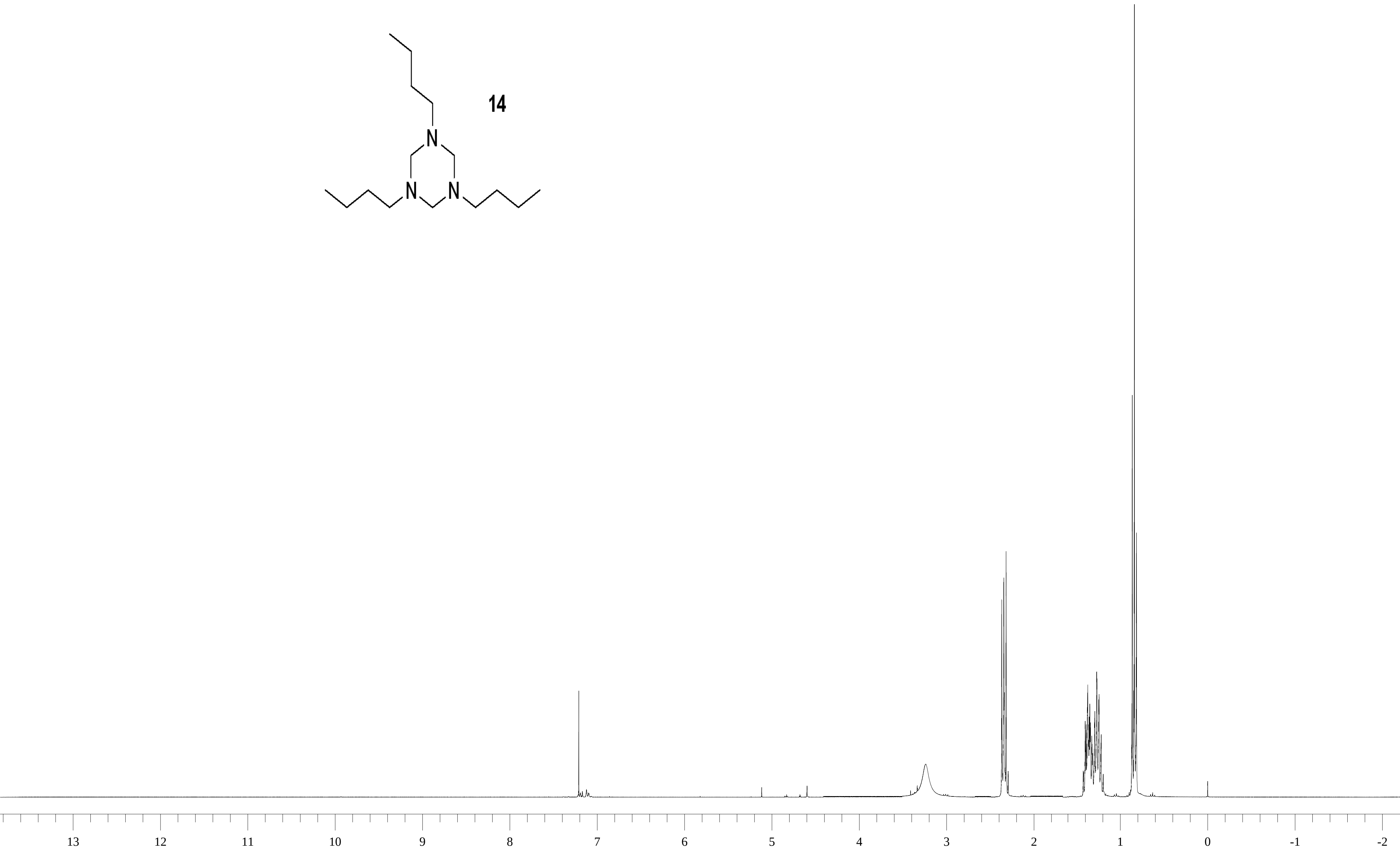
14
13
12
11
10
9
8
7
6
5
4
3
2
1
0
-1
-2

S11

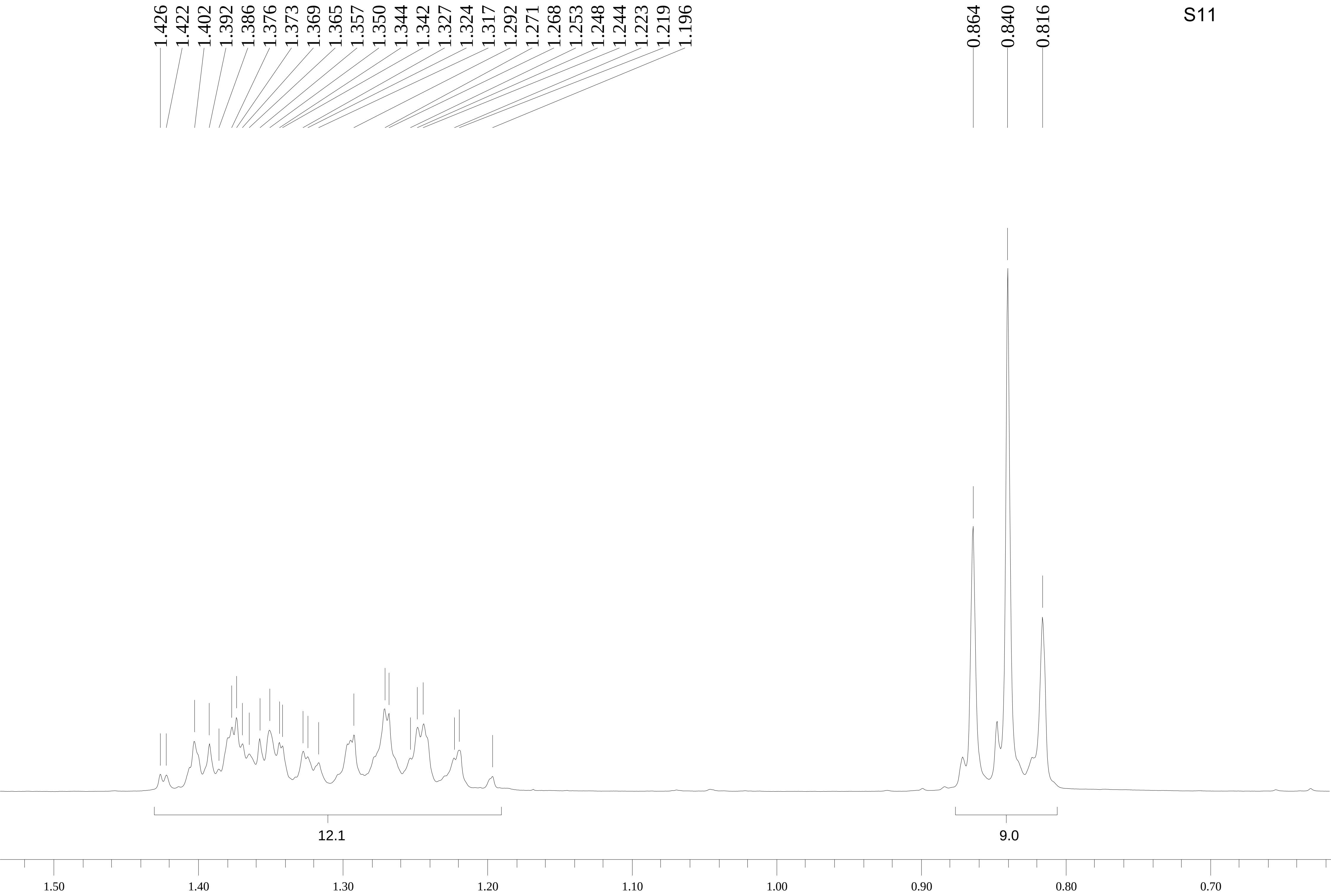

1.426
1.422
1.402
1.392
1.386
1.376
1.373
1.369
1.365
1.357
1.350
1.344
1.342
1.327
1.324
1.317
1.292
1.271
1.268
1.253
1.248
1.244
1.223
1.219
1.196
0.864
0.840
0.816
12.1
9.0
1.50
1.40
1.30
1.20
1.10
1.00
0.90
0.80
0.70

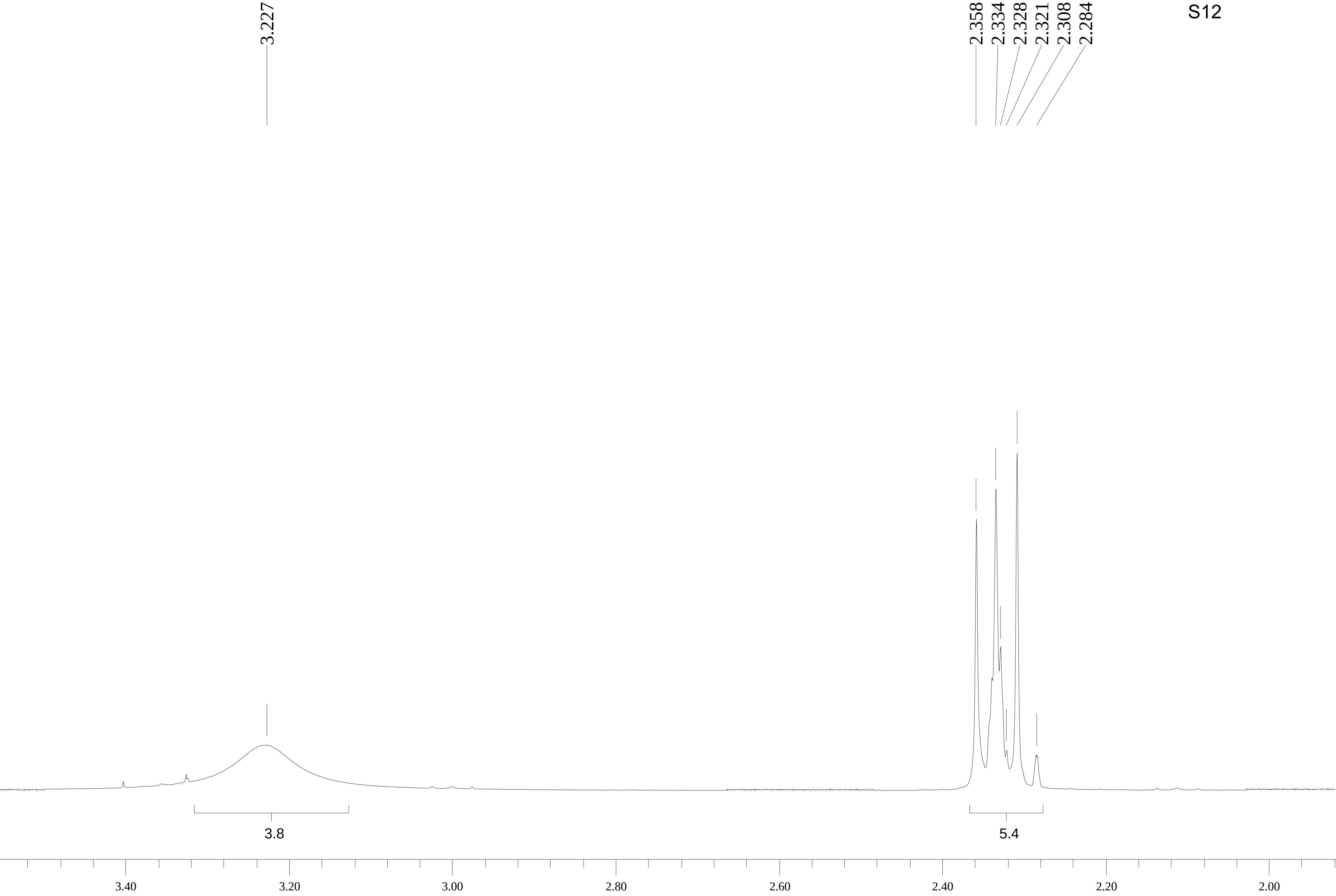

3.227
2.358
2.334
2.328
2.321
2.308
2.284
S12
3.8
5.4
3.40
3.20
3.00
2.80
2.60
2.40
2.20
2.00

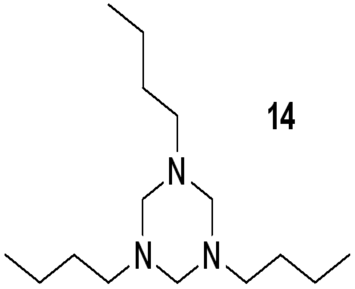

14

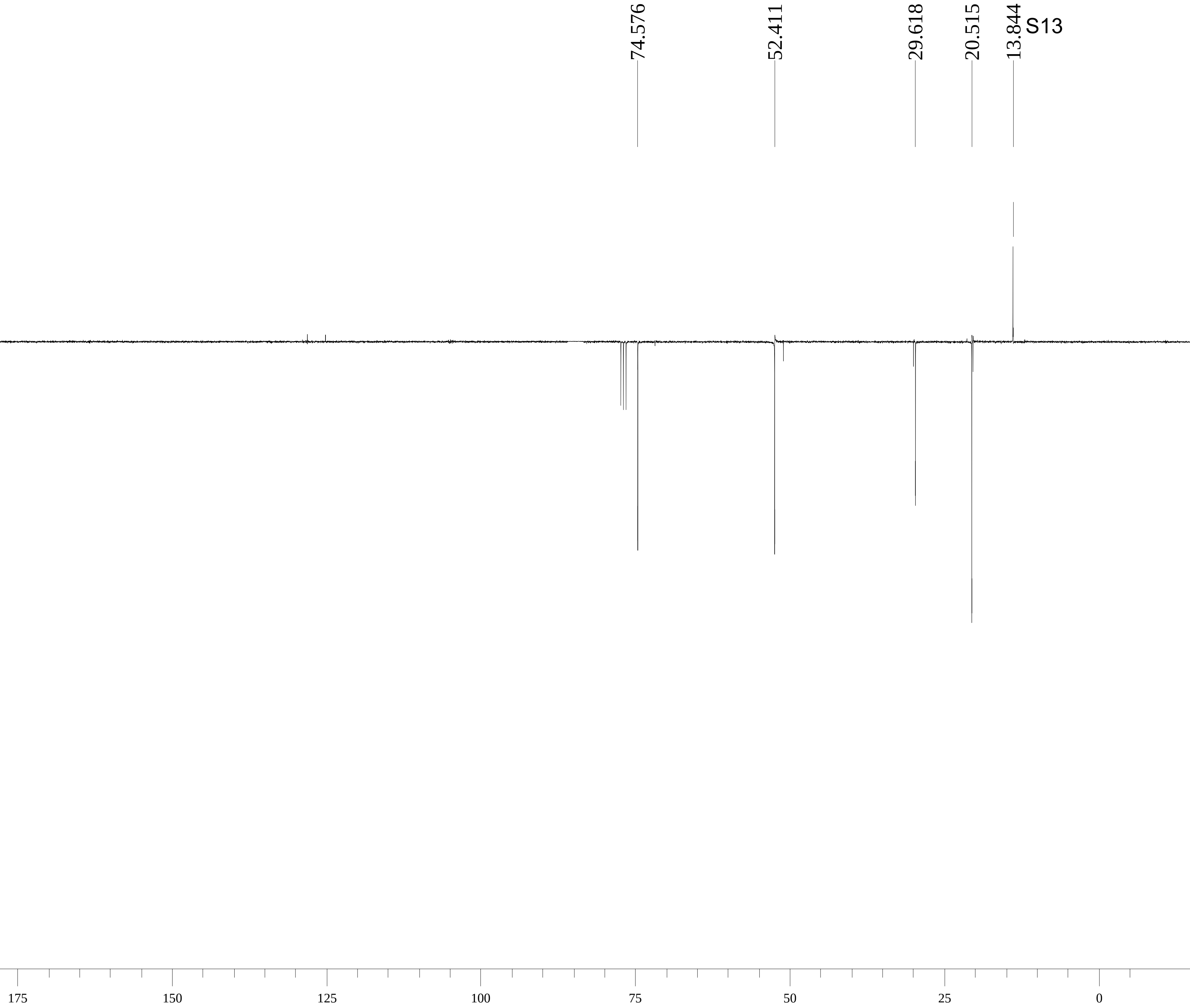

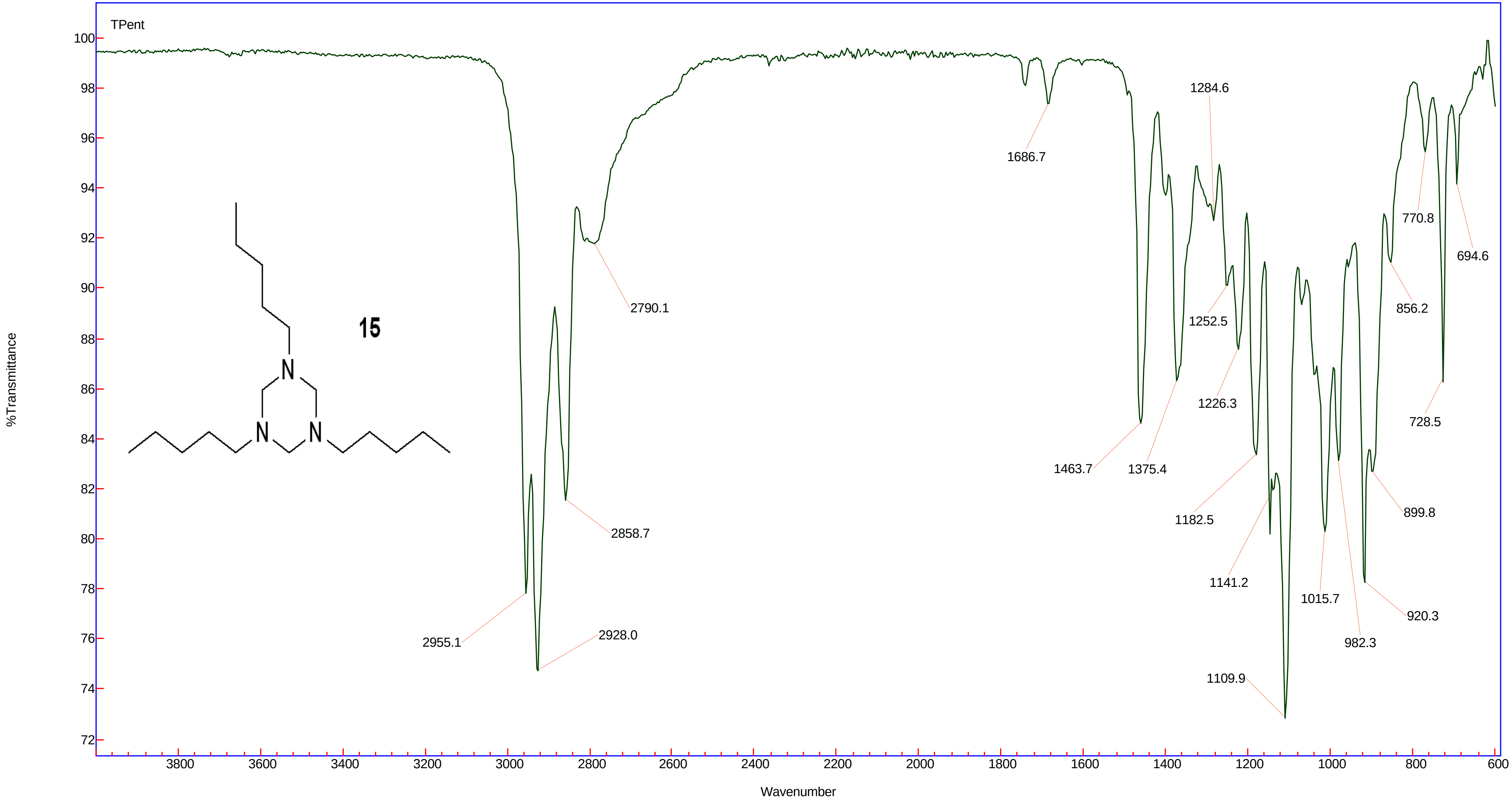
TPent
15
%Transmittance
100
98
96
94
92
90
88
86
84
82
80
78
76
74
72
Wavenumber
3800
3600
3400
3200
3000
2800
2600
2400
2200
2000
1800
1600
1400
1200
1000
800
600
2955.1
2928.0
2858.7
2790.1
1686.7
1463.7
1375.4
1284.6
1252.5
1226.3
1182.5
1141.2
1109.9
1015.7
982.3
920.3
899.8
856.2
770.8
728.5
694.6

Name
TPent

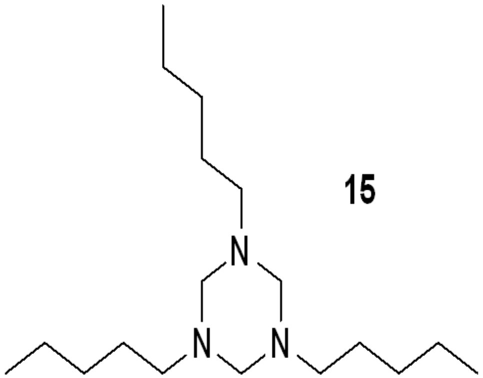

15

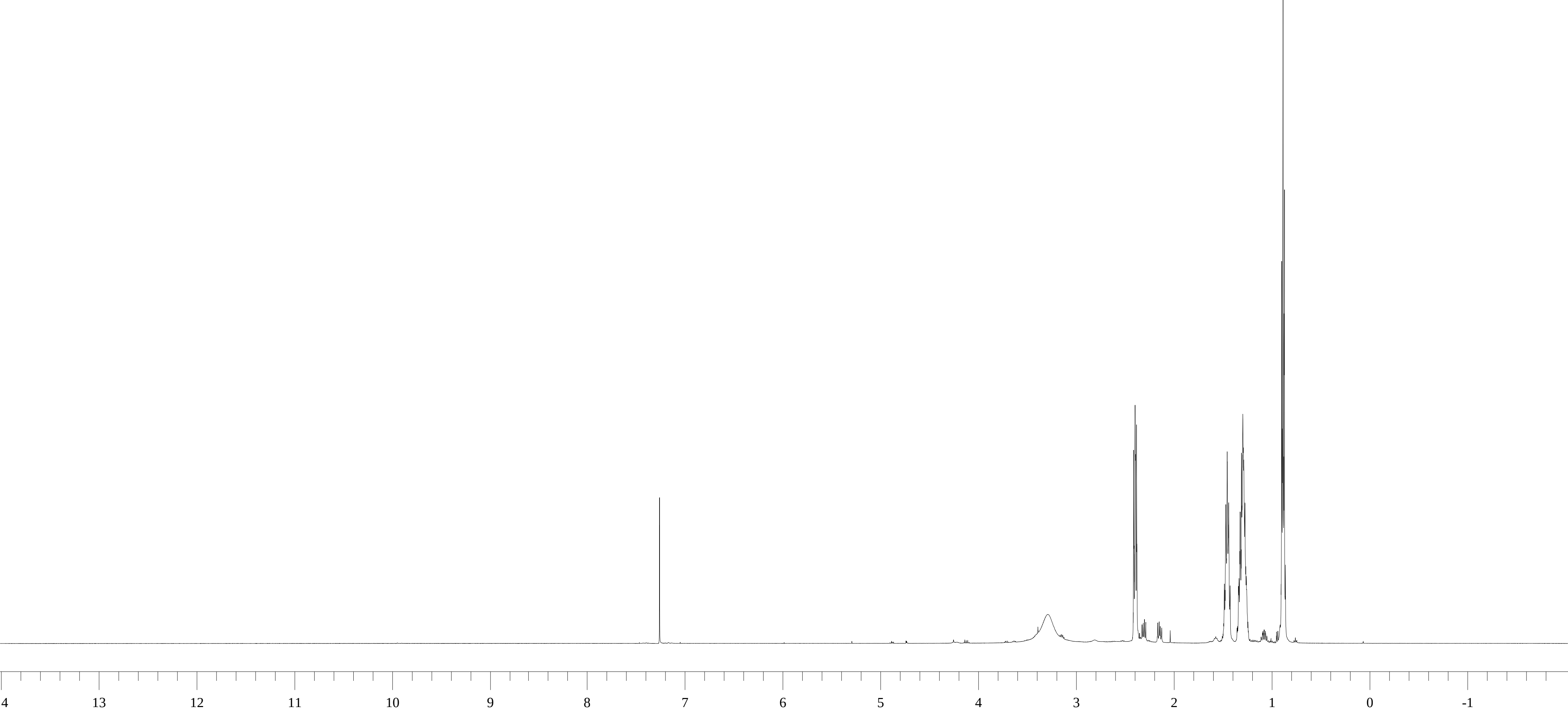

S16

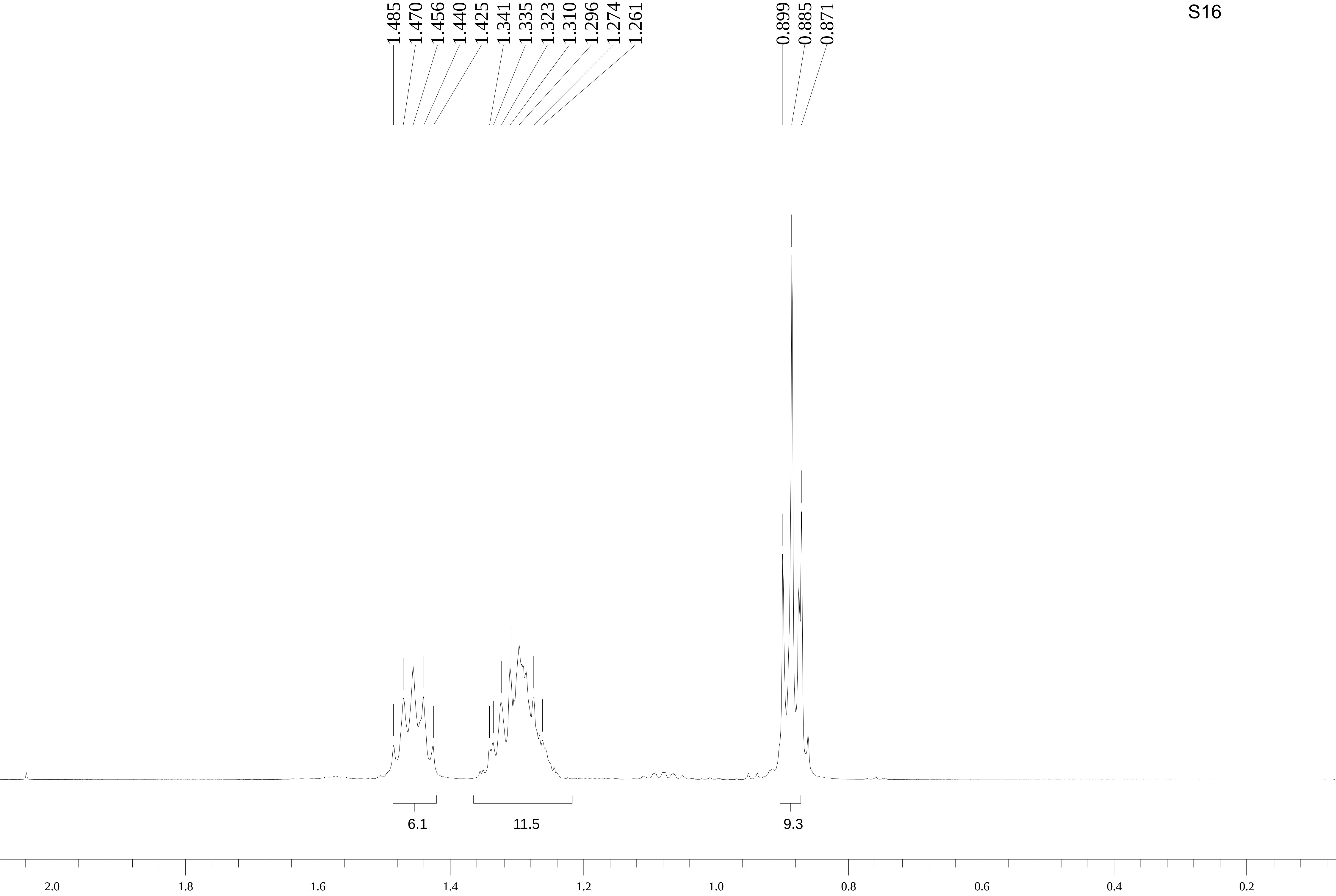
1.485
1.470
1.456
1.440
1.425
1.341
1.335
1.323
1.310
1.296
1.274
1.261
0.899
0.885
0.871
6.1
11.5
9.3
2.0
1.8
1.6
1.4
1.2
1.0
0.8
0.6
0.4
0.2

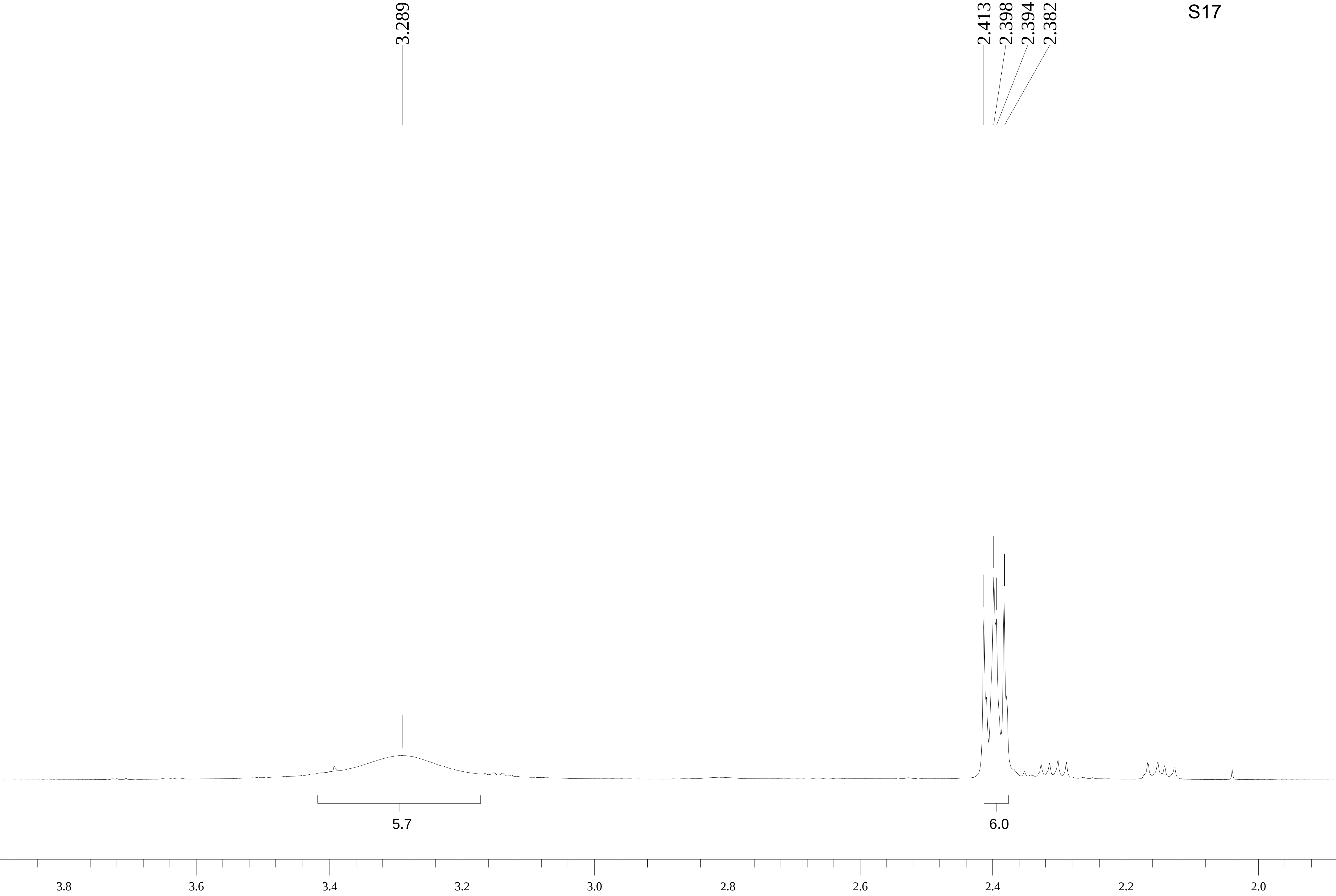
3.289
2.413
2.398
2.394
2.382
5.7
6.0
3.8
3.6
3.4
3.2
3.0
2.8
2.6
2.4
2.2
2.0

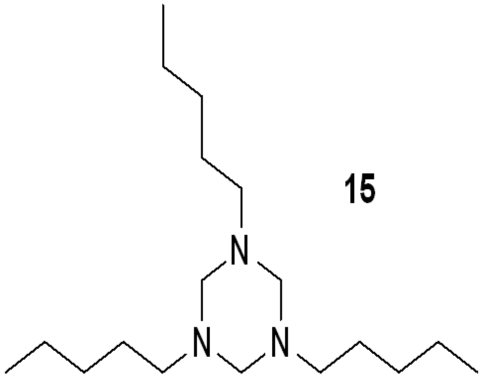
N
N
N

15

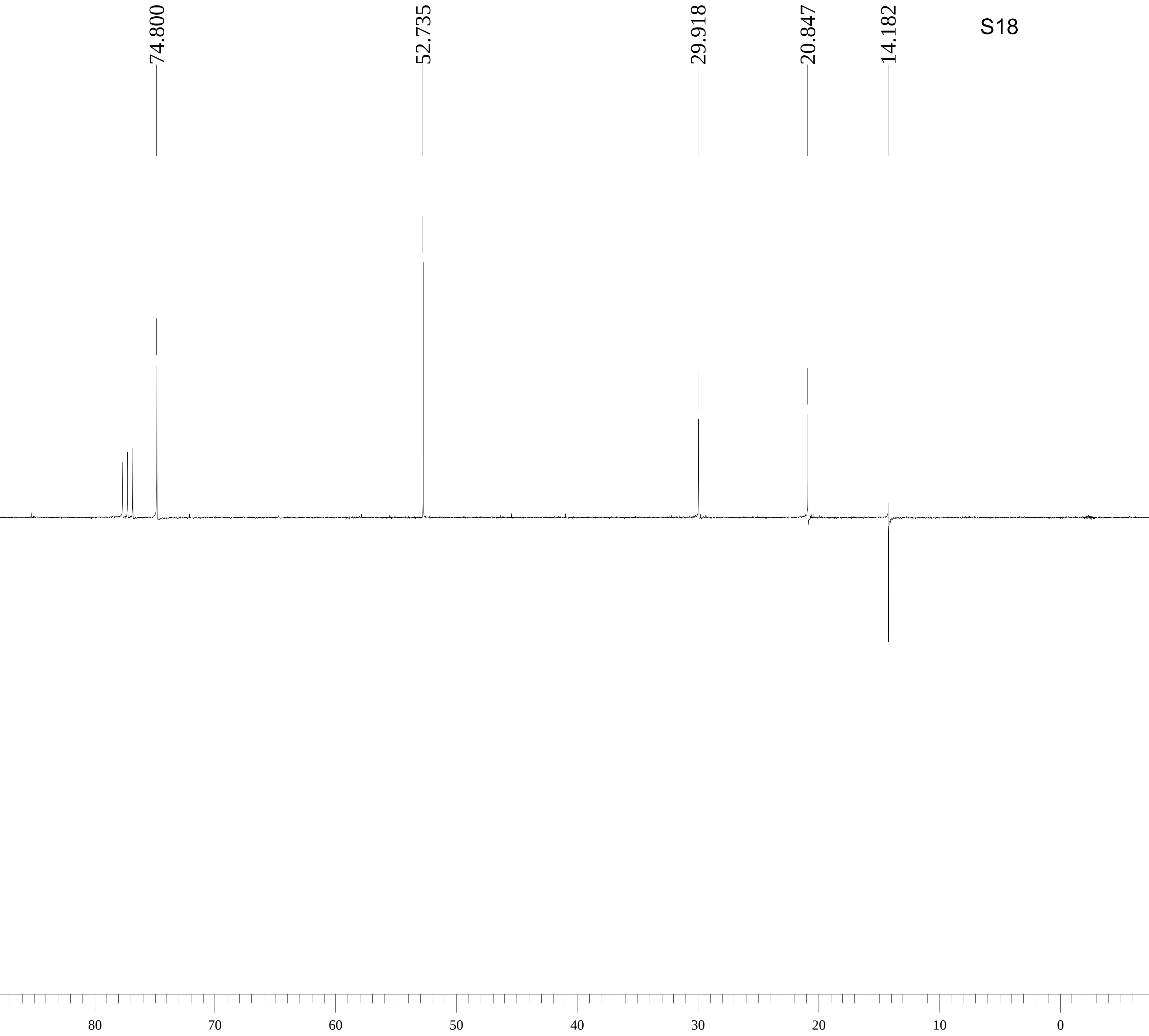
74.800
52.735
29.918
20.847
14.182
110
100
90
80
70
60
50
40
30
20
10
0

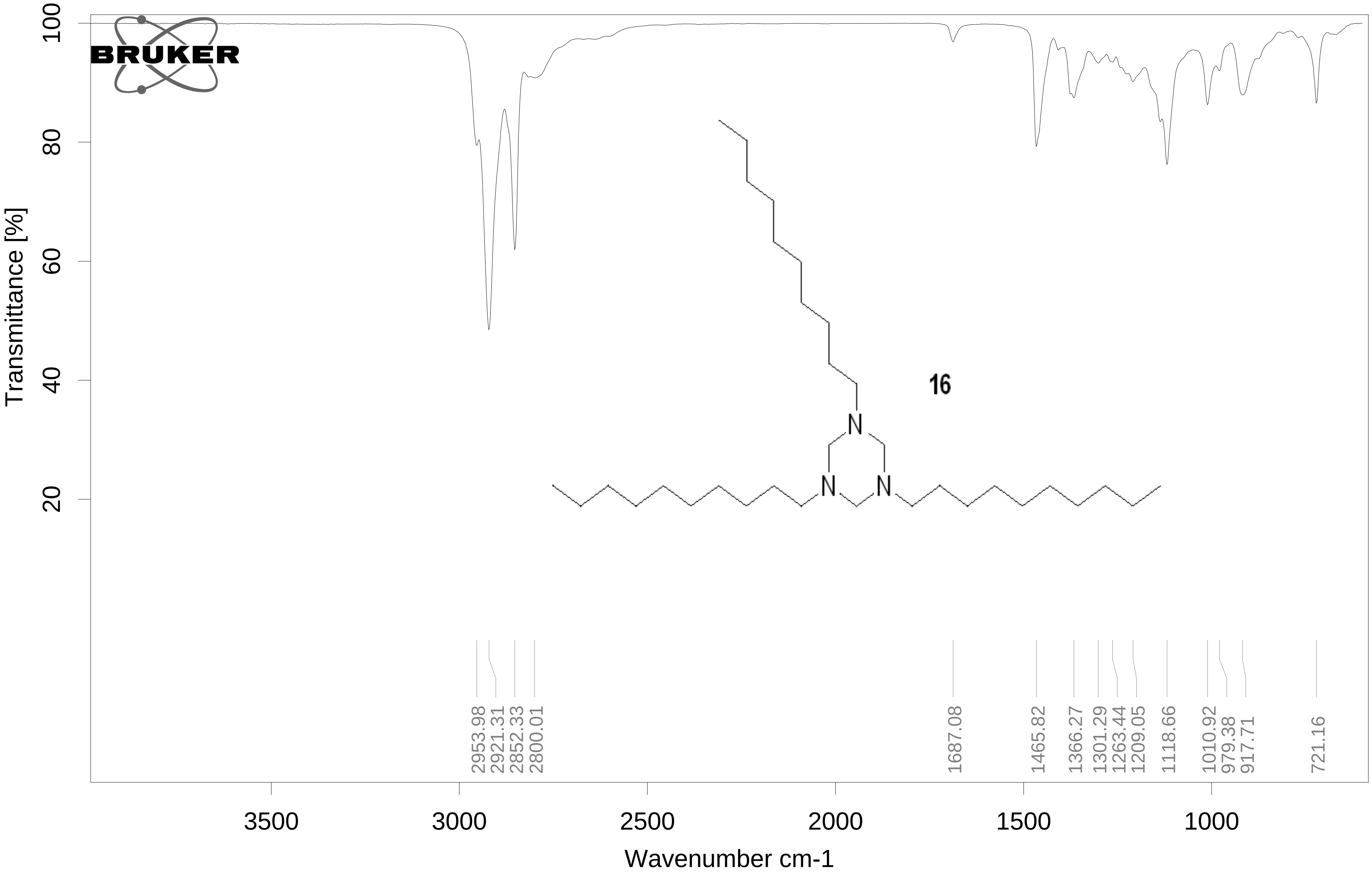

C:\OPUS_7.2.139.1294\MEAS\TDEC.2	TDEC	Instrument type and / or accessory		11/08/2014

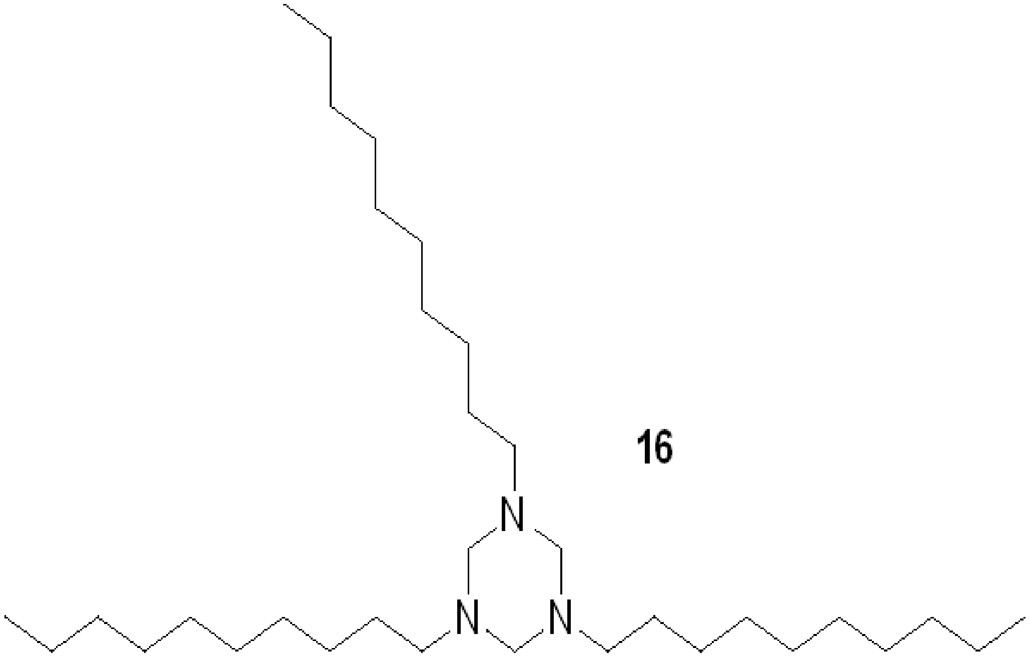

16

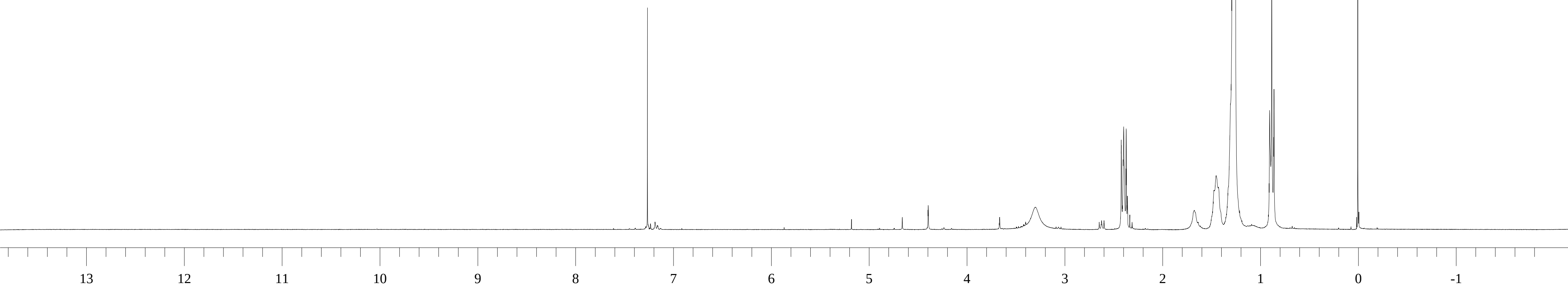

S21

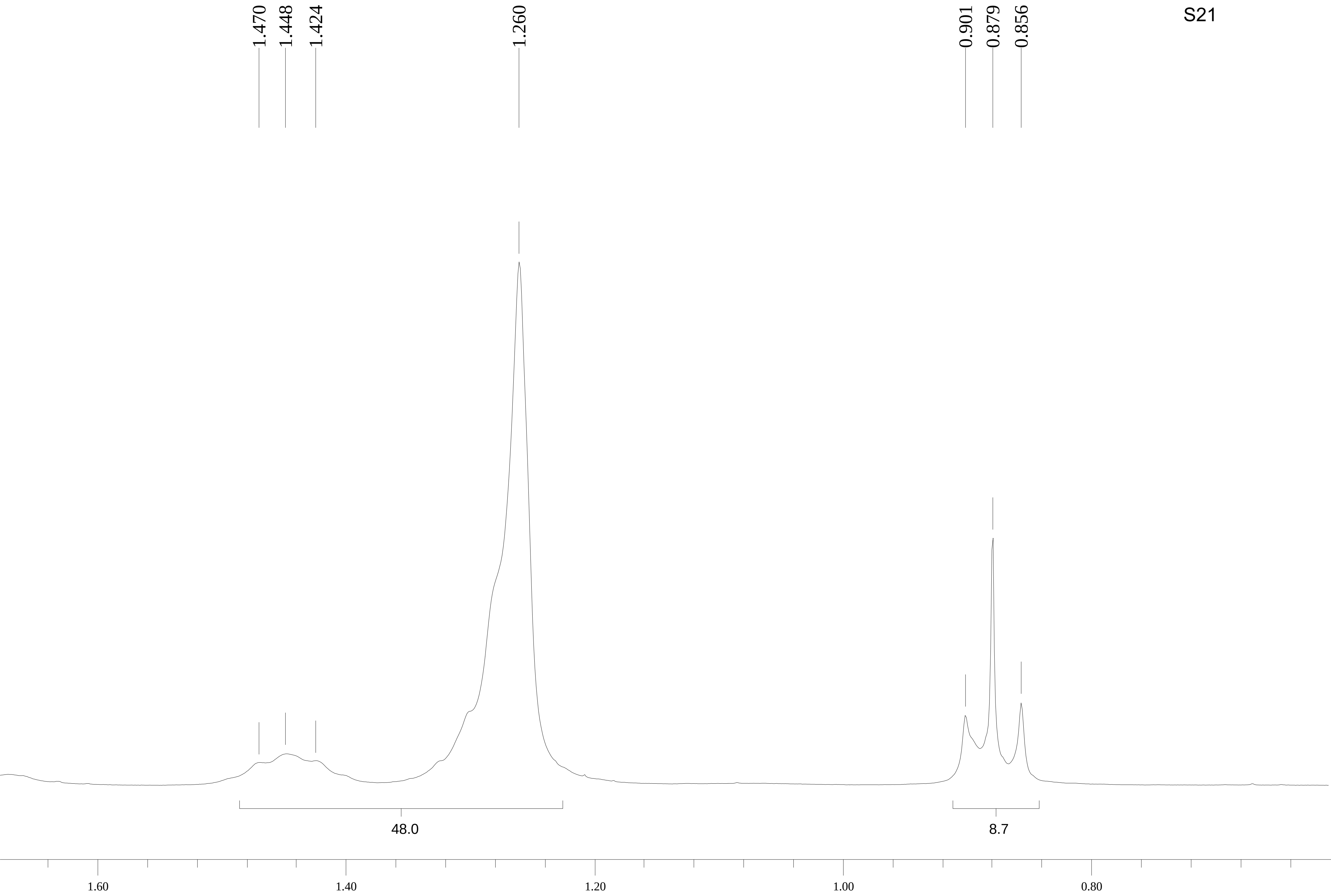

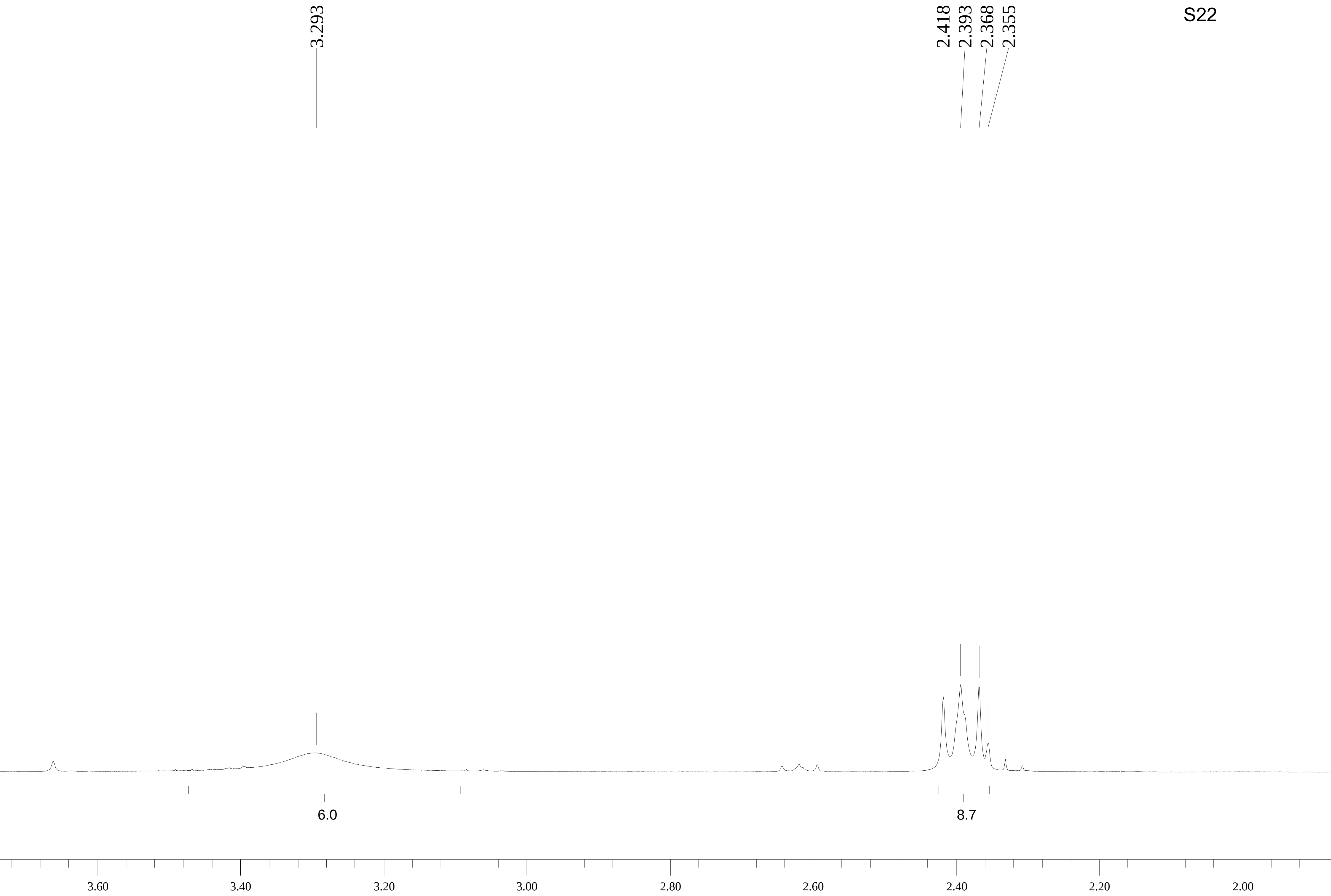
3.293
2.418
2.393
2.368
2.355
6.0
8.7
3.60
3.40
3.20
3.00
2.80
2.60
2.40
2.20
2.00

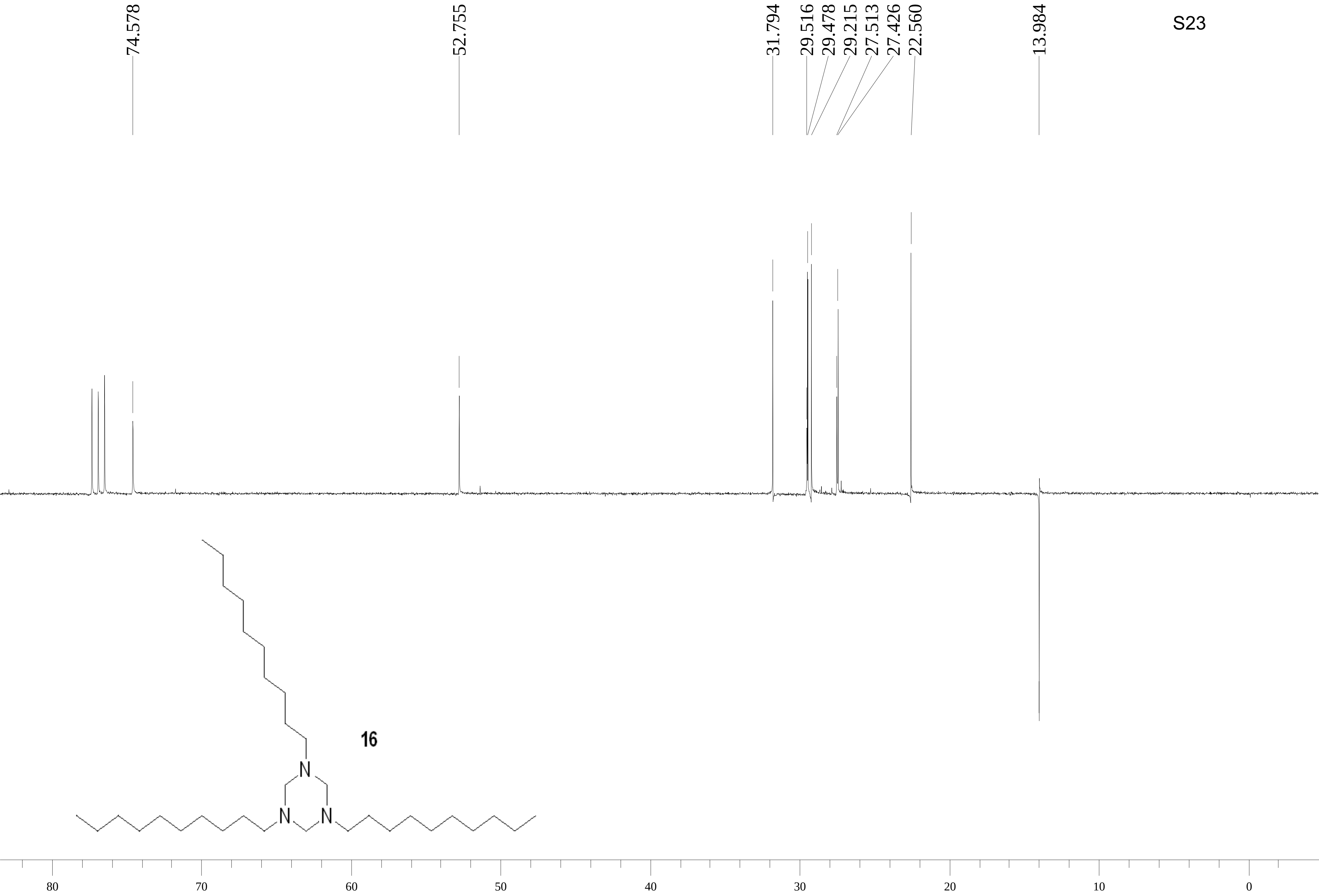
74.578
52.755
31.794
29.516
29.478
29.215
27.513
27.426
22.560
13.984
N
N
N
80
70
60
50
40
30
20
10
0

16

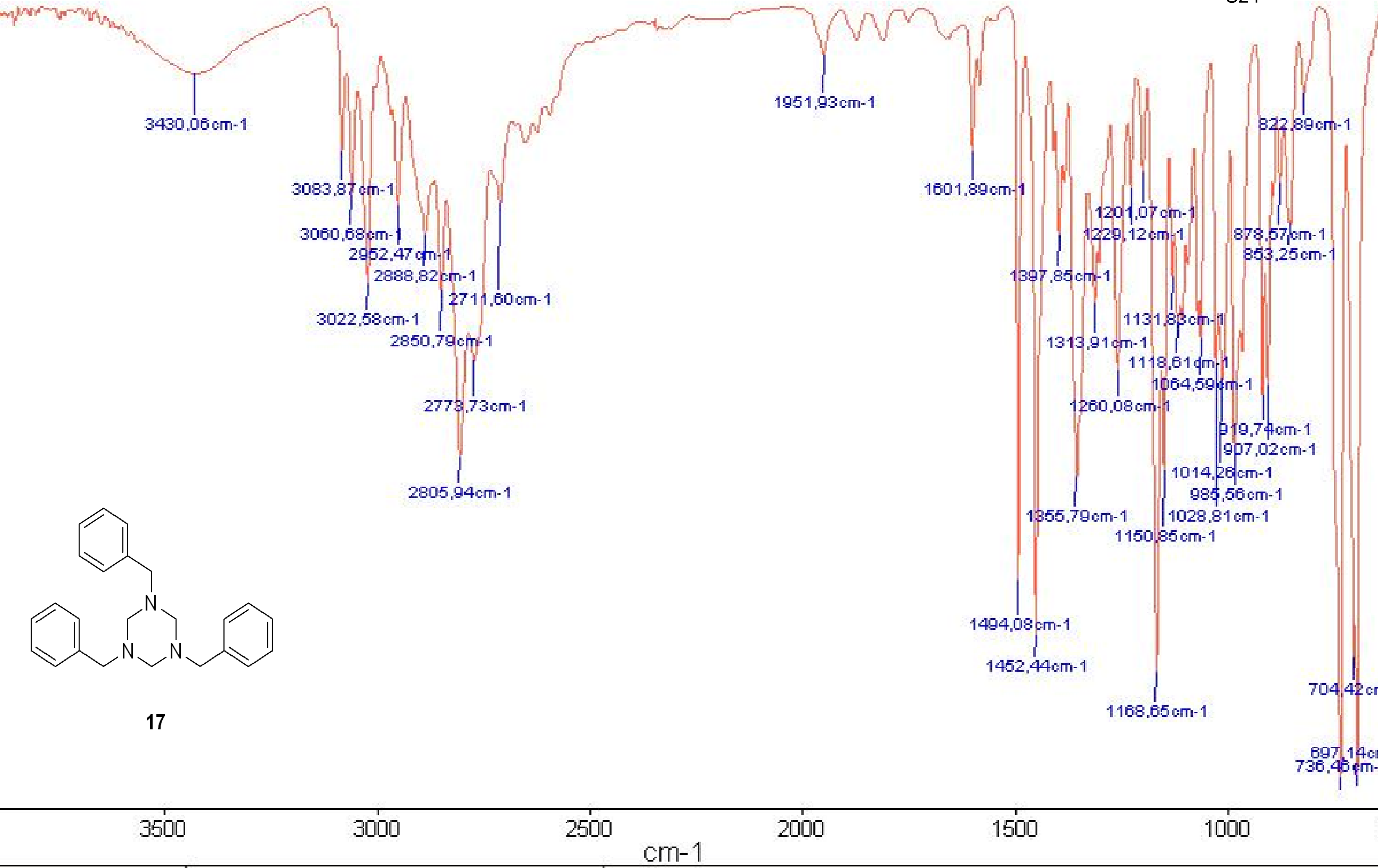

17

	Descrição	
Op. Vania_1	Marcelo	

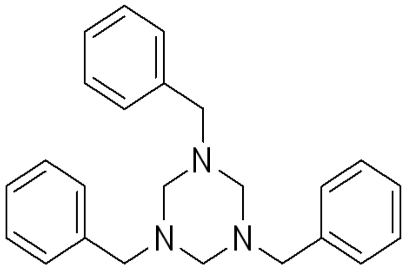

17

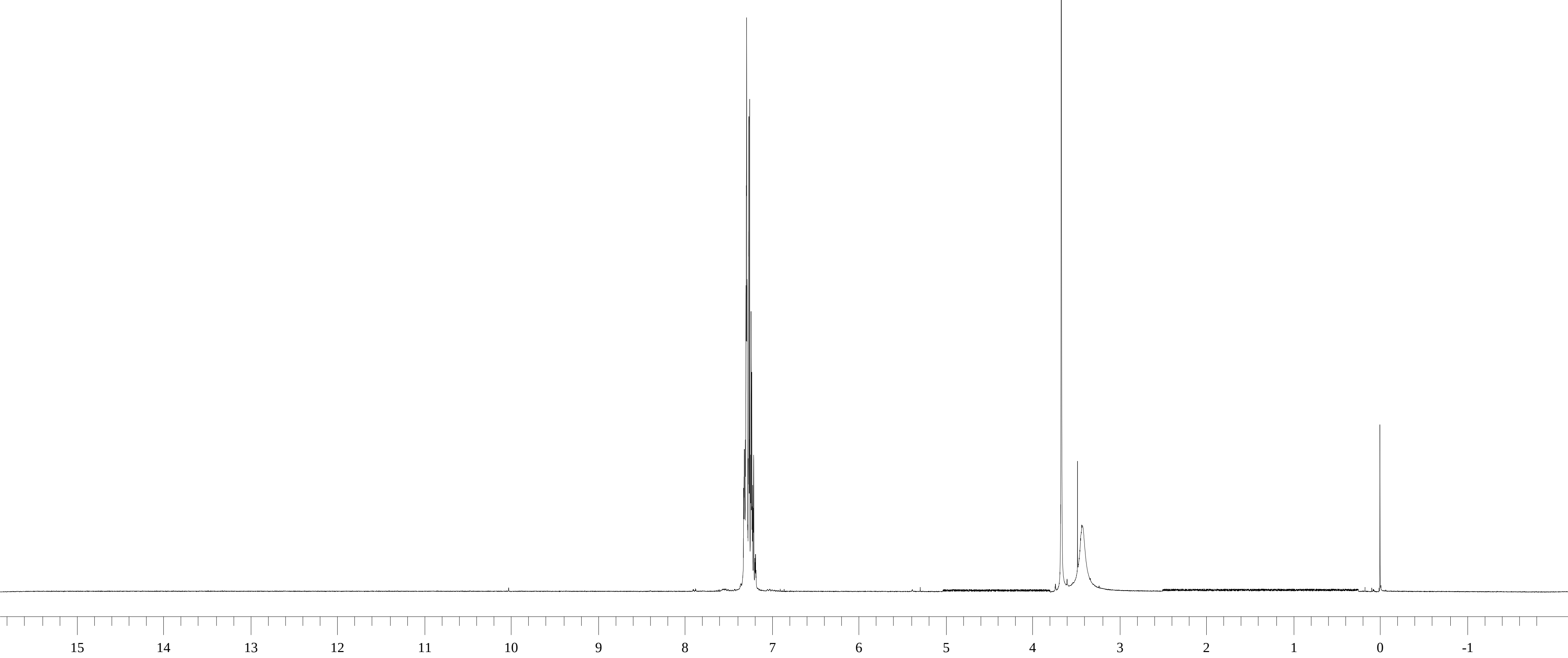

S26

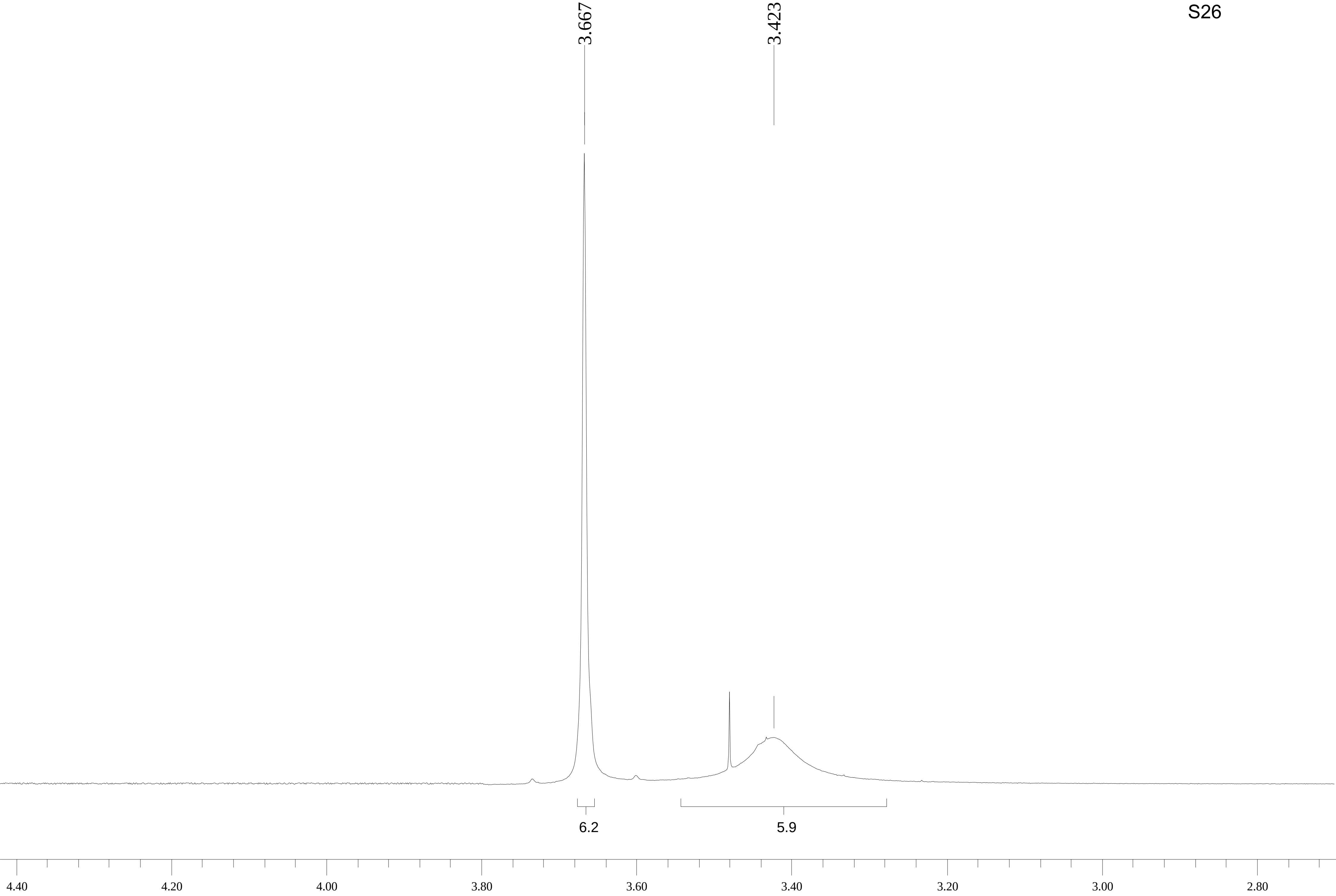

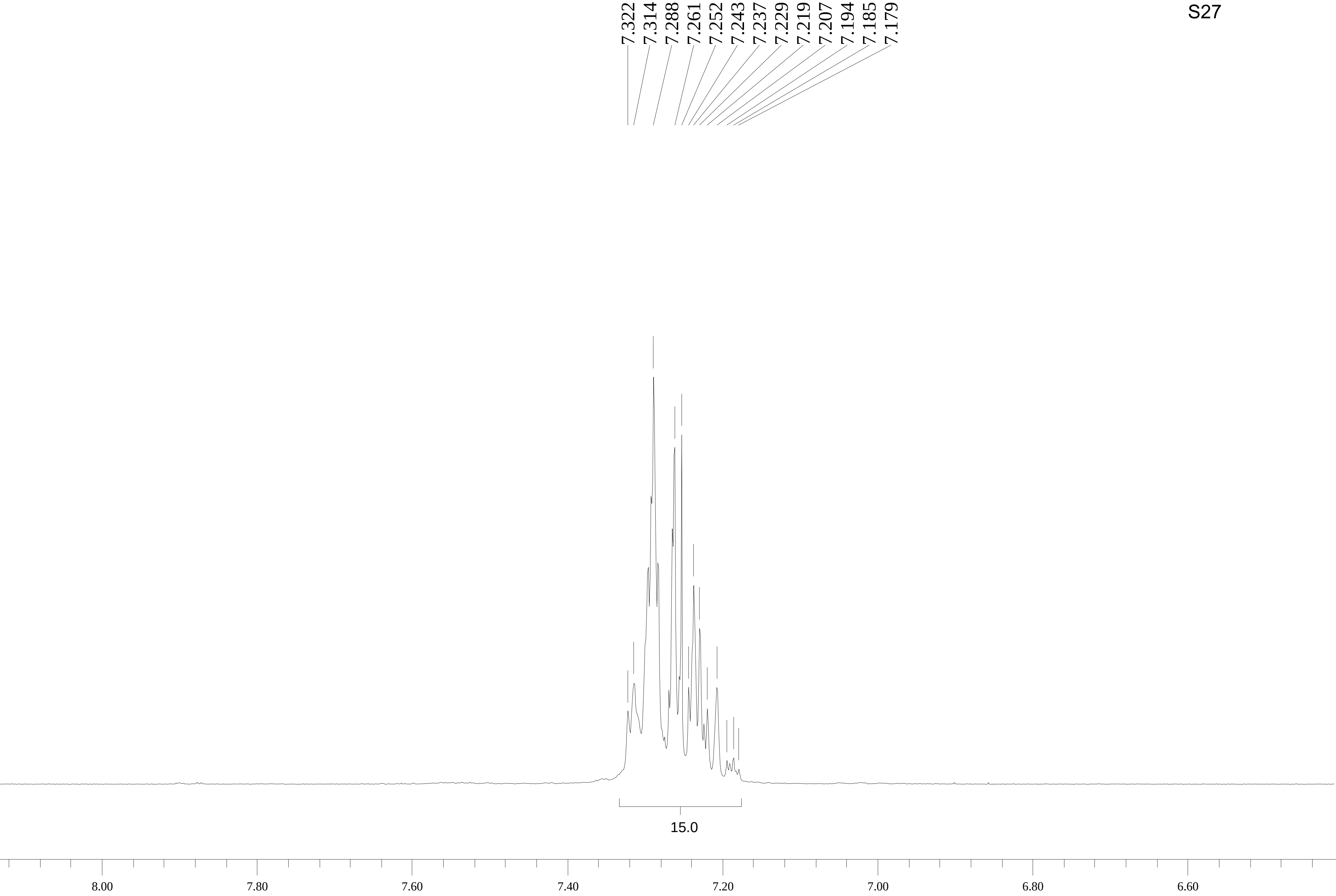
7.322
7.314
7.288
7.261
7.252
7.243
7.237
7.229
7.219
7.207
7.194
7.185
7.179
15.0
8.00
7.80
7.60
7.40
7.20
7.00
6.80
6.60

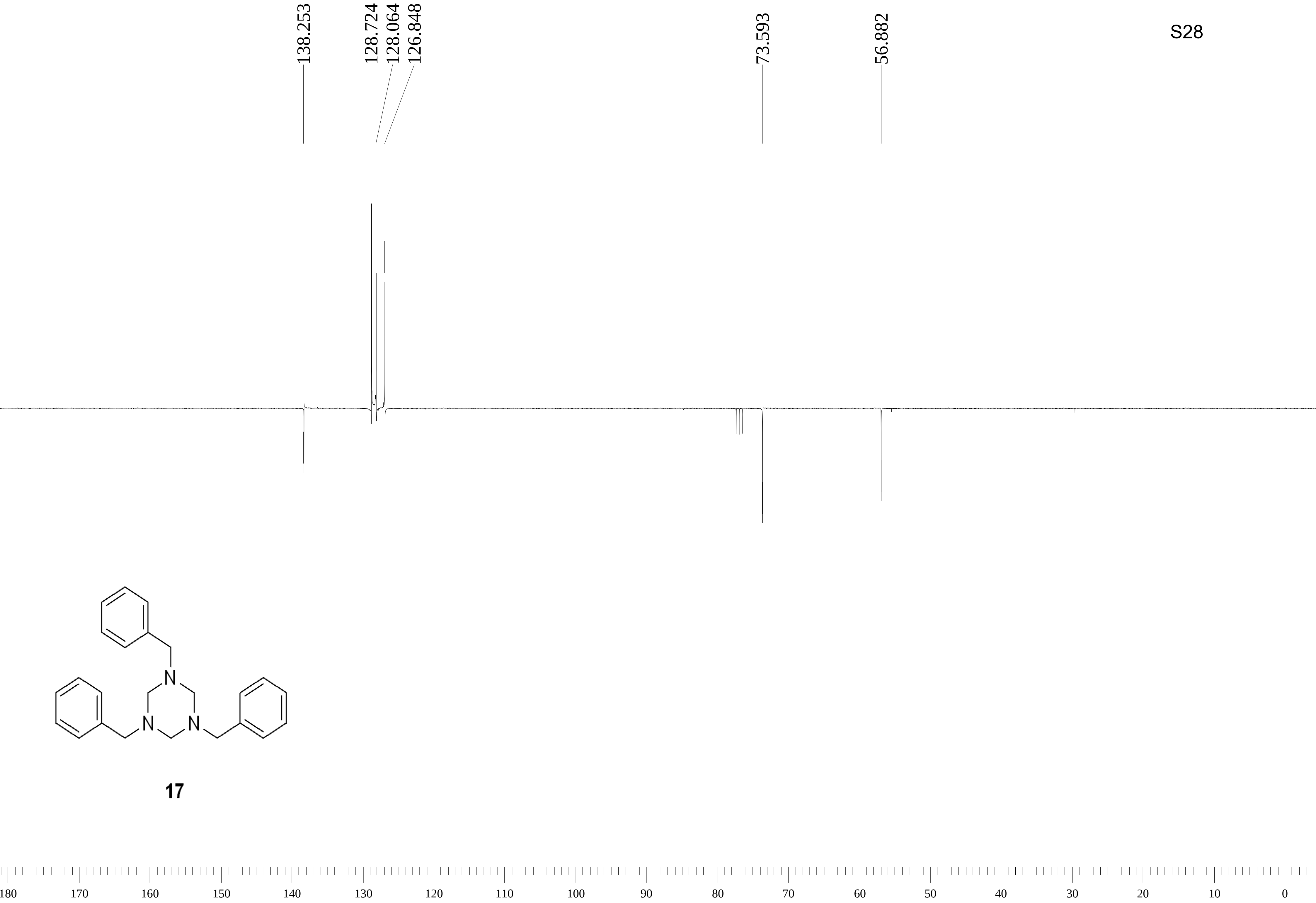

138.253
128.724
128.064
126.848
73.593
56.882
N
N
N
180
170
160
150
140
130
120
110
100
90
80
70
60
50
40
30
20
10
0

17

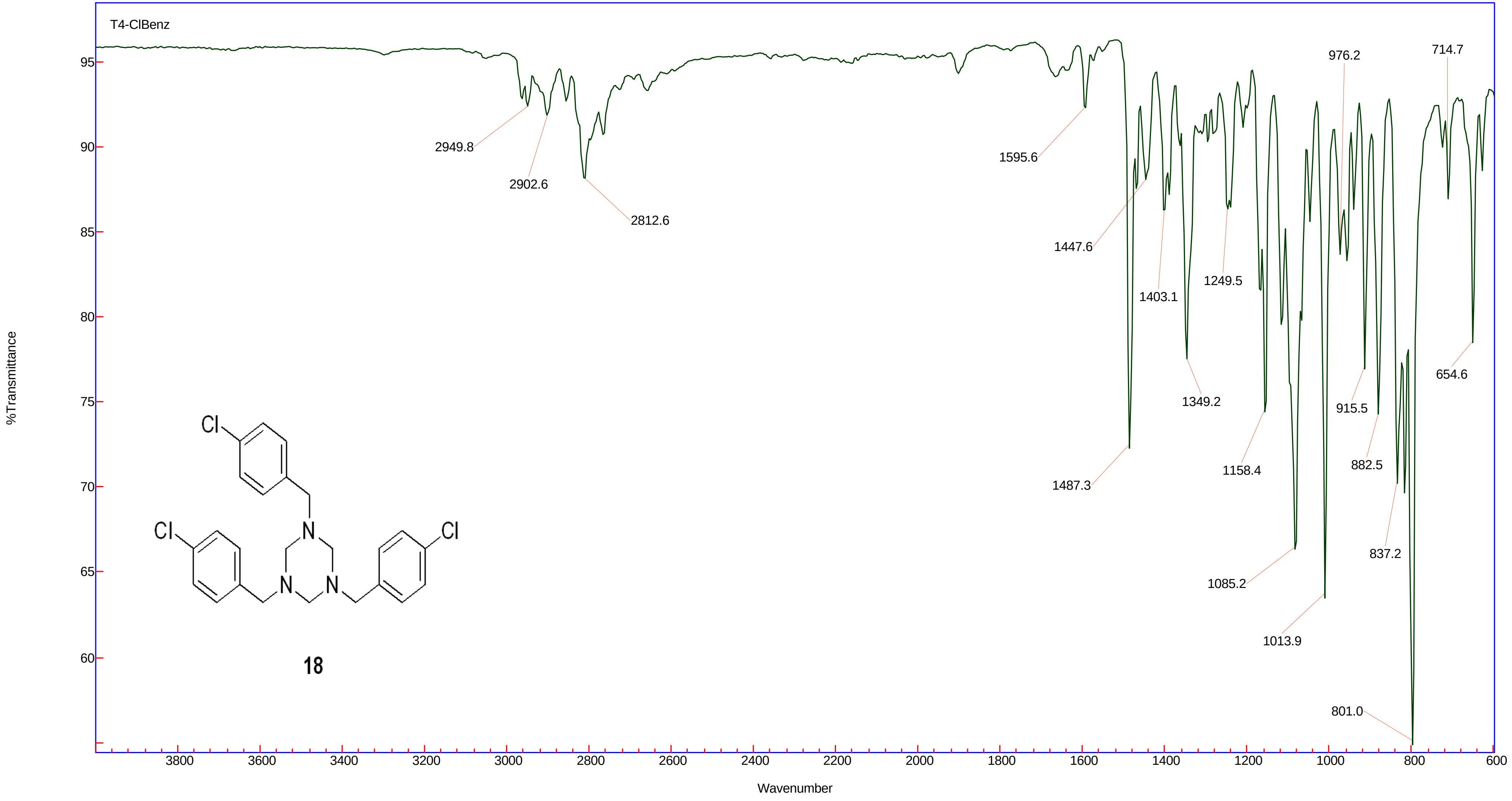

Name
T4-ClBenz

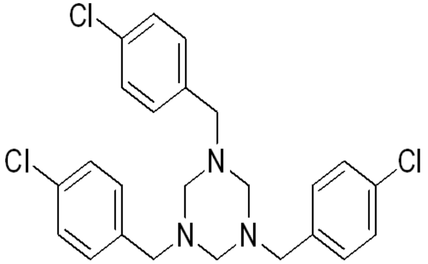

18

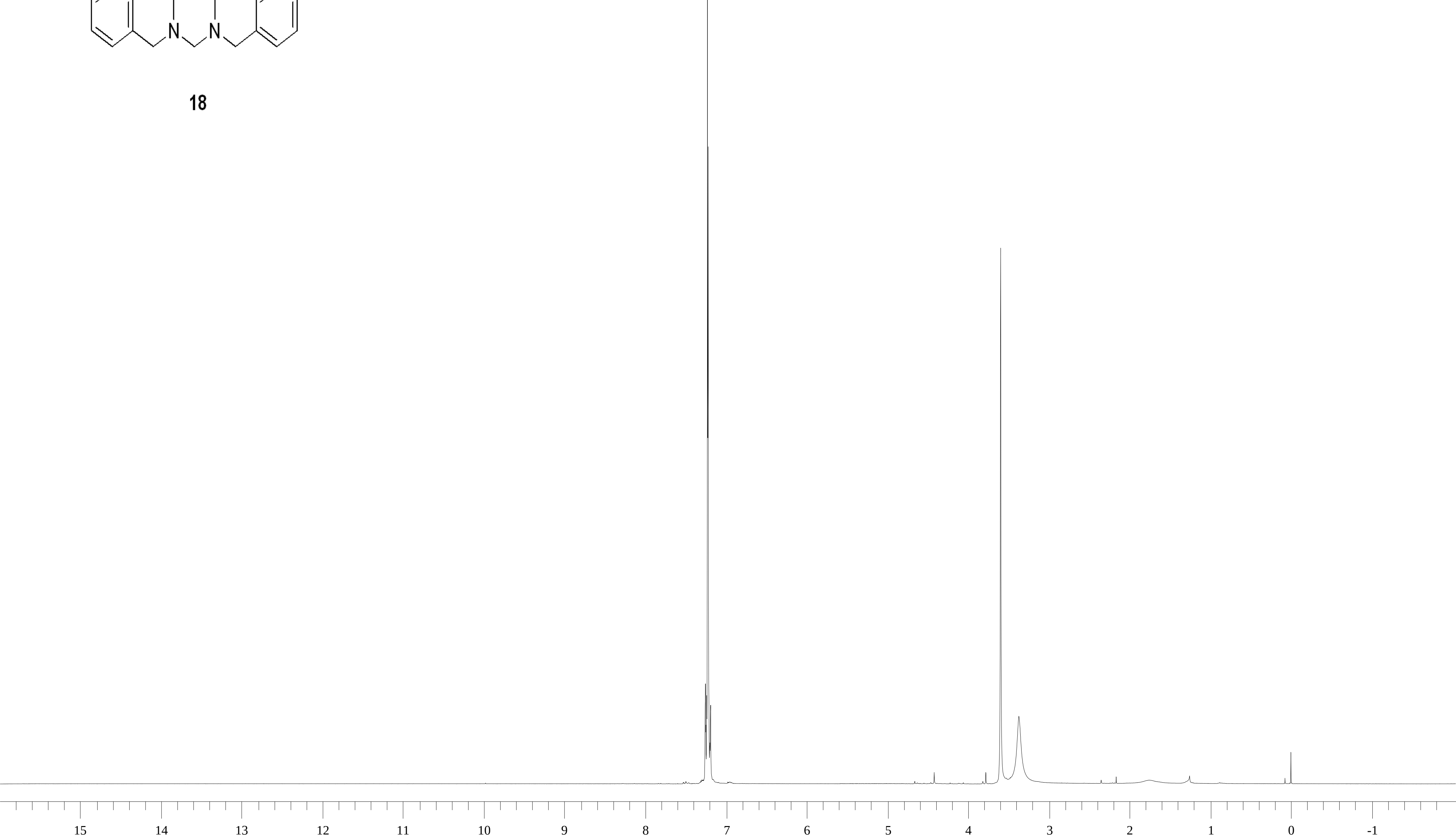

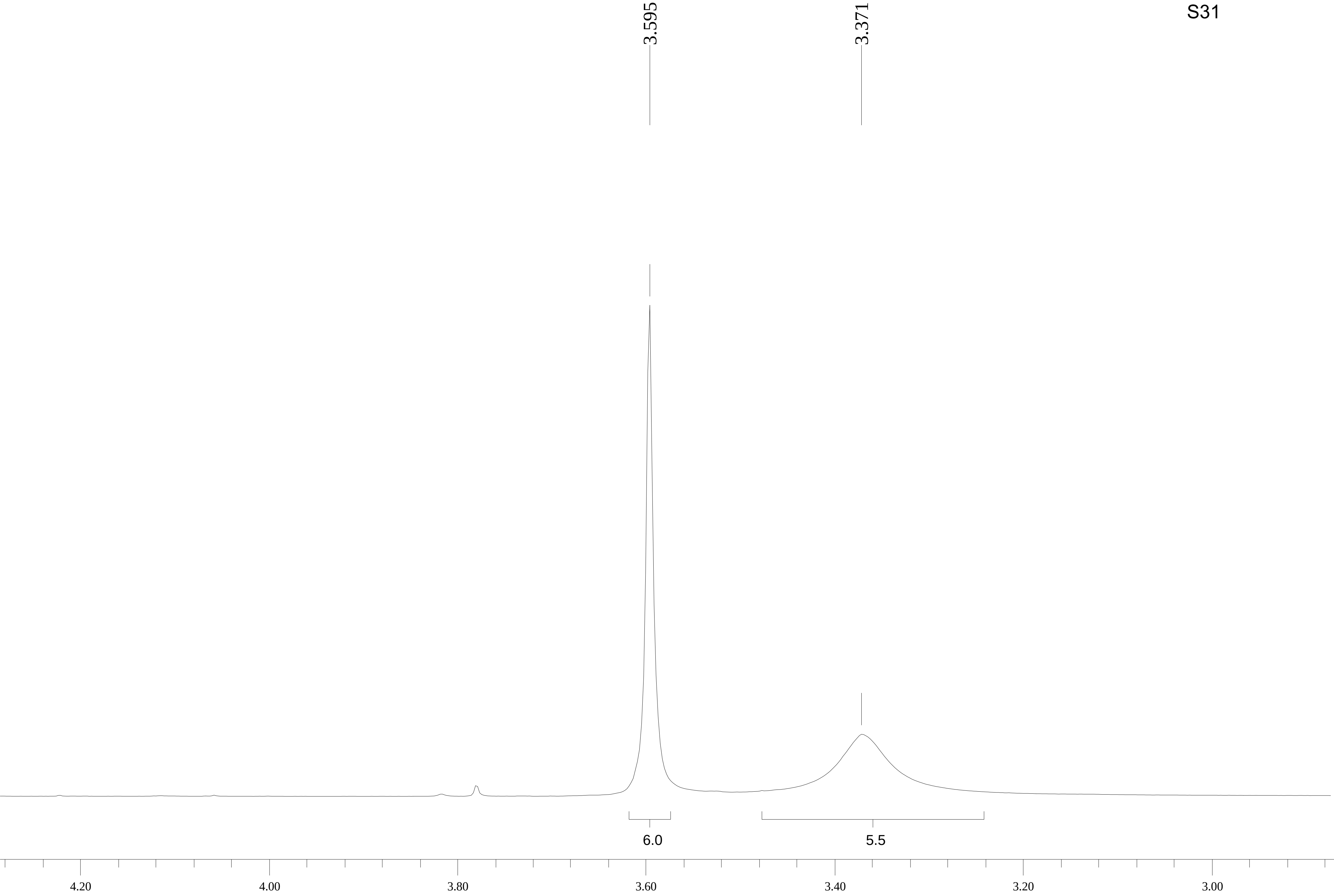
3.595
3.371
6.0
5.5
4.20
4.00
3.80
3.60
3.40
3.20
3.00

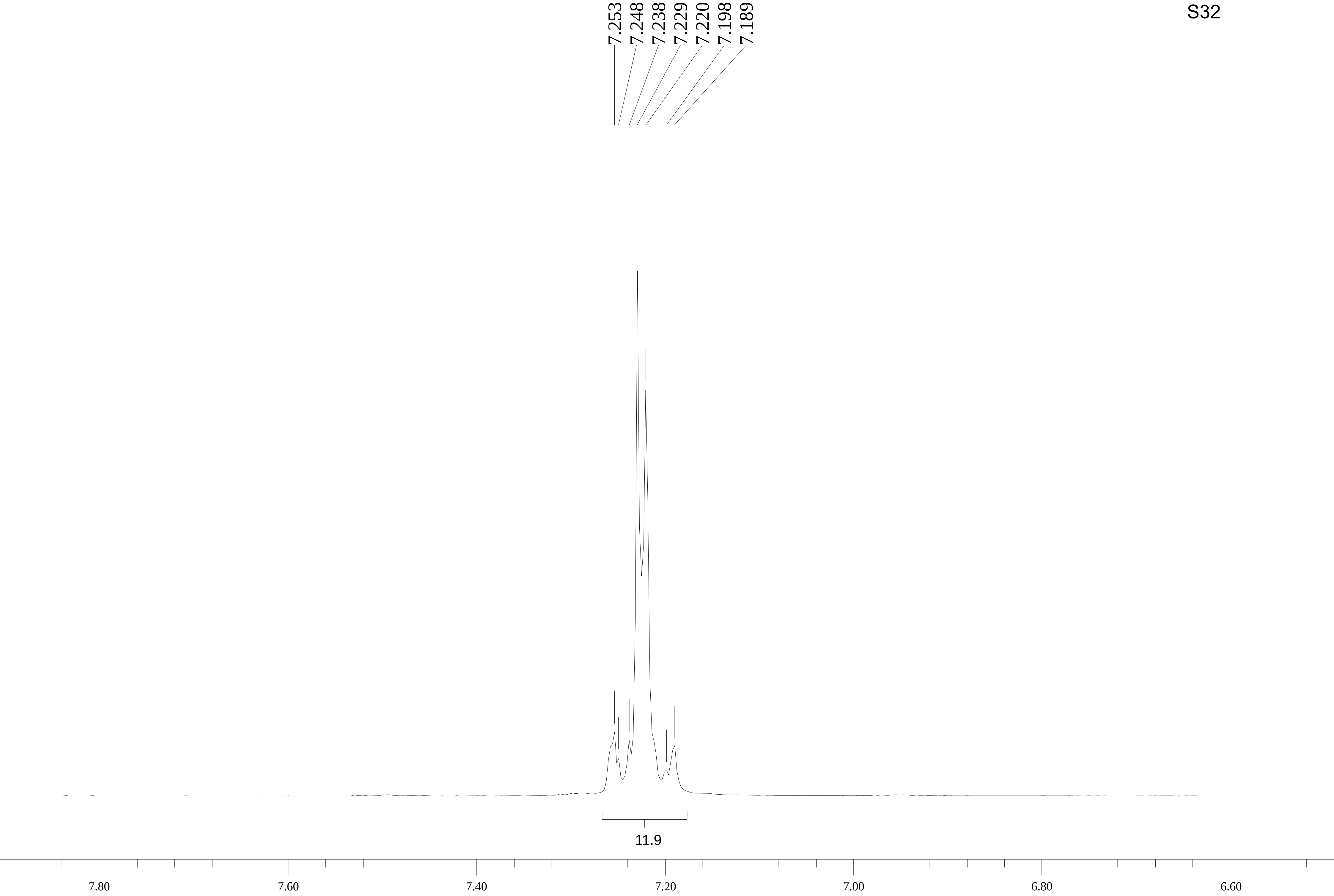
7.253
7.248
7.238
7.229
7.220
7.198
7.189
11.9
7.80
7.60
7.40
7.20
7.00
6.80
6.60

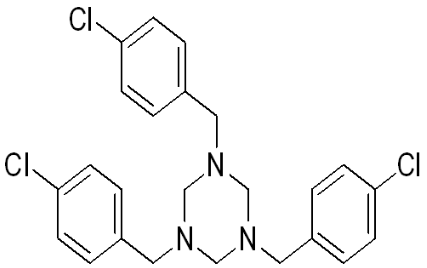

18

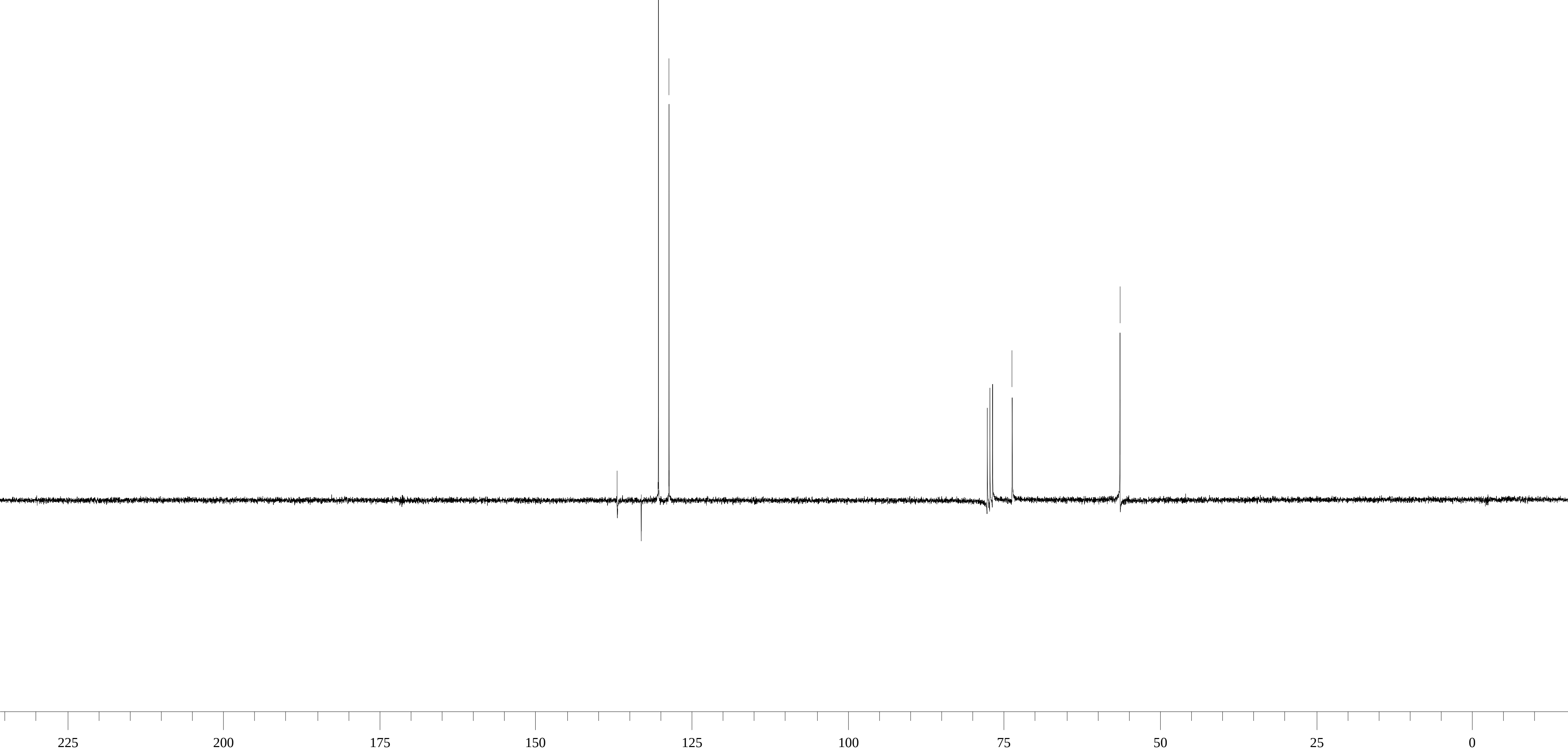
136.868
133.057
130.301
128.598
73.647
56.379
225
200
175
150
125
100
75
50
25
0

Agilent Resolutions Pro

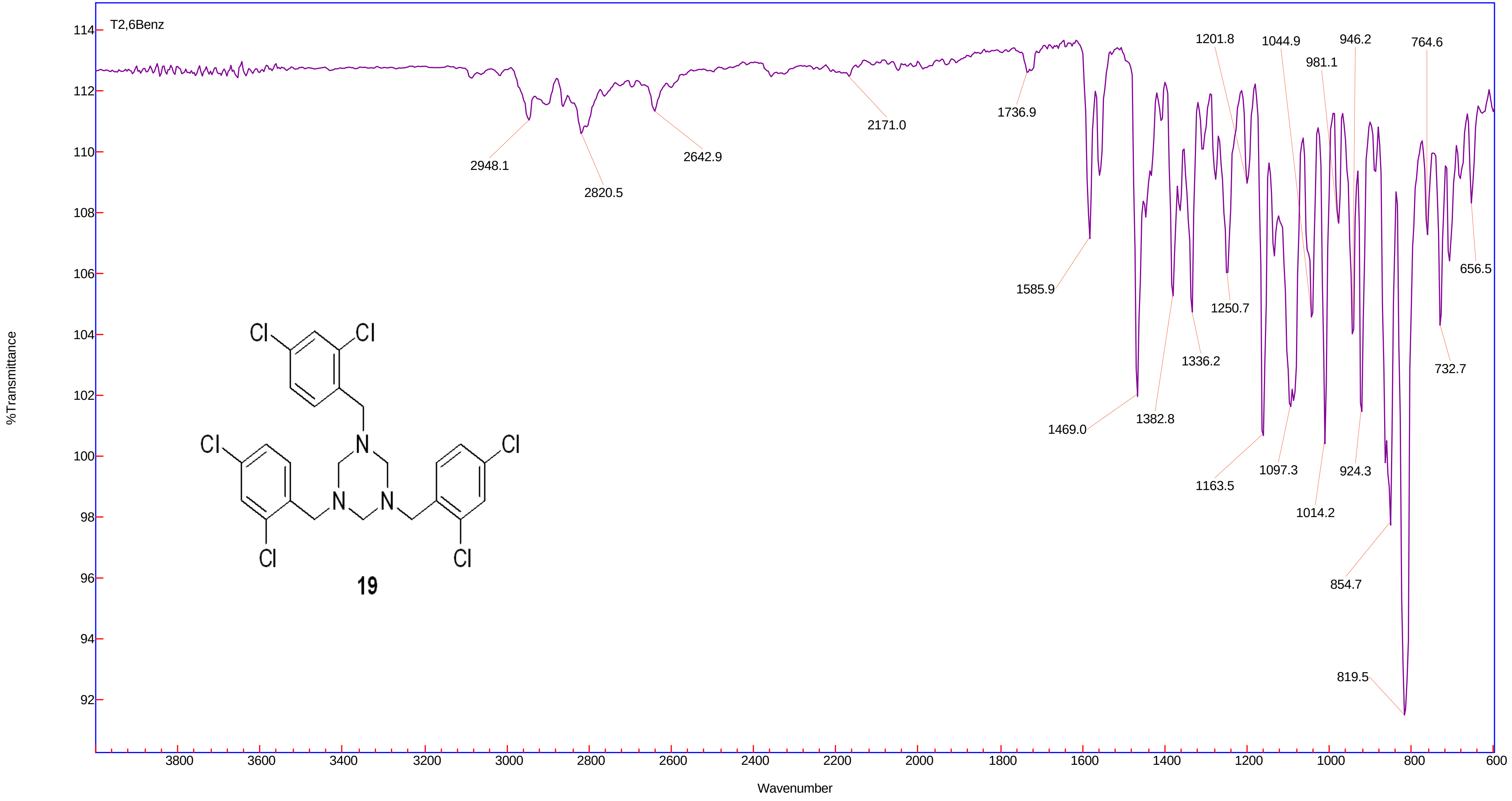

Name	
T2,6Benz	

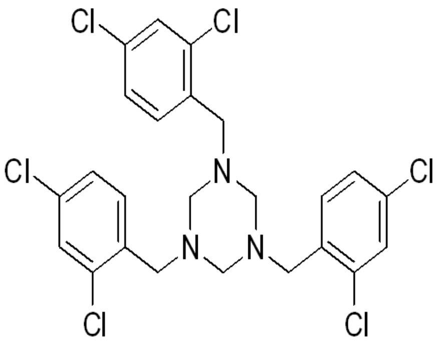

19

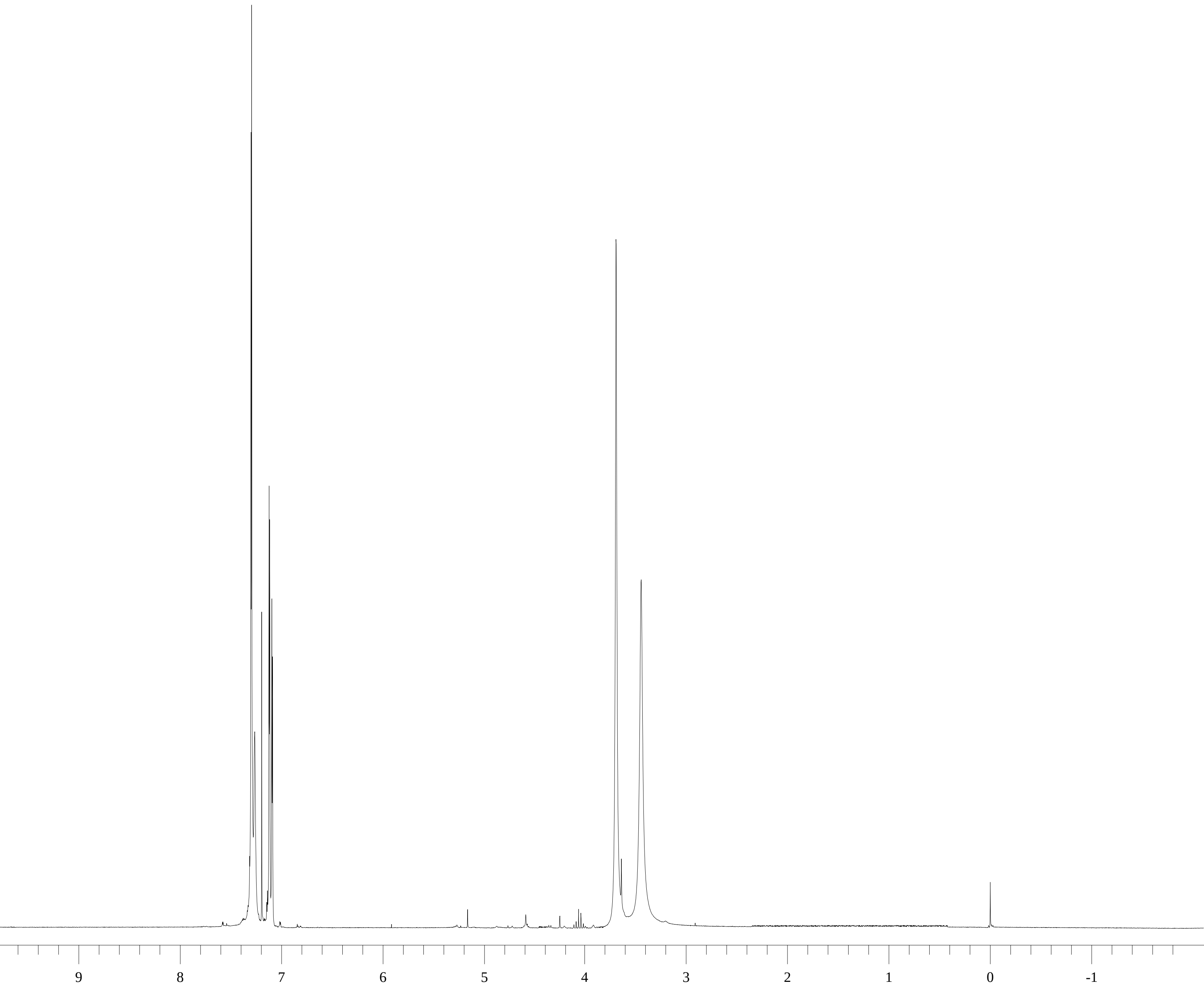

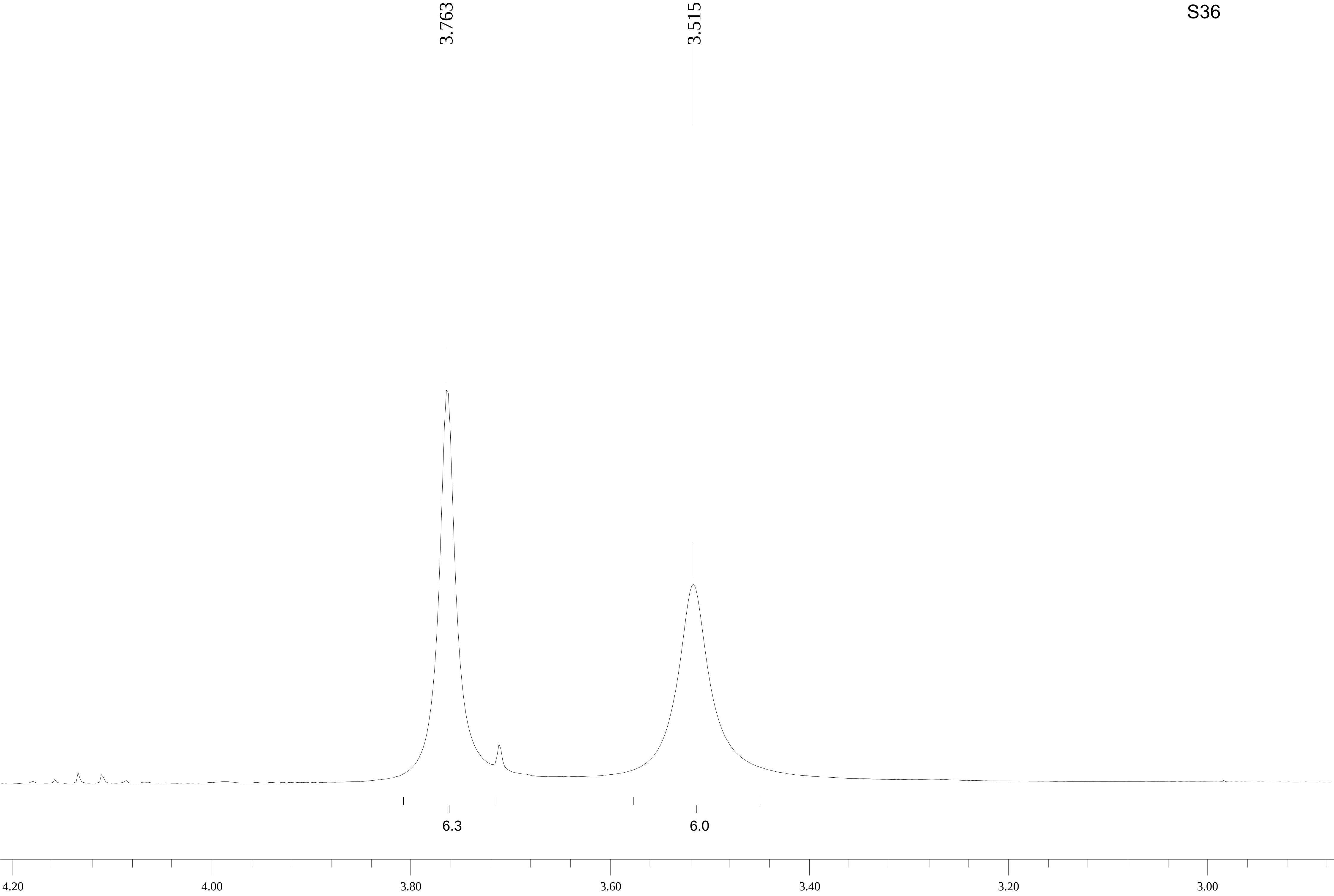
3.763
3.515
6.3
6.0
4.20
4.00
3.80
3.60
3.40
3.20
3.00

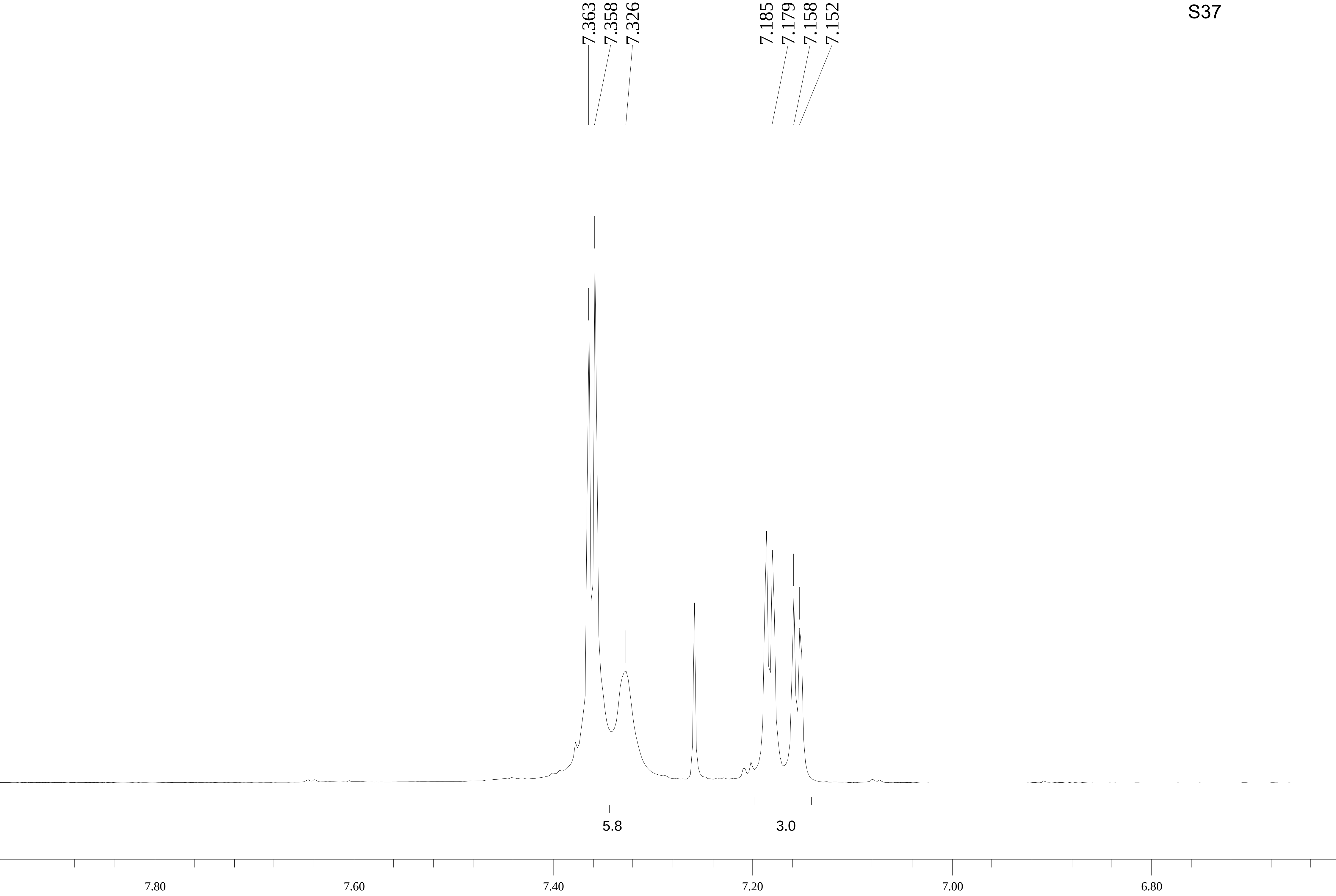
7.363
7.358
7.326
7.185
7.179
7.158
7.152
5.8
3.0
7.80
7.60
7.40
7.20
7.00
6.80

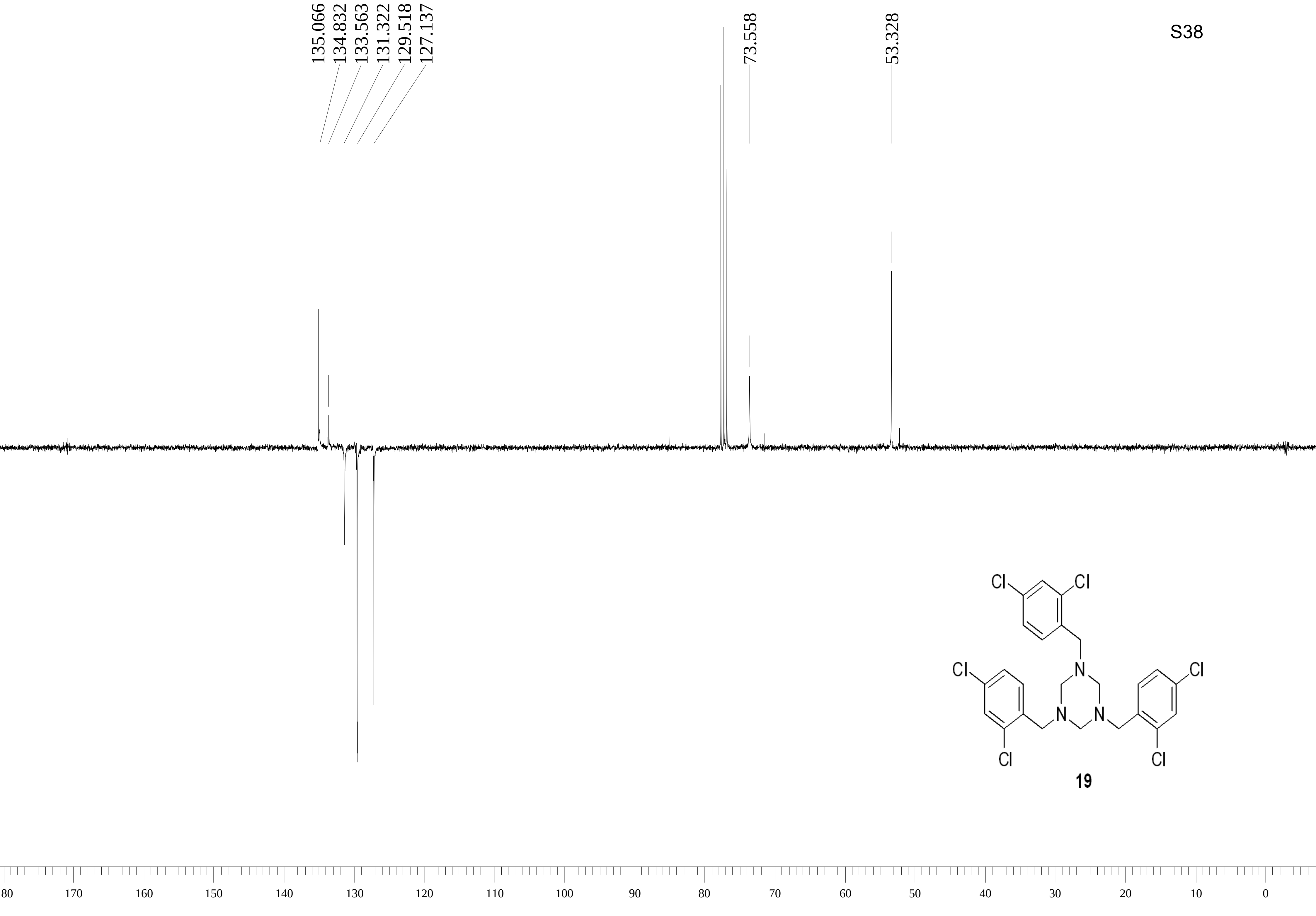
135.066
134.832
133.563
131.322
129.518
127.137
73.558
53.328
80
170
160
150
140
130
120
110
100
90
80
70
60
50
40
30
20
10
0
Cl
Cl
N
Cl
Cl
N
N
Cl
Cl

19

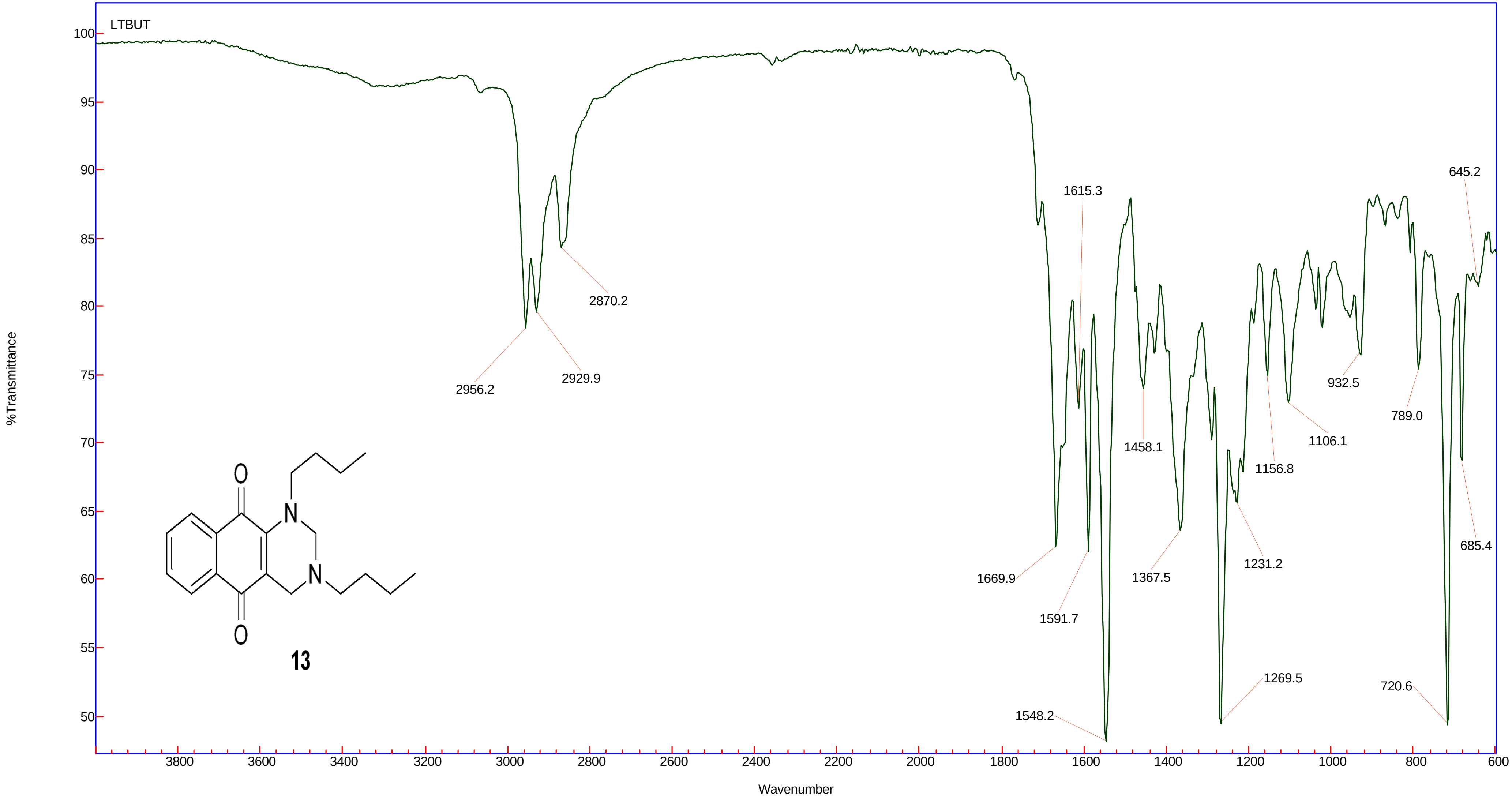
LTBUT
%Transmittance
Wavenumber
2956.2
2929.9
2870.2
1669.9
1615.3
1591.7
1548.2
1458.1
1367.5
1269.5
1231.2
1156.8
1106.1
932.5
789.0
720.6
685.4
645.2
13

Name
LTBUT

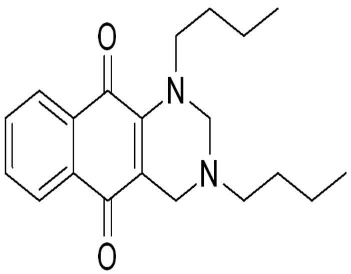

13

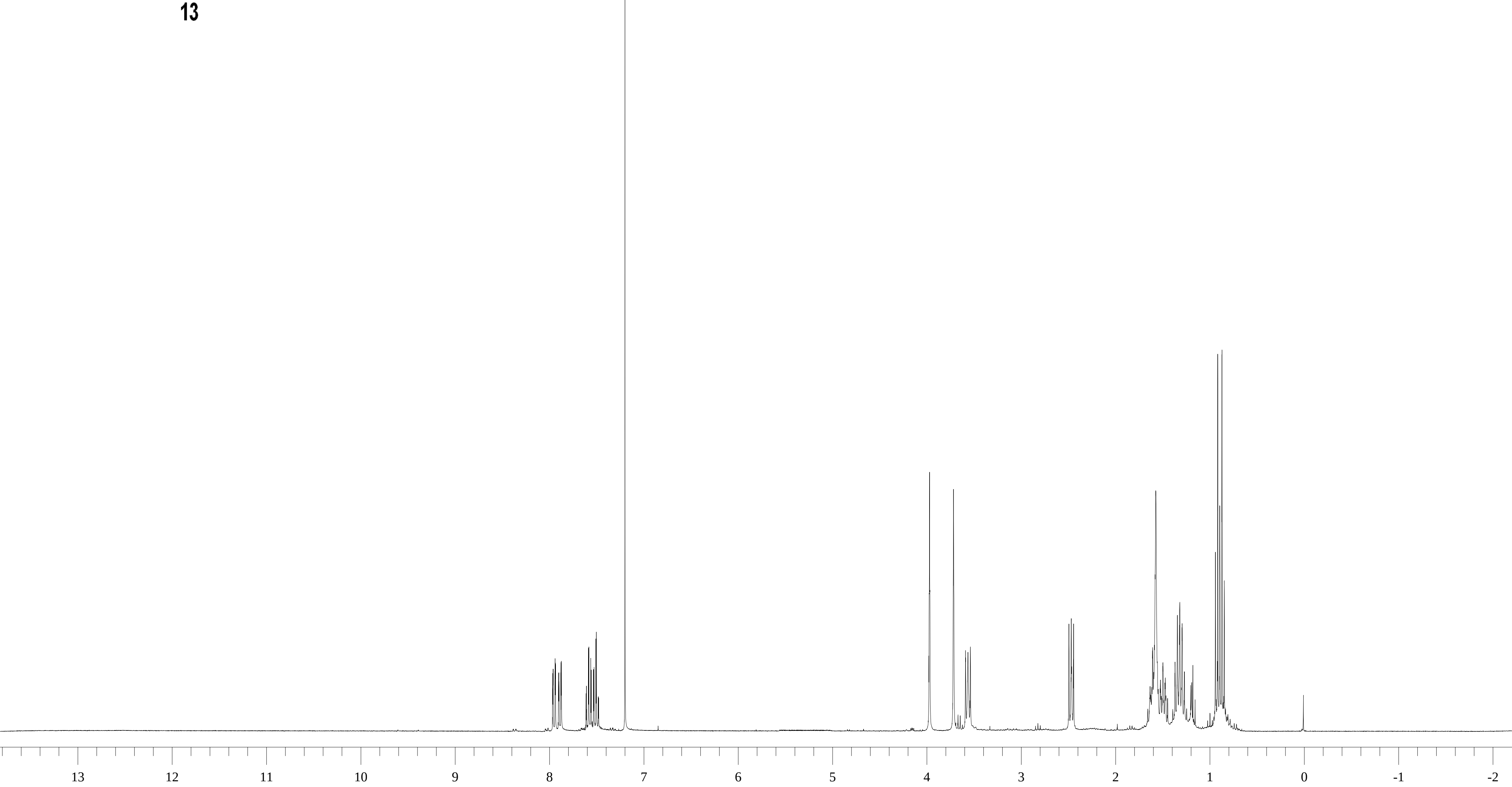

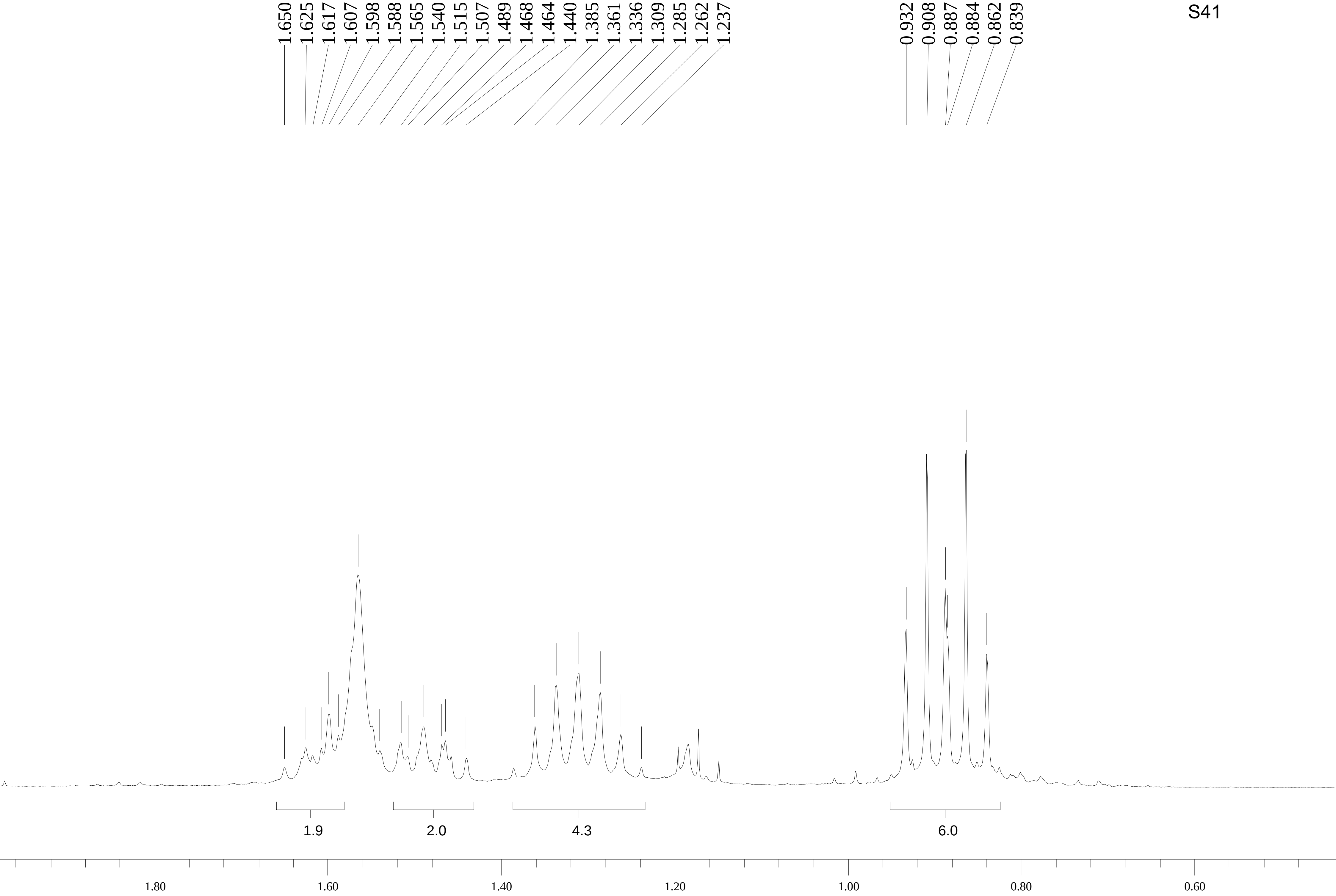
1.650
1.625
1.617
1.607
1.598
1.588
1.565
1.540
1.515
1.507
1.489
1.468
1.464
1.440
1.385
1.361
1.336
1.309
1.285
1.262
1.237
0.932
0.908
0.887
0.884
0.862
0.839
1.9
2.0
4.3
6.0
1.80
1.60
1.40
1.20
1.00
0.80
0.60

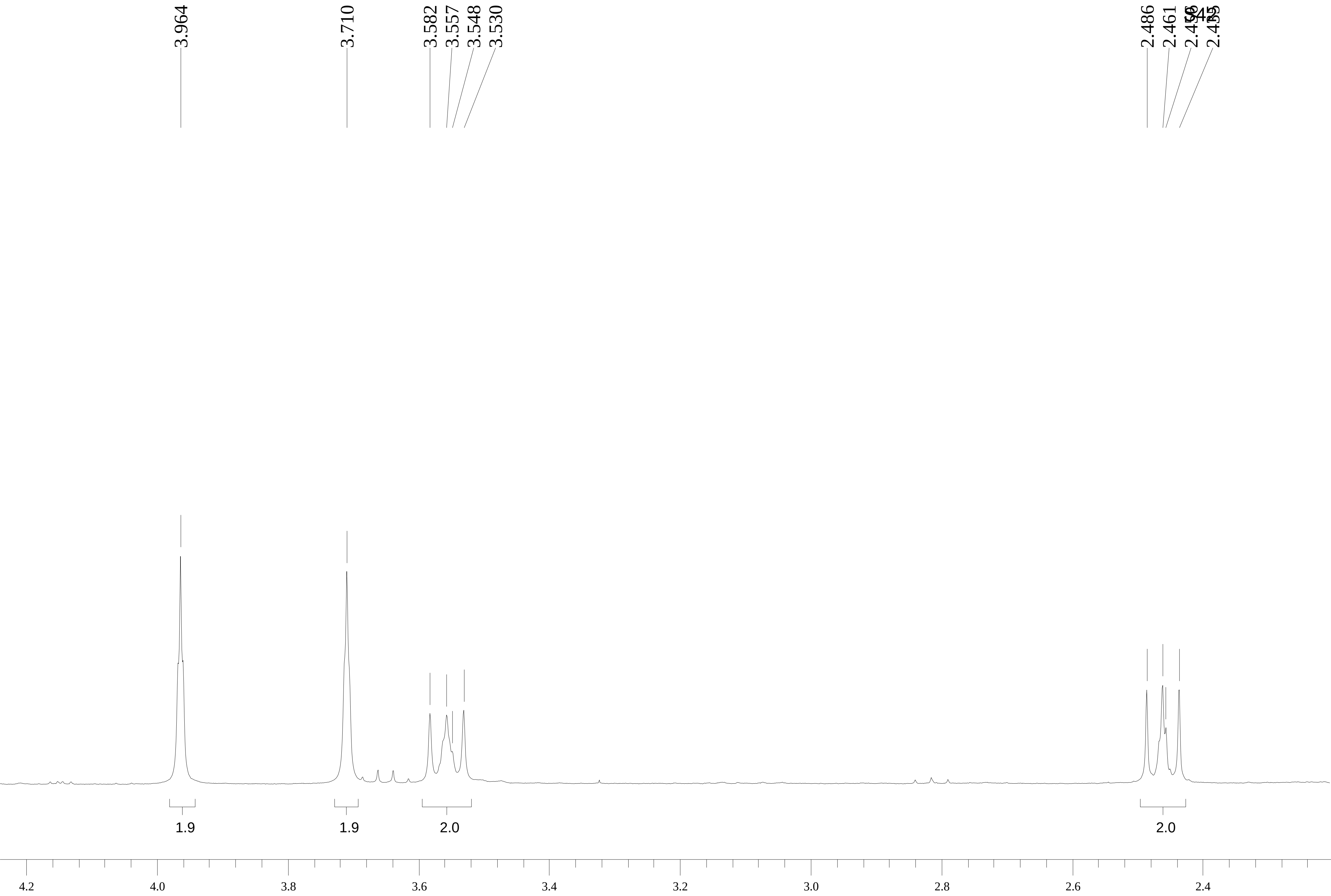
3.964
3.710
3.582
3.557
3.548
3.530
2.486
2.461
1.9
1.9
2.0
2.0
4.2
4.0
3.8
3.6
3.4
3.2
3.0
2.8
2.6
2.4

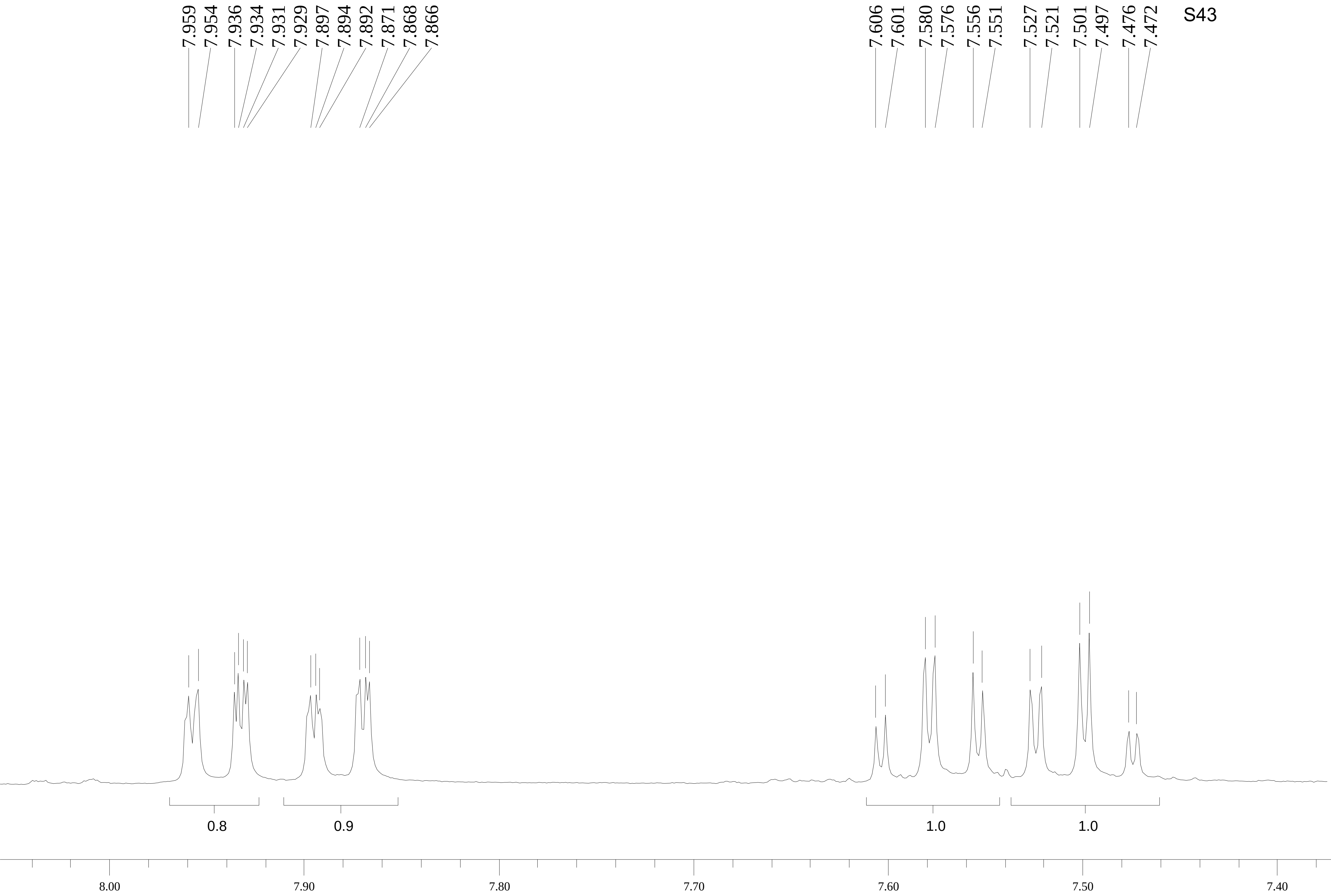

7.959
7.954
7.936
7.934
7.931
7.929
7.897
7.894
7.892
7.871
7.868
7.866
7.606
7.601
7.580
7.576
7.556
7.551
7.527
7.521
7.501
7.497
7.476
7.472
S43
0.8
0.9
1.0
1.0
8.00
7.90
7.80
7.70
7.60
7.50
7.40

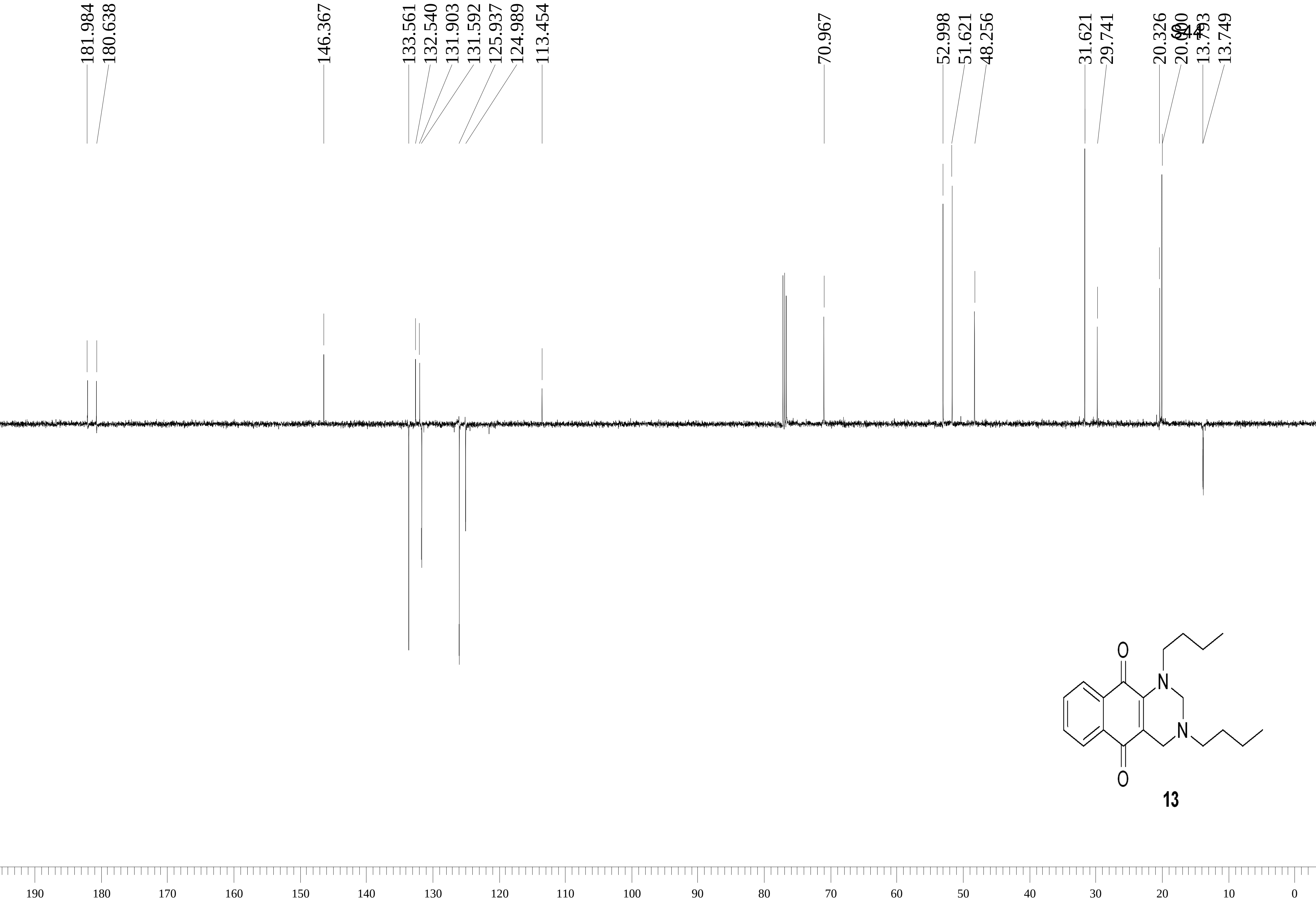

181.984
180.638
146.367
133.561
132.540
131.903
131.592
125.937
124.989
113.454
70.967
52.998
51.621
48.256
31.621
29.741
20.326
13.749
O
N
N
O
190
180
170
160
150
140
130
120
110
100
90
80
70
60
50
40
30
20
10
0

13

CIO

S45

13

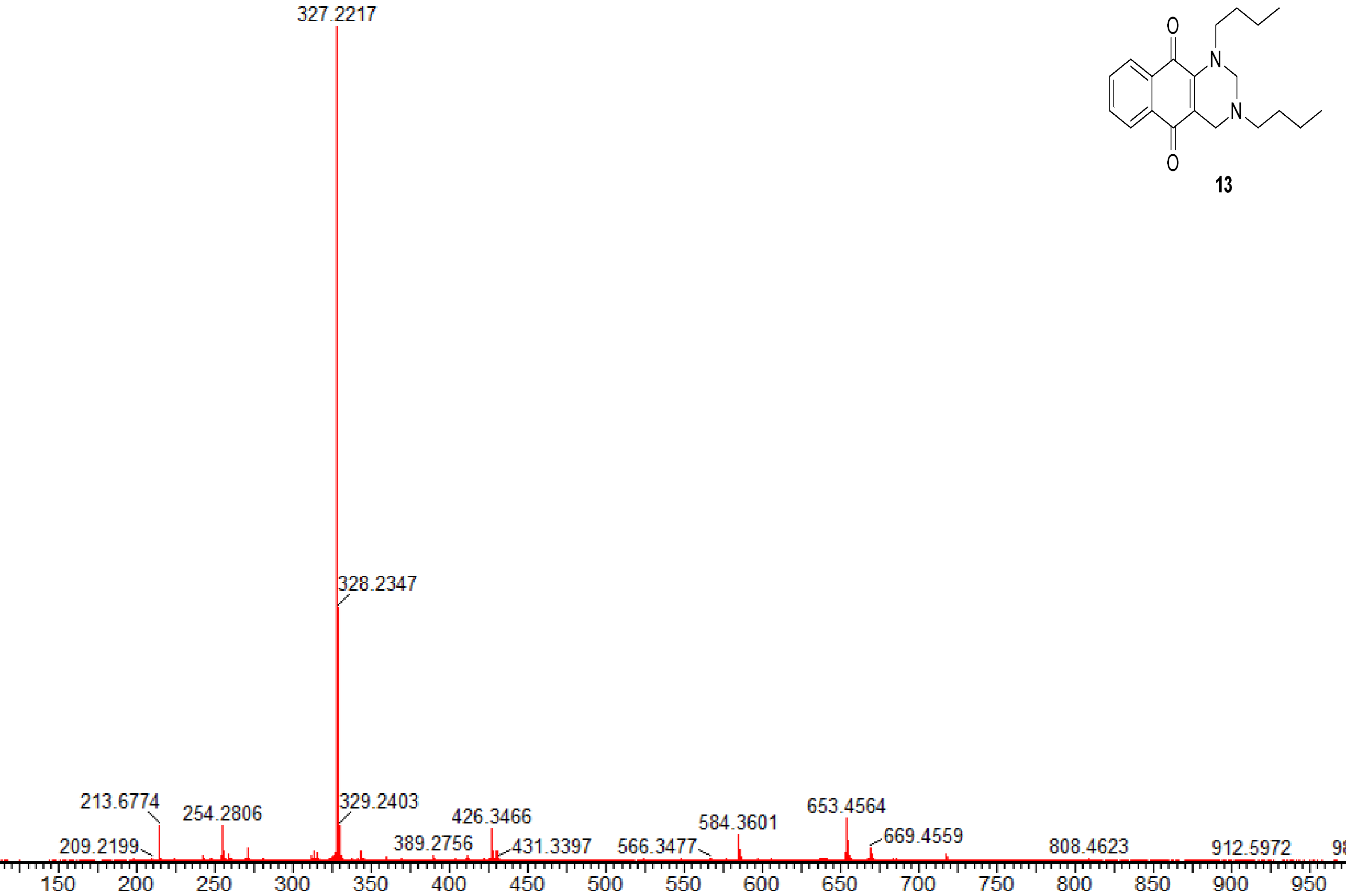

327.2217
328.2347
329.2403
213.6774
254.2806
209.2199
389.2756
426.3466
431.3397
566.3477
584.3601
653.4564
669.4559
808.4623
912.5972
150
200
250
300
350
400
450
500
550
600
650
700
750
800
850
900
950

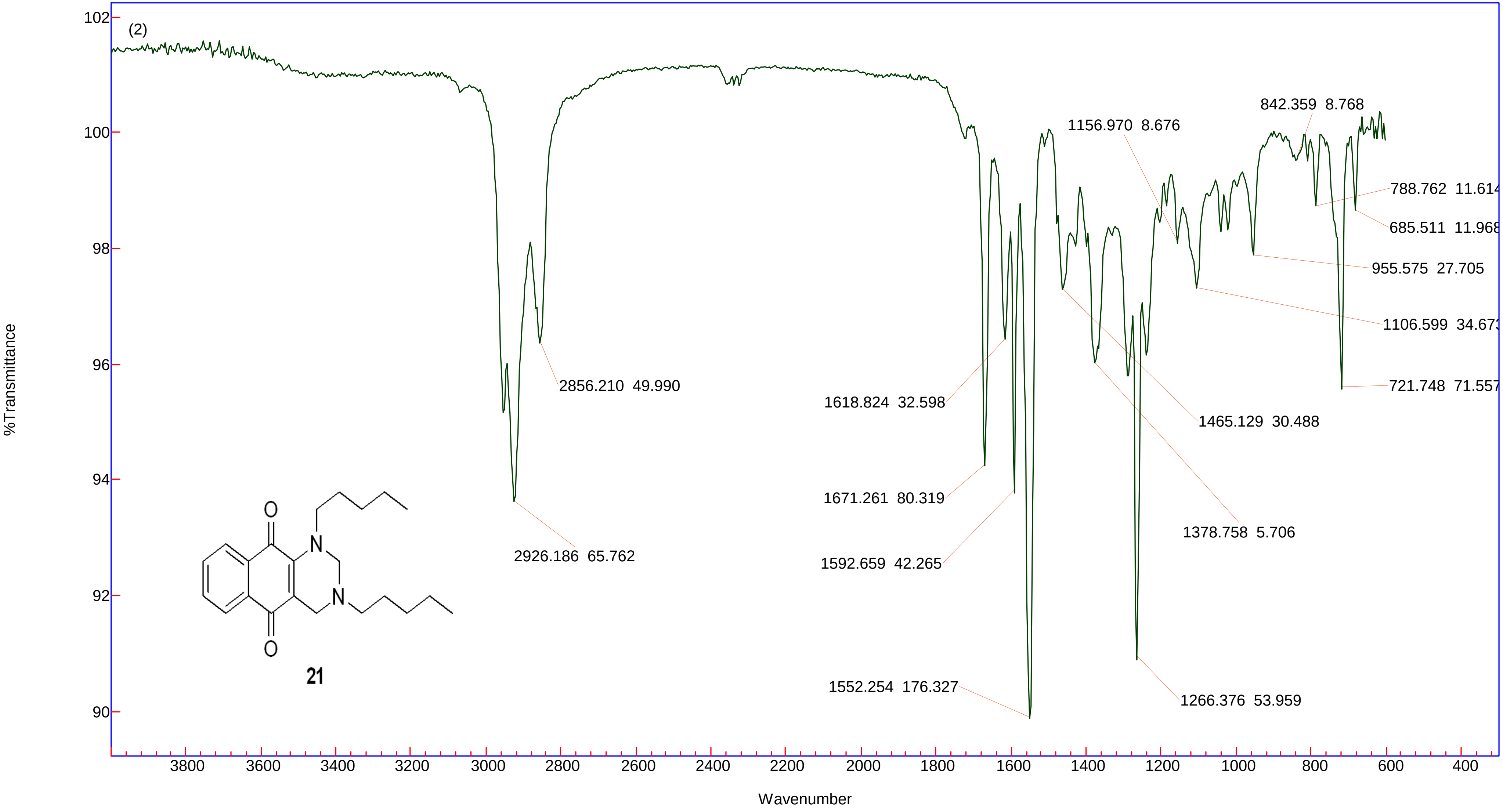
(2)
%Transmittance
102
100
98
96
94
92
90
3800
3600
3400
3200
3000
2800
2600
2400
2200
2000
1800
1600
1400
1200
1000
800
600
400
Wavenumber
2926.186 65.762
2856.210 49.990
1618.824 32.598
1671.261 80.319
1592.659 42.265
1552.254 176.327
1156.970 8.676
842.359 8.768
788.762 11.614
685.511 11.968
955.575 27.705
1106.599 34.673
721.748 71.557
1465.129 30.488
1378.758 5.706
1266.376 53.959

21

Name	
(2)	

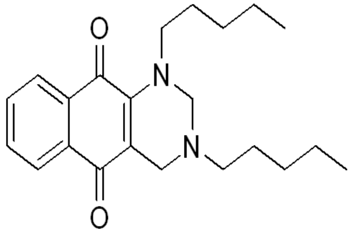

21

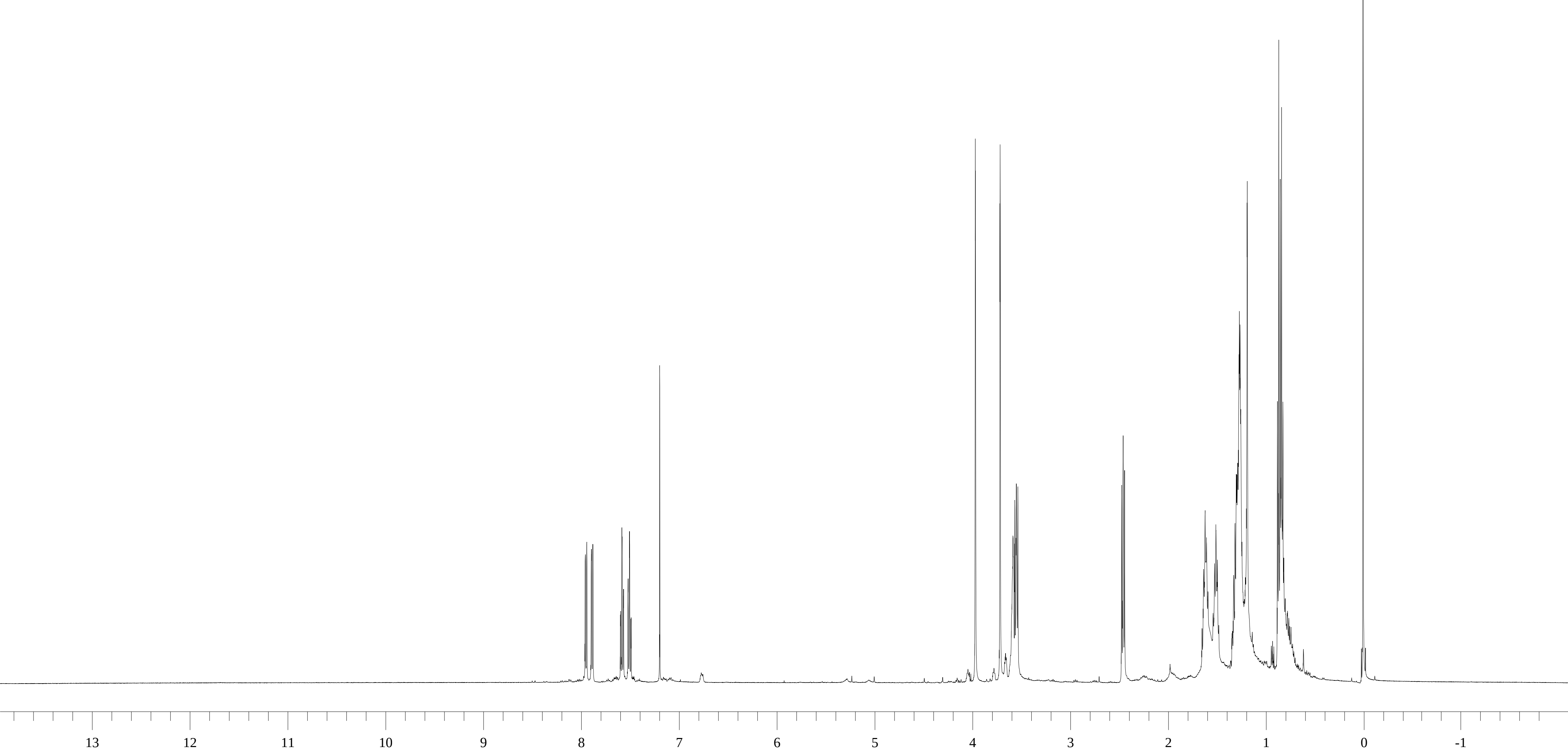

S48

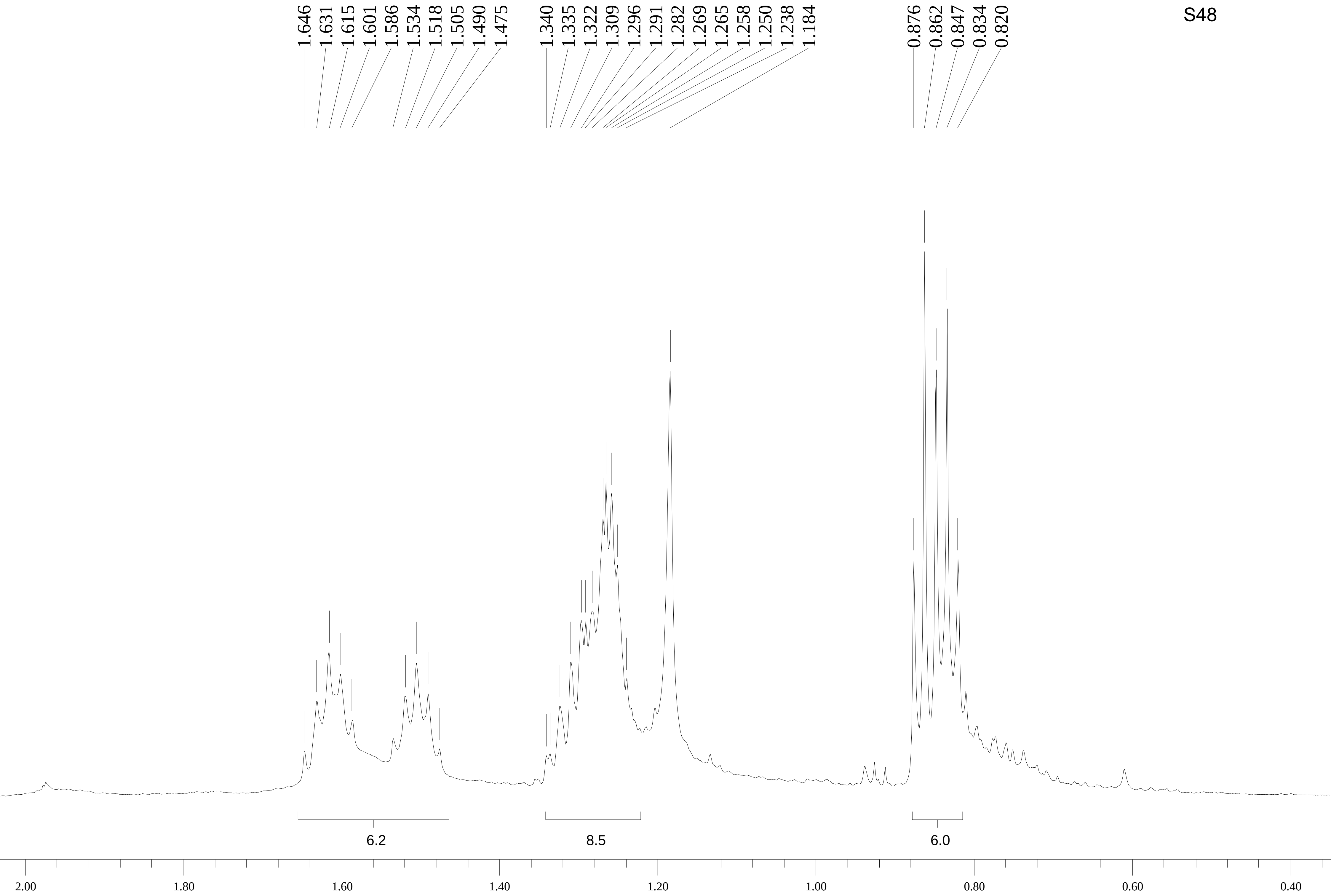

1.646
1.631
1.615
1.601
1.586
1.534
1.518
1.505
1.490
1.475
1.340
1.335
1.322
1.309
1.296
1.291
1.282
1.269
1.265
1.258
1.250
1.238
1.184
0.876
0.862
0.847
0.834
0.820
6.2
8.5
6.0
2.00
1.80
1.60
1.40
1.20
1.00
0.80
0.60
0.40

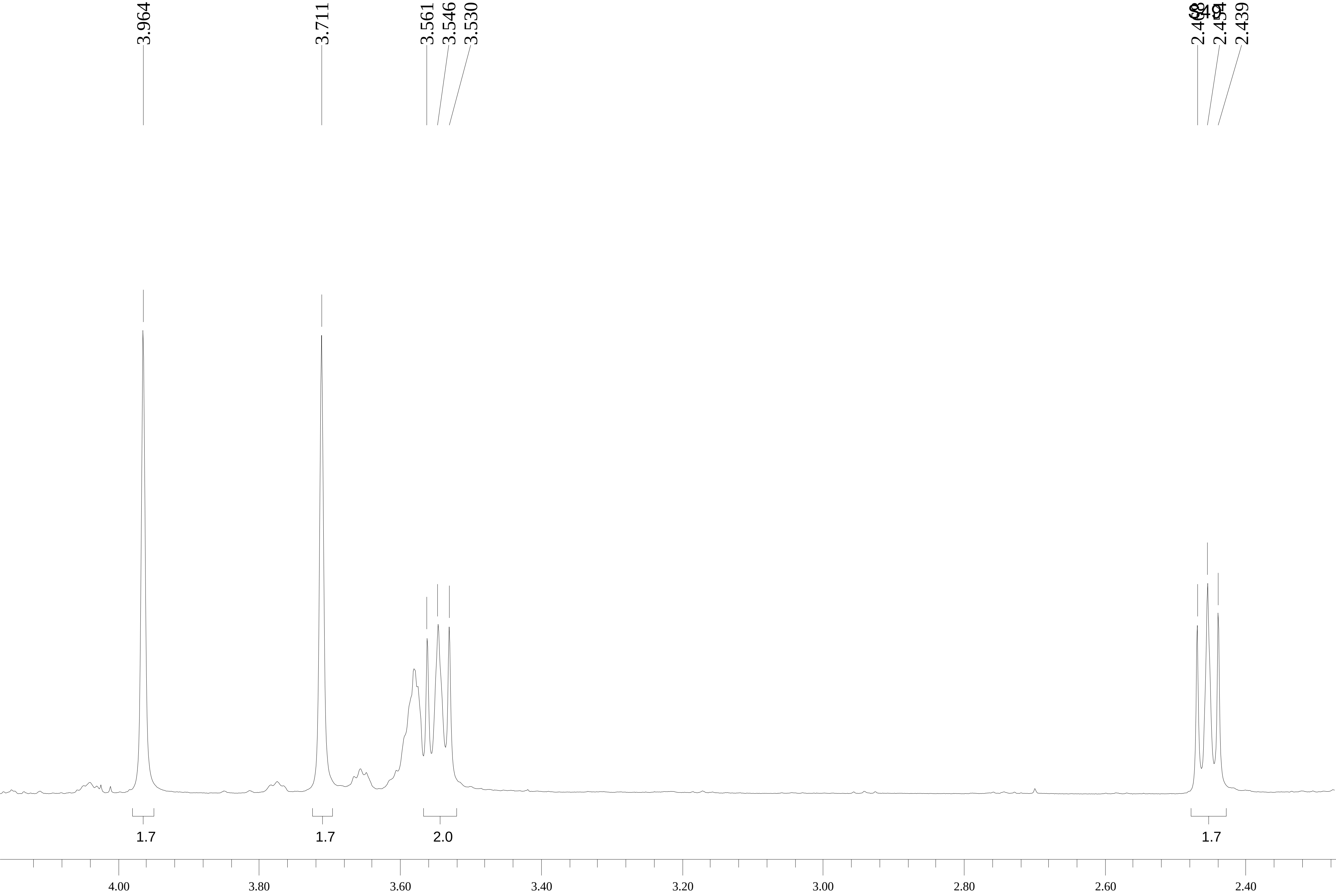
3.964
3.711
3.561
3.546
3.530
2.454
2.439
1.7
1.7
2.0
1.7
4.00
3.80
3.60
3.40
3.20
3.00
2.80
2.60
2.40

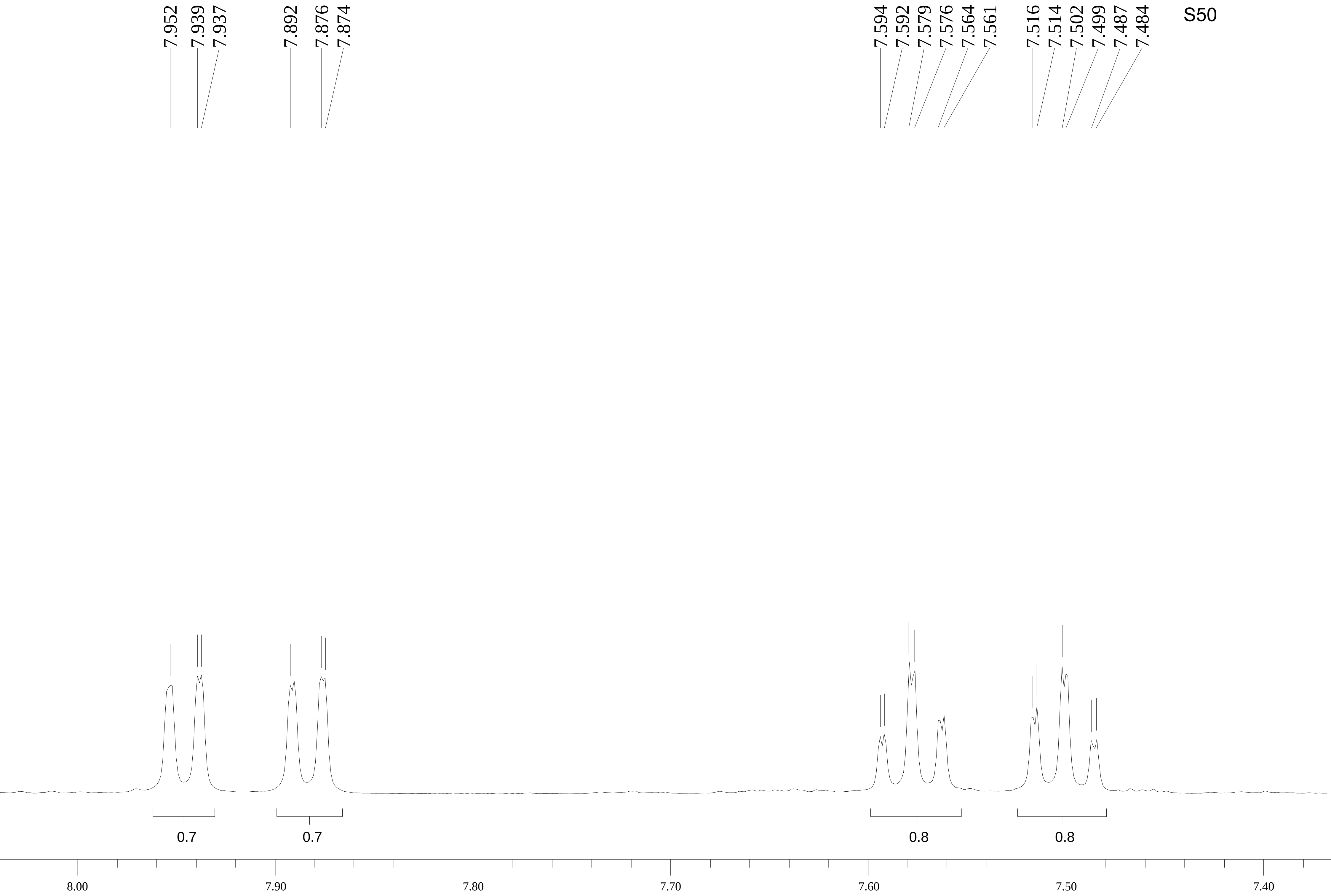
7.952
7.939
7.937
7.892
7.876
7.874
7.594
7.592
7.579
7.576
7.564
7.561
7.516
7.514
7.502
7.499
7.487
7.484
0.7
0.7
0.8
0.8
8.00
7.90
7.80
7.70
7.60
7.50
7.40

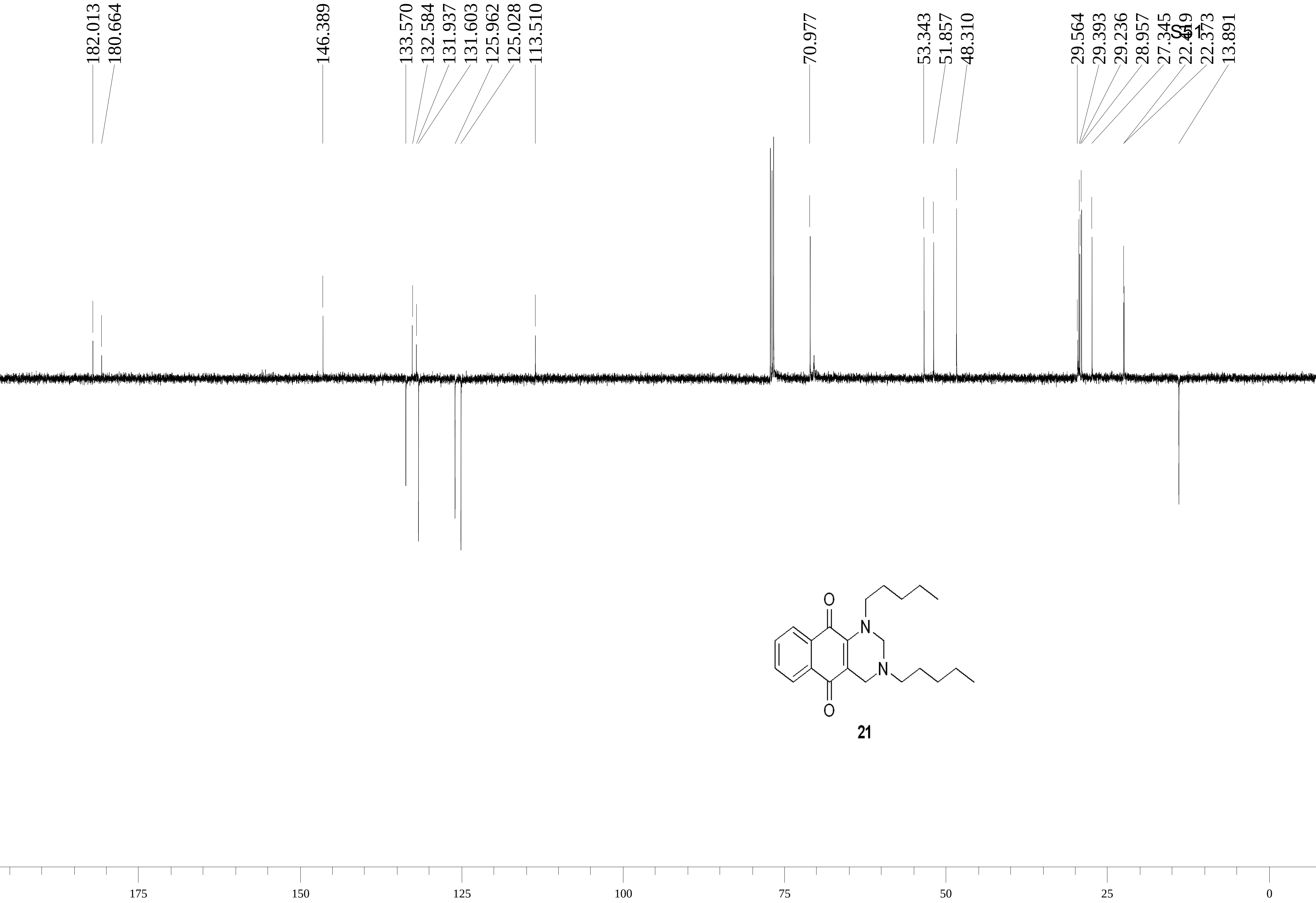
182.013
180.664
146.389
133.570
132.584
131.937
131.603
125.962
125.028
113.510
70.977
53.343
51.857
48.310
29.564
29.393
29.236
28.957
27.345
22.419
22.373
13.891
O
N
N
O
21
175
150
125
100
75
50
25
0

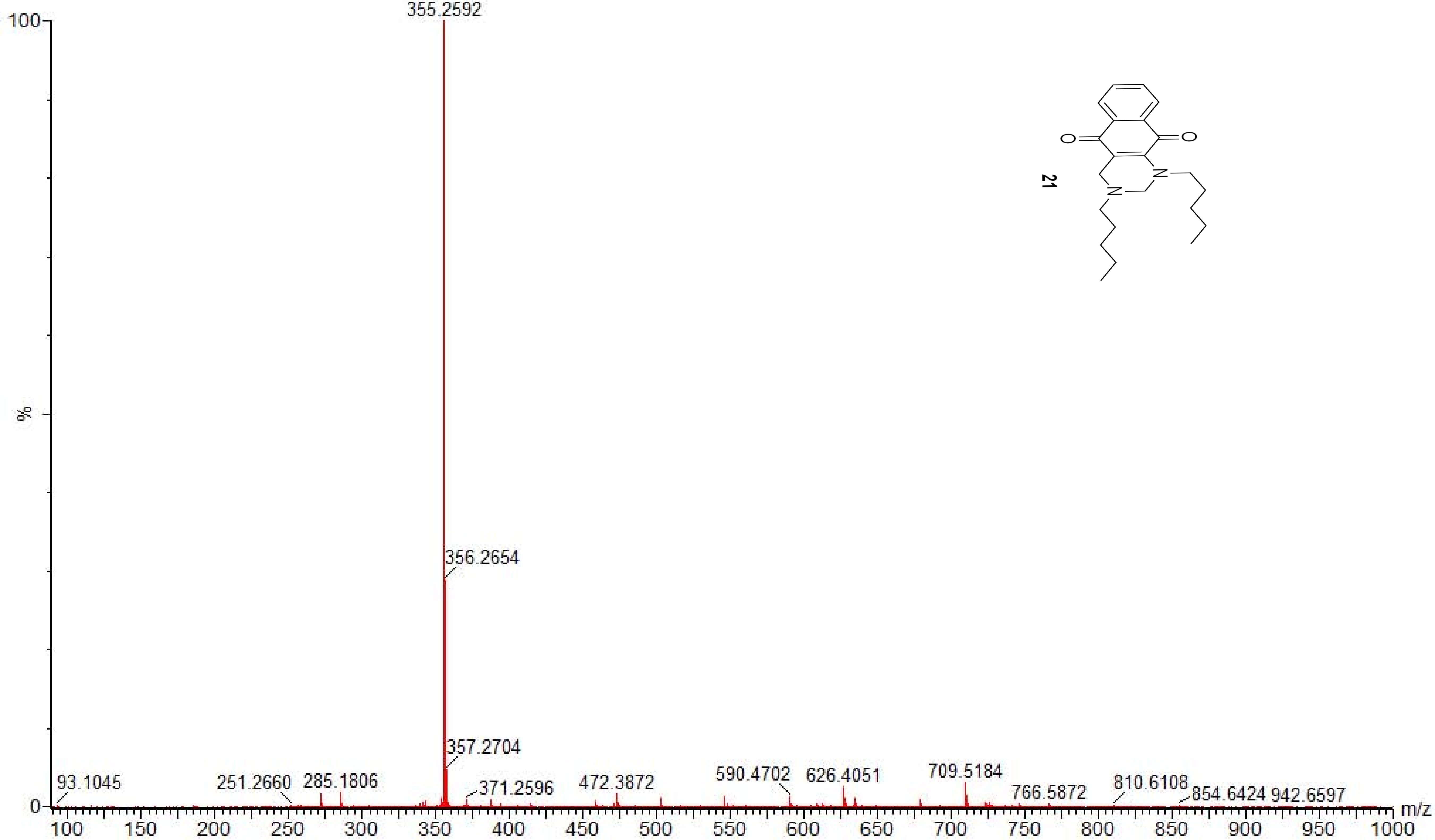
355.2592
356.2654
357.2704
93.1045
251.2660
285.1806
371.2596
472.3872
590.4702
626.4051
709.5184
766.5872
810.6108
854.6424
942.6597
100
%
0
m/z
100 150 200 250 300 350 400 450 500 550 600 650 700 750 800 850 900 950 1000
O
O
N
N

21

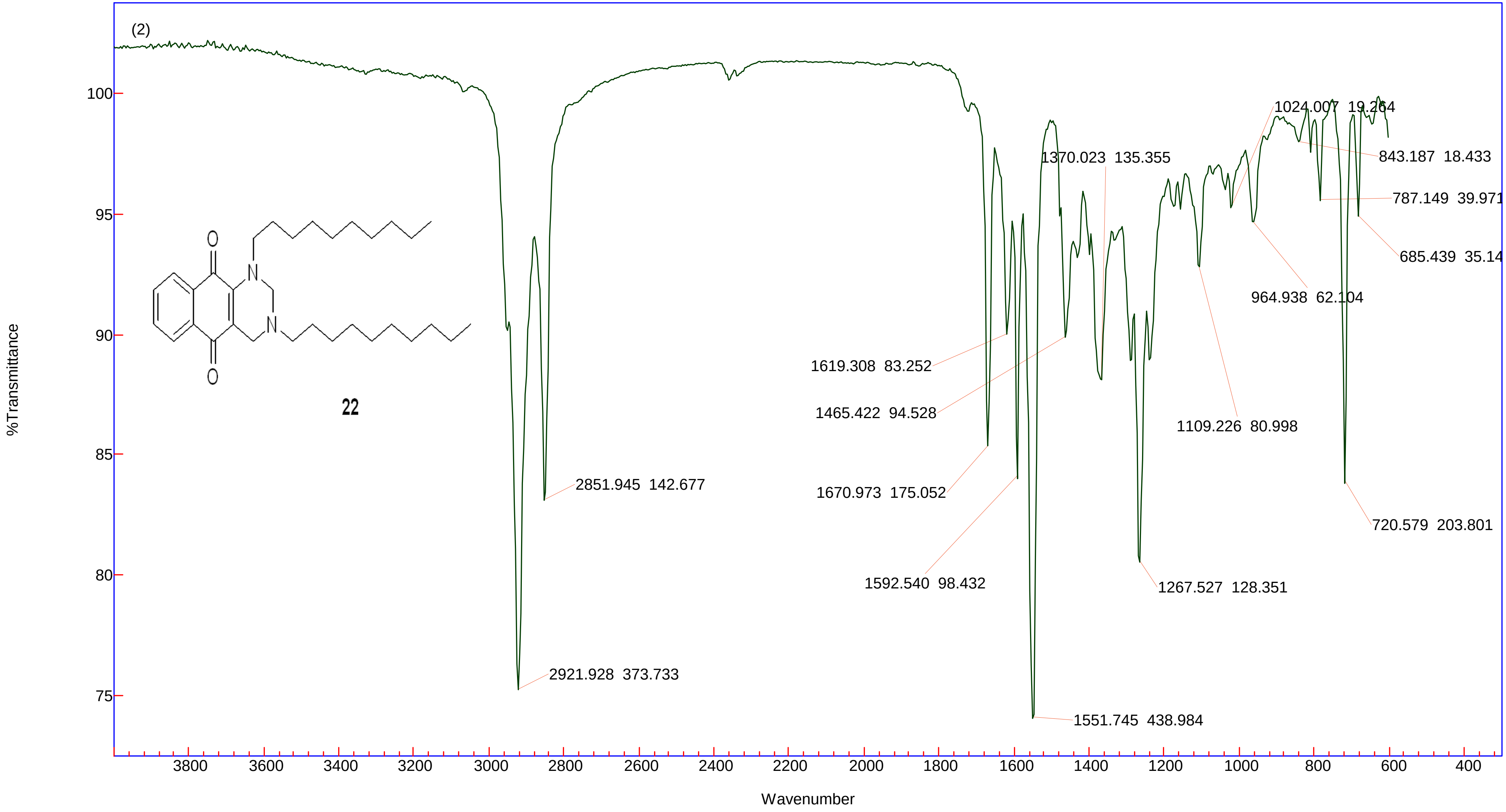
(2)
%Transmittance
100
95
90
85
80
75
O
N
N
O
22
2851.945 142.677
2921.928 373.733
1619.308 83.252
1465.422 94.528
1670.973 175.052
1592.540 98.432
1551.745 438.984
1370.023 135.355
1267.527 128.351
1109.226 80.998
1024.007 19.264
964.938 62.104
843.187 18.433
787.149 39.971
685.439 35.14
720.579 203.801
3800
3600
3400
3200
3000
2800
2600
2400
2200
2000
1800
1600
1400
1200
1000
800
600
400
Wavenumber

Name	
(2)	———

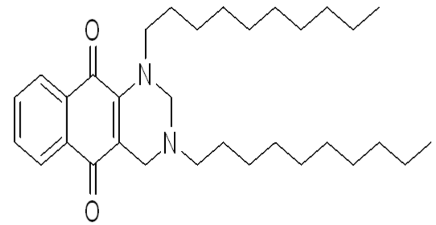

22

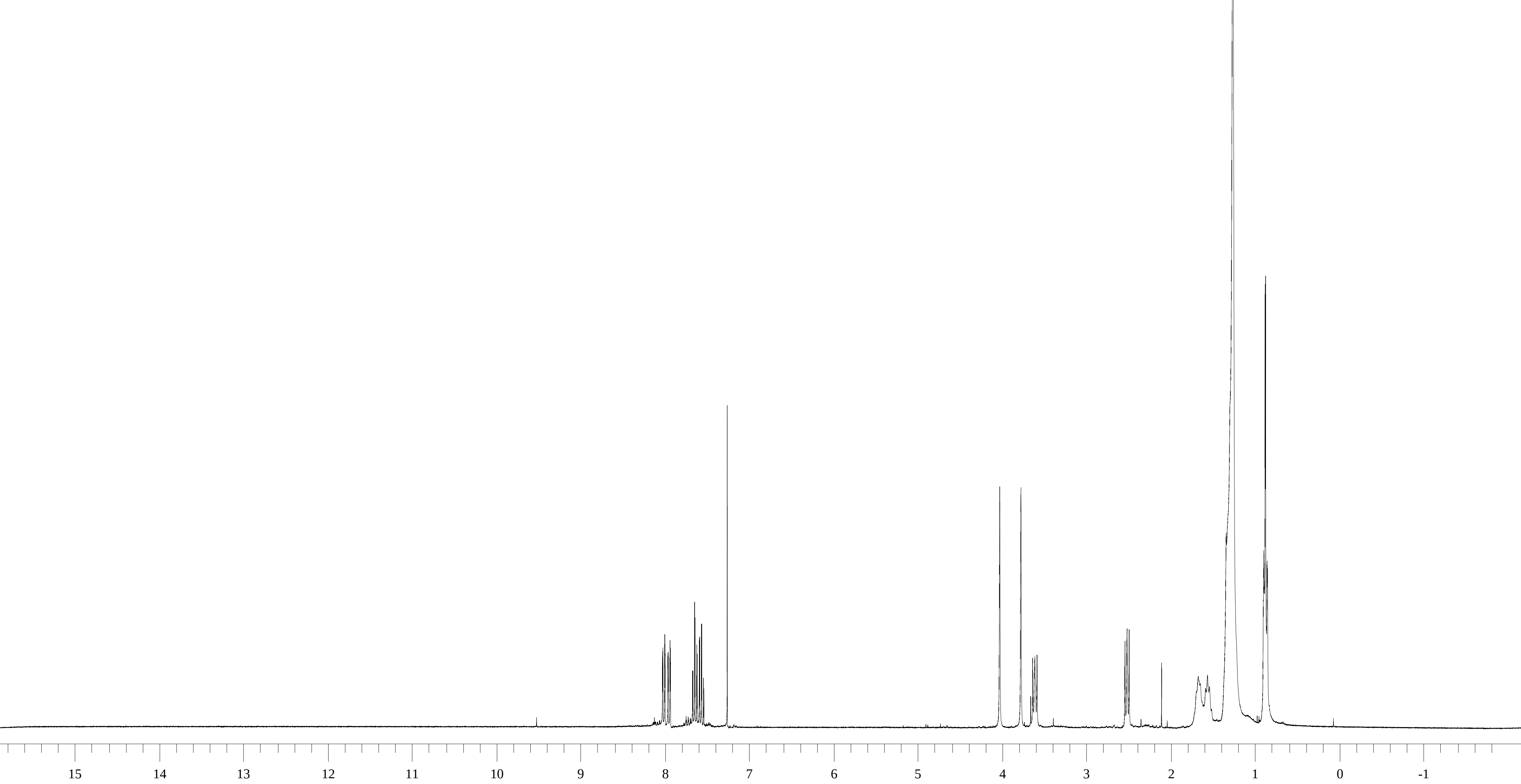

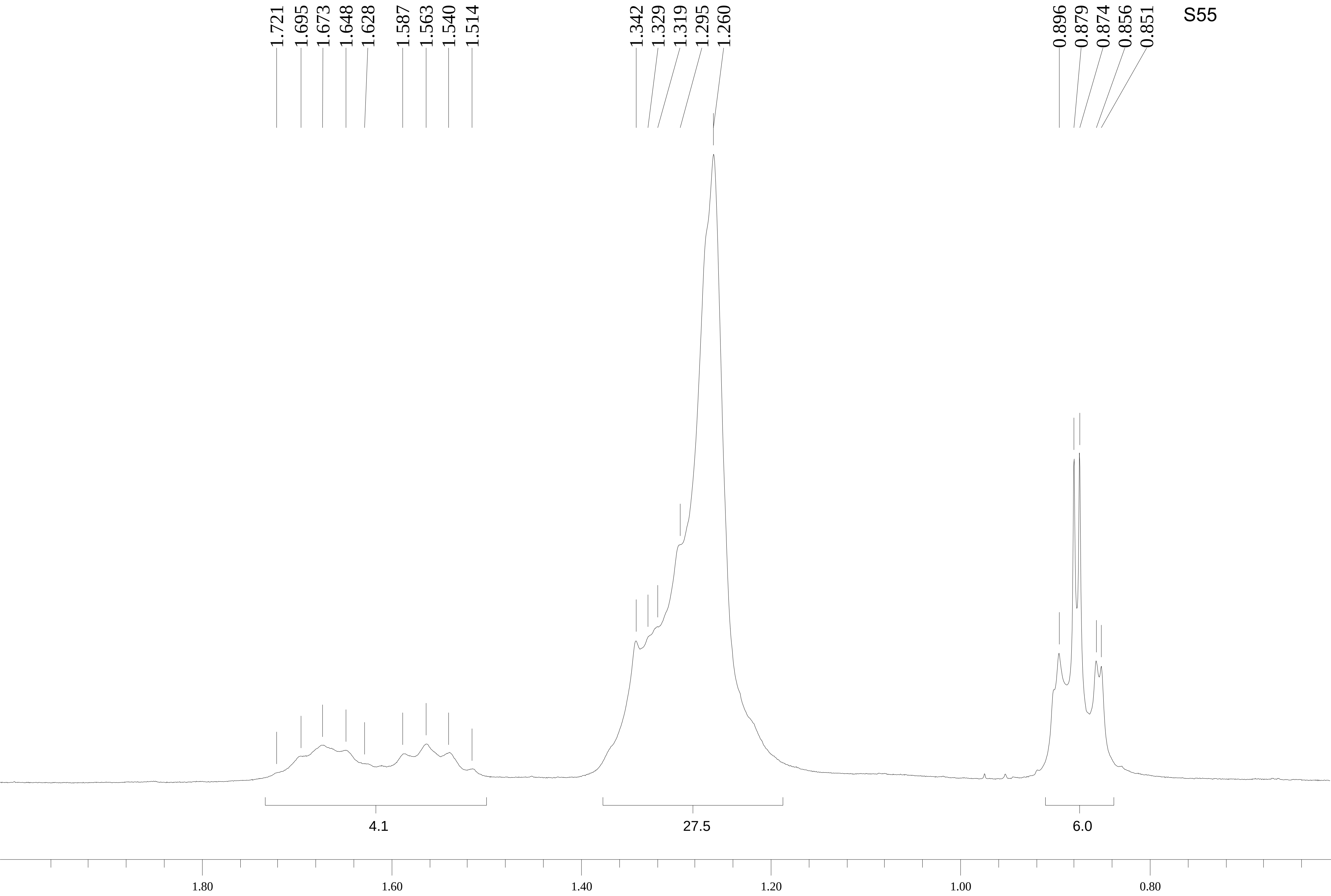
S55
1.721
1.695
1.673
1.648
1.628
1.587
1.563
1.540
1.514
1.342
1.329
1.319
1.295
1.260
0.896
0.879
0.874
0.856
0.851
4.1
27.5
6.0
1.80
1.60
1.40
1.20
1.00
0.80

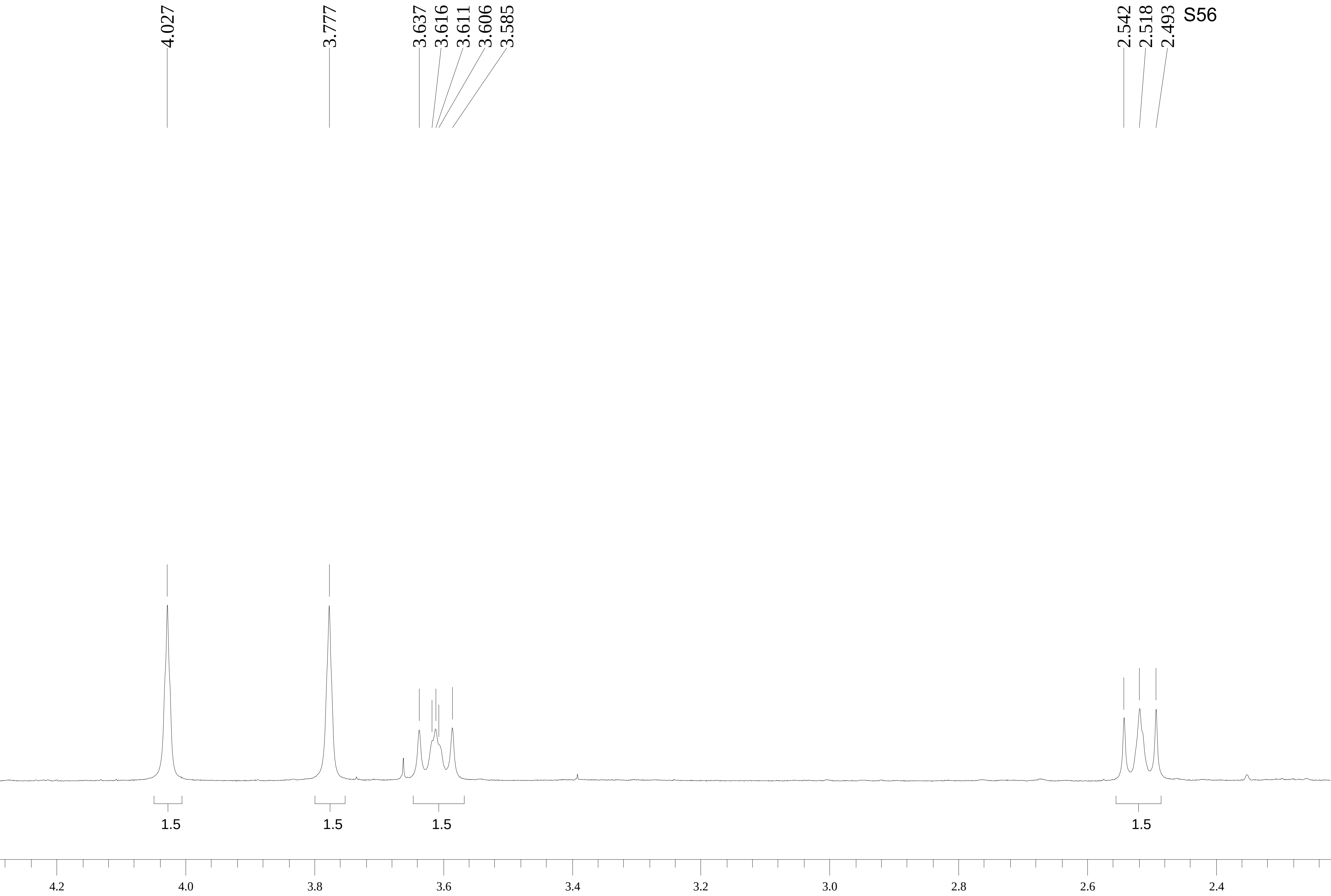
4.027
3.777
3.637
3.616
3.611
3.606
3.585
2.542
2.518
2.493
1.5
1.5
1.5
1.5
4.2
4.0
3.8
3.6
3.4
3.2
3.0
2.8
2.6
2.4

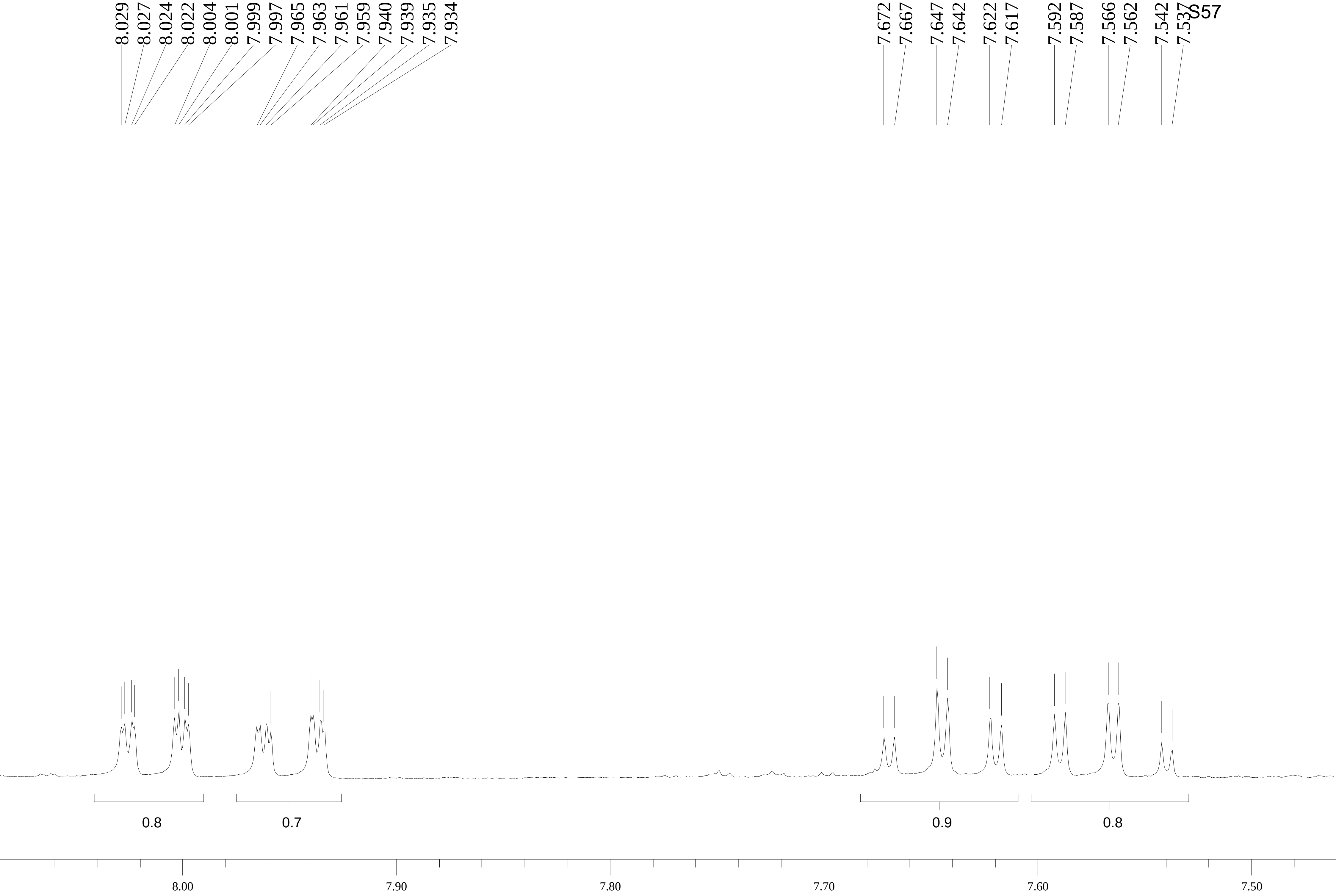

8.029
8.027
8.024
8.022
8.004
8.001
7.999
7.997
7.965
7.963
7.961
7.959
7.940
7.939
7.935
7.934
7.672
7.667
7.647
7.642
7.622
7.617
7.592
7.587
7.566
7.562
7.542
7.537
0.8
0.7
0.9
0.8
8.00
7.90
7.80
7.70
7.60
7.50

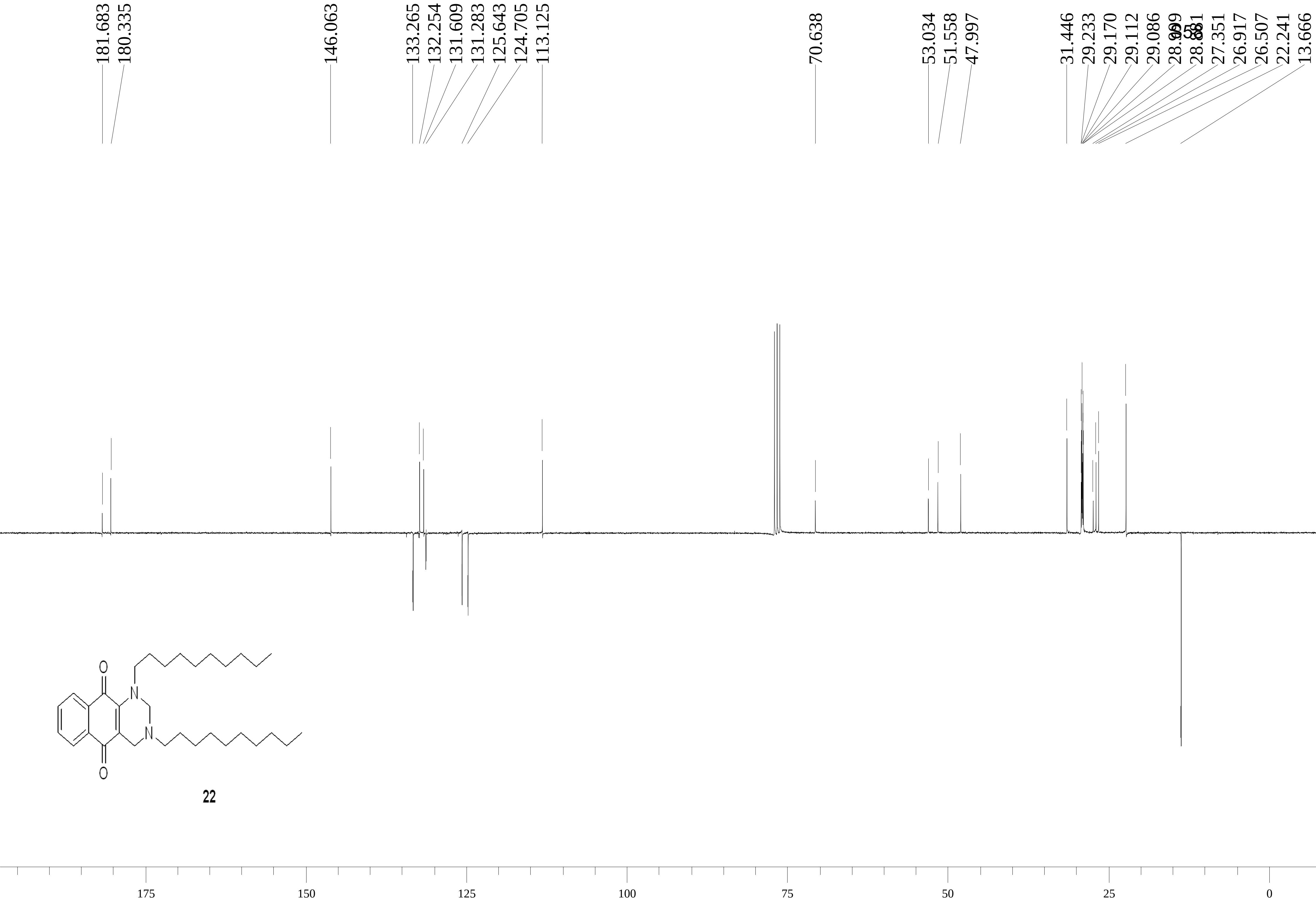
181.683
180.335
146.063
133.265
132.254
131.609
131.283
125.643
124.705
113.125
70.638
53.034
51.558
47.997
31.446
29.233
29.170
29.112
29.086
27.351
26.917
26.507
22.241
13.666
175
150
125
100
75
50
25
0
22

HELVECIO

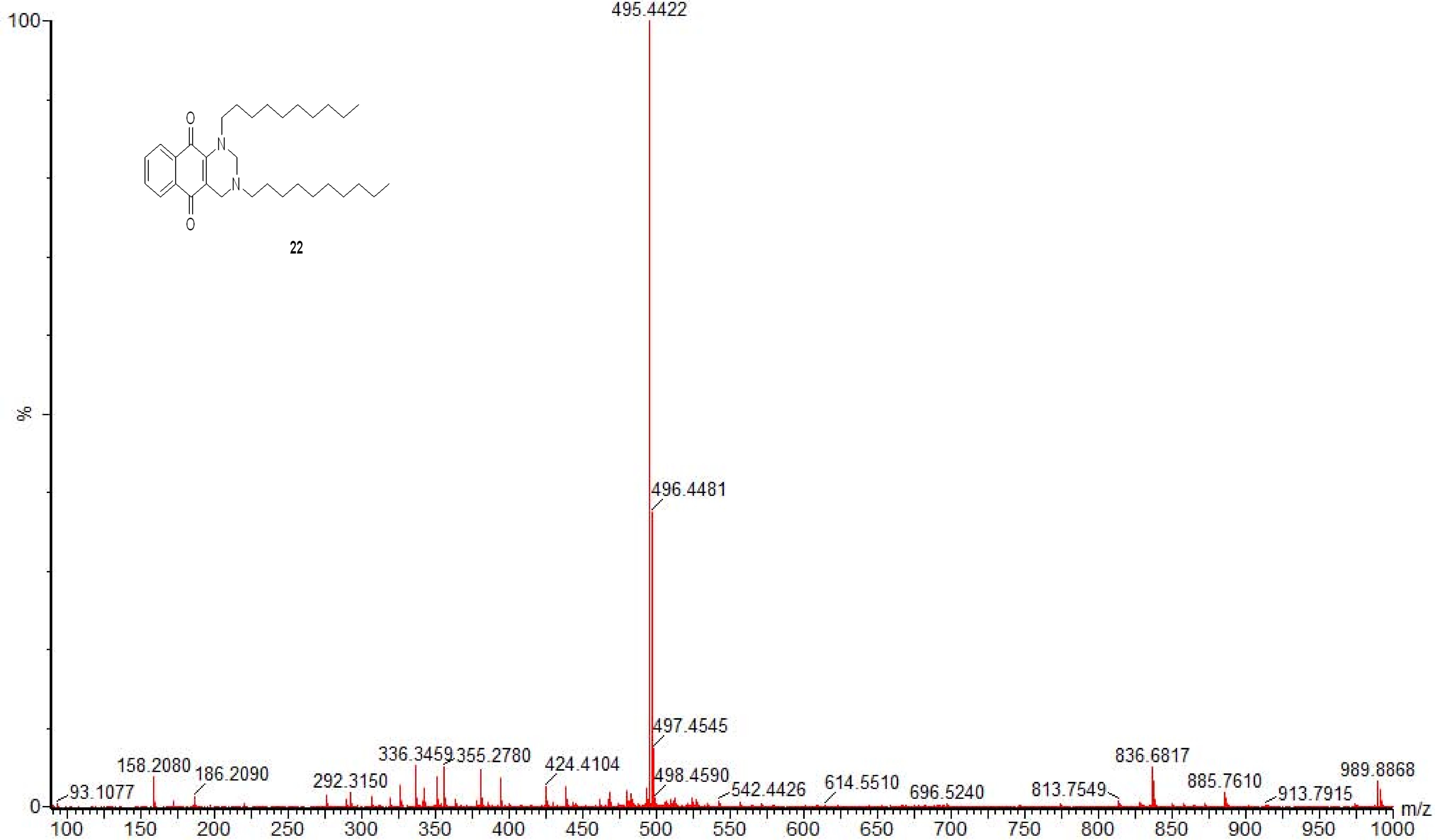

495.4422
100
%
0
496.4481
497.4545
498.4590
158.2080
93.1077
186.2090
292.3150
336.3459
355.2780
424.4104
542.4426
614.5510
696.5240
813.7549
836.6817
885.7610
913.7915
989.8868
m/z
100
150
200
250
300
350
400
450
500
550
600
650
700
750
800
850
900
950
1000
O
N
N
O
22

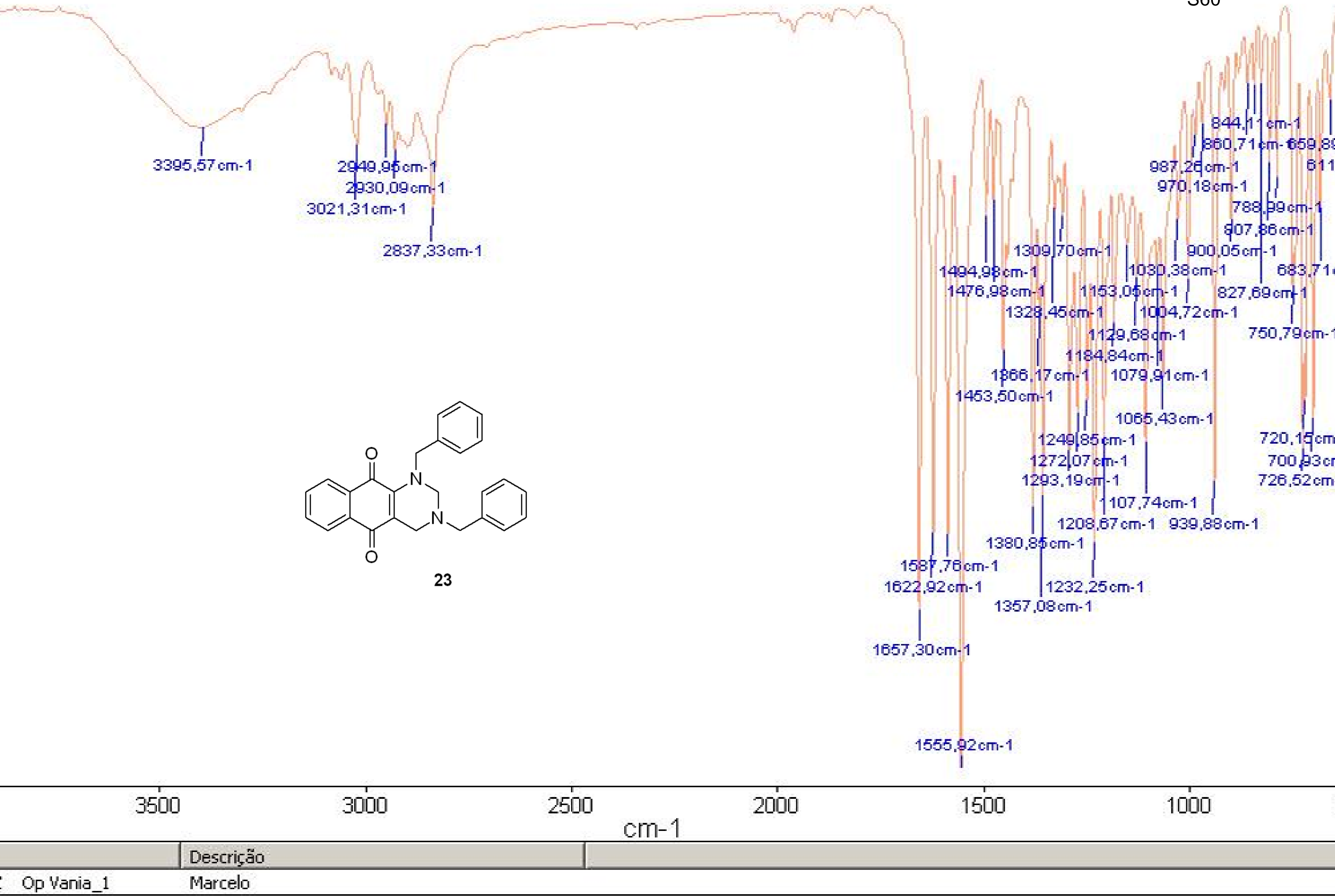
3395,57cm-1
2949,95cm-1
2930,09cm-1
3021,31cm-1
2837,33cm-1
1494,98cm-1
1476,98cm-1
1453,50cm-1
1366,17cm-1
1328,45cm-1
1309,70cm-1
1380,85cm-1
1357,08cm-1
1293,19cm-1
1272,07cm-1
1249,85cm-1
1232,25cm-1
1208,67cm-1
1184,84cm-1
1153,05cm-1
1129,68cm-1
1107,74cm-1
1079,91cm-1
1065,43cm-1
1030,38cm-1
1004,72cm-1
987,26cm-1
970,18cm-1
939,88cm-1
900,05cm-1
860,71cm-1
844,11cm-1
827,69cm-1
807,86cm-1
788,99cm-1
750,79cm-1
1657,30cm-1
1622,92cm-1
1587,76cm-1
1555,92cm-1
3500
3000
2500
2000
1500
1000
cm-1
Descrição
Op Vania_1
Marcelo

23

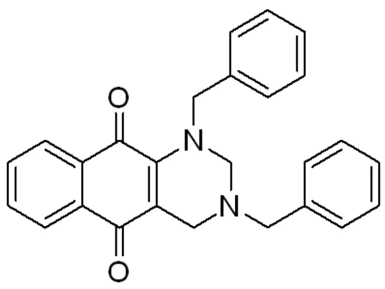

23

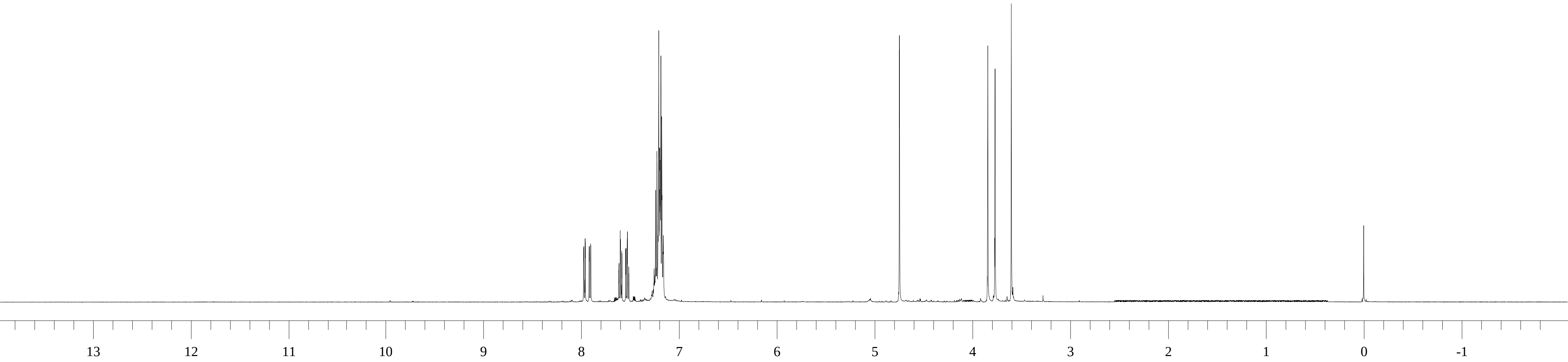

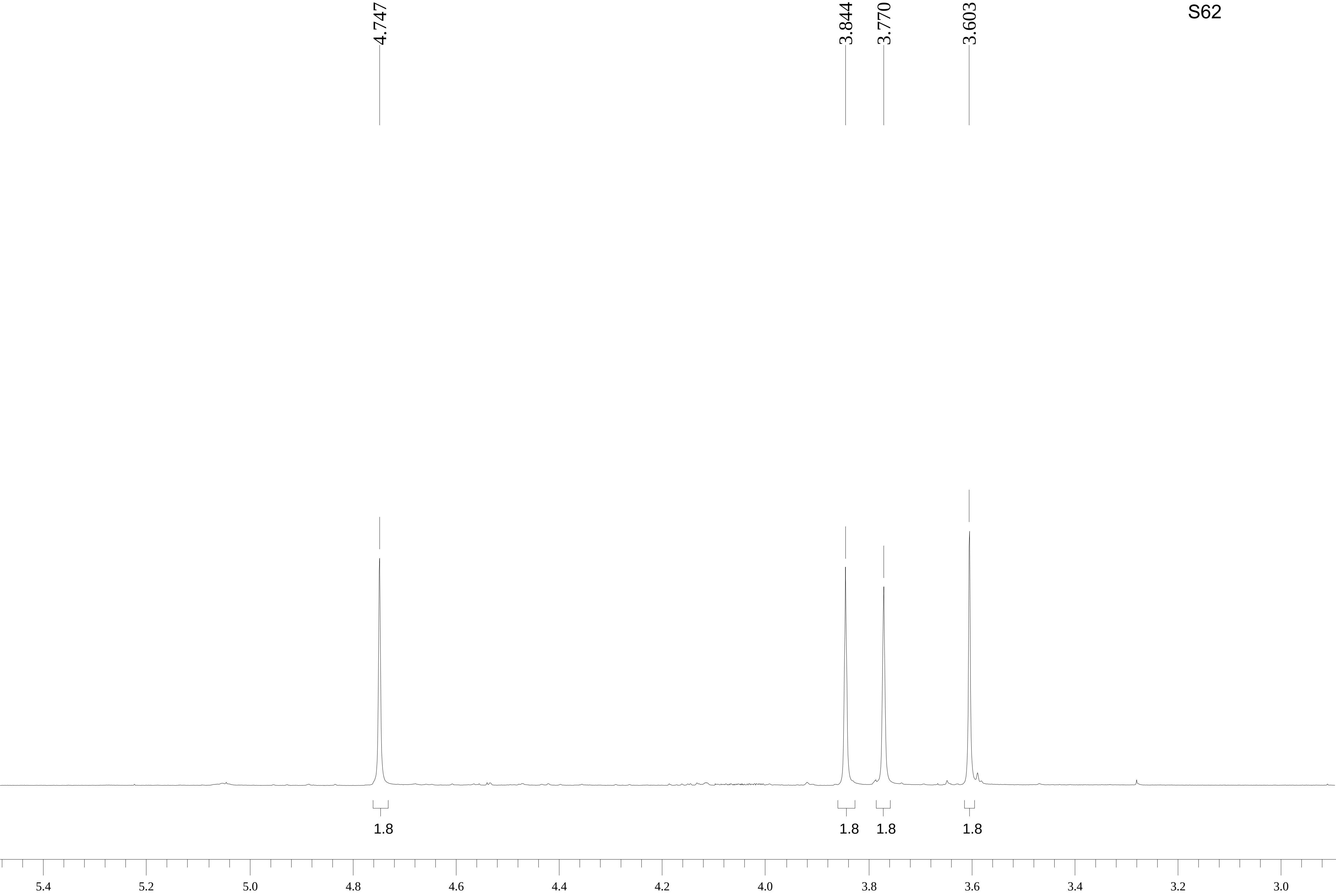

4.747
3.844
3.770
3.603
1.8
1.8
1.8
1.8
5.4
5.2
5.0
4.8
4.6
4.4
4.2
4.0
3.8
3.6
3.4
3.2
3.0

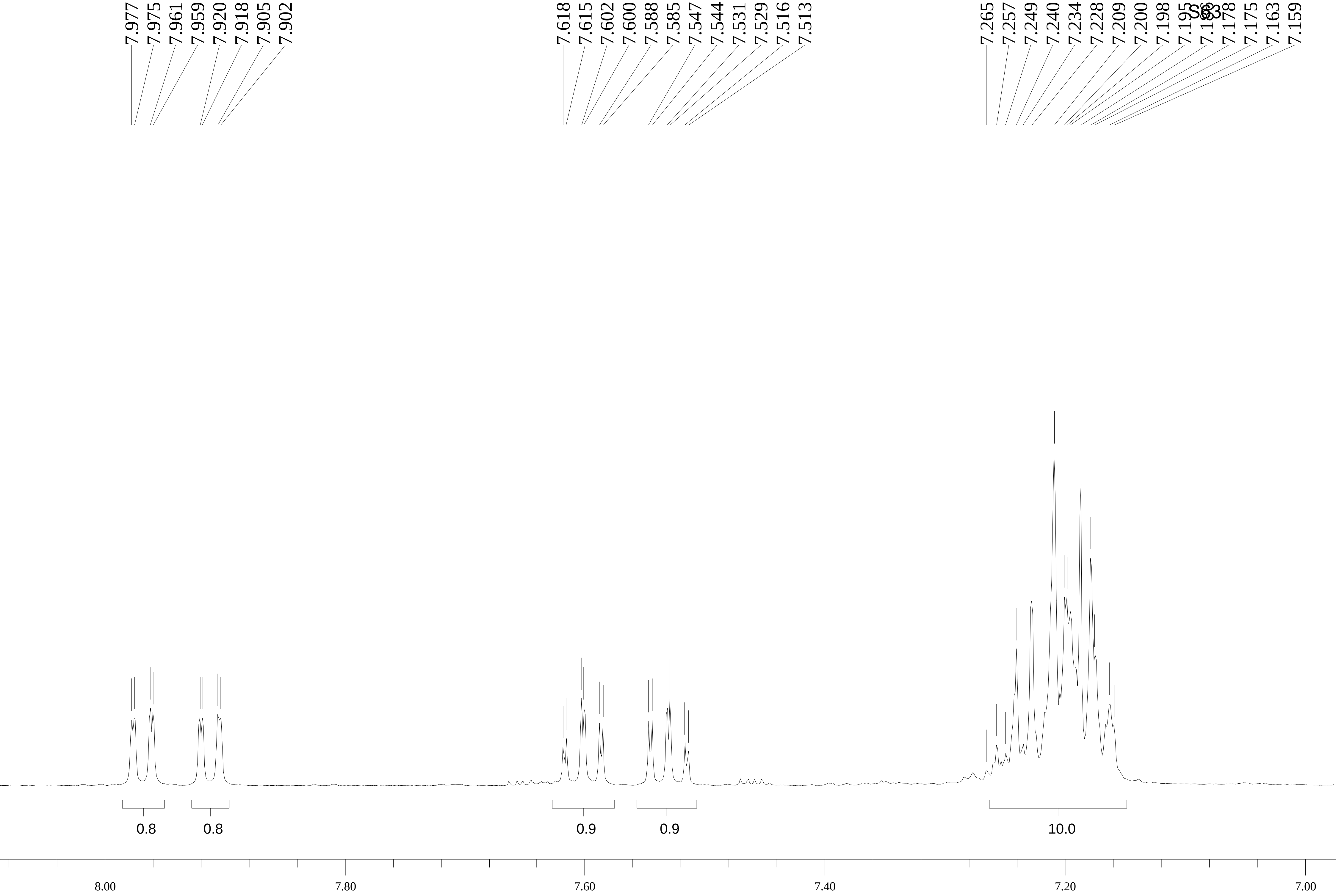
7.977
7.975
7.961
7.959
7.920
7.918
7.905
7.902
7.618
7.615
7.602
7.600
7.588
7.585
7.547
7.544
7.531
7.529
7.516
7.513
7.265
7.257
7.249
7.240
7.234
7.228
7.209
7.200
7.198
7.195
7.178
7.175
7.163
7.159
0.8
0.8
0.9
0.9
10.0
8.00
7.80
7.60
7.40
7.20
7.00

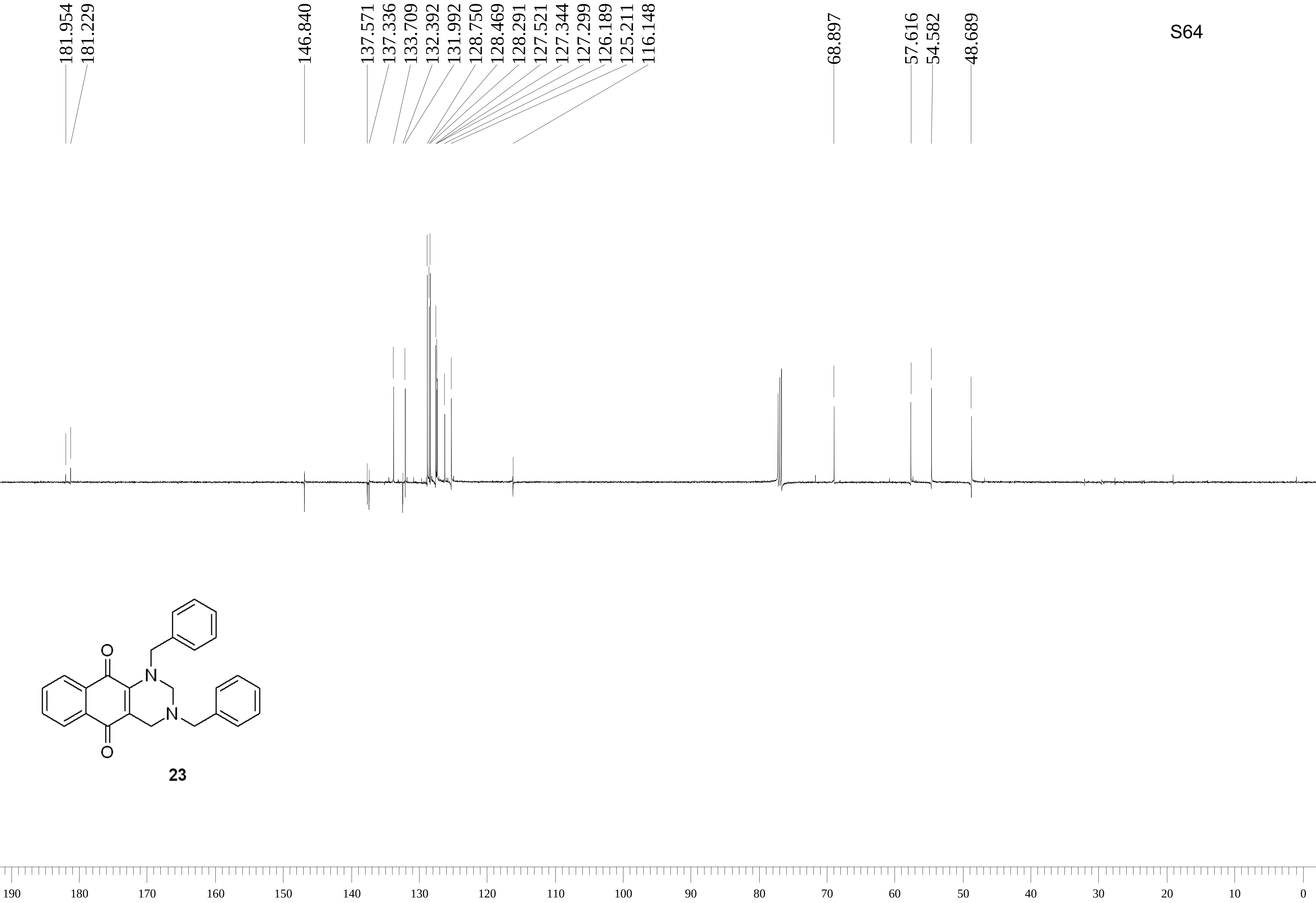
181.954
181.229
146.840
137.571
137.336
133.709
132.392
131.992
128.750
128.469
128.291
127.521
127.344
127.299
126.189
125.211
116.148
68.897
57.616
54.582
48.689
O
N
N
O
23
190 180 170 160 150 140 130 120 110 100 90 80 70 60 50 40 30 20 10 0

CIO

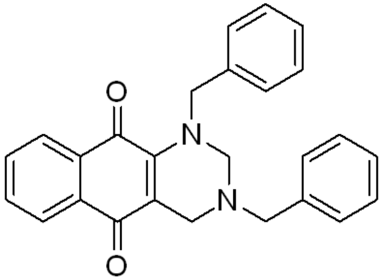

23

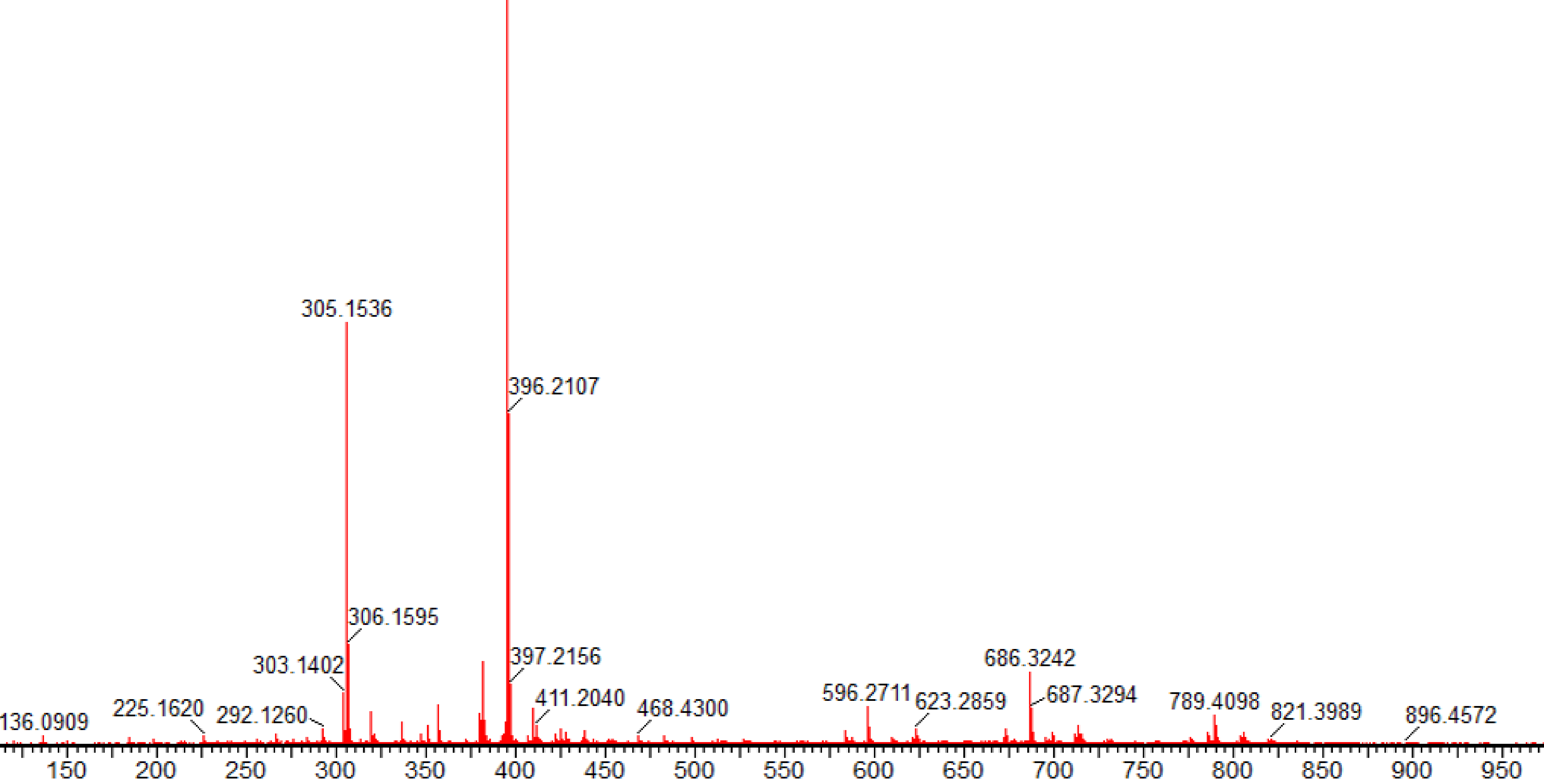
395.2062
305.1536
396.2107
306.1595
303.1402
397.2156
686.3242
596.2711
623.2859
687.3294
411.2040
468.4300
789.4098
821.3989
896.4572
136.0909
225.1620
292.1260
150
200
250
300
350
400
450
500
550
600
650
700
750
800
850
900
950

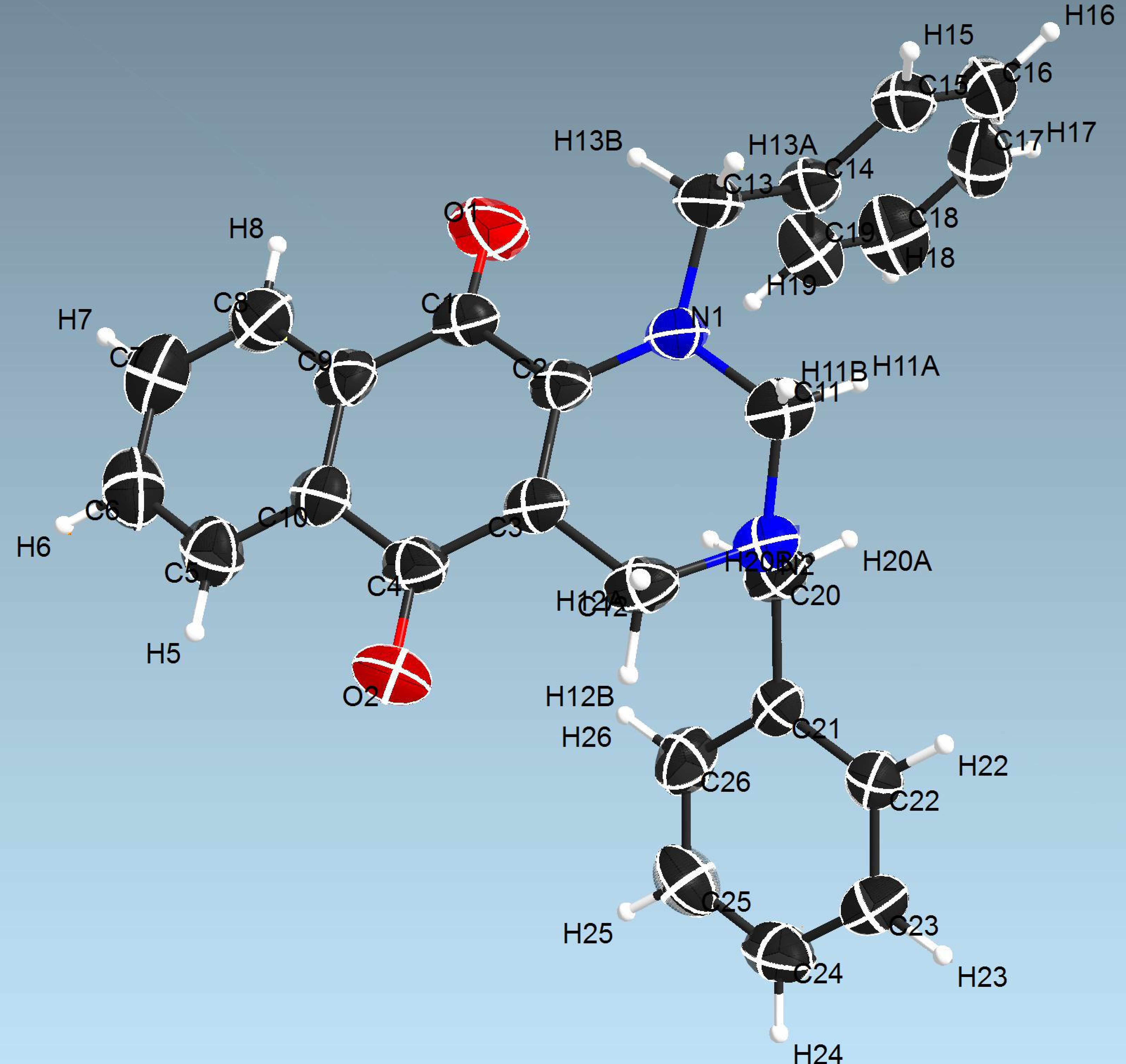

H16
H15
C15
C16
H13B
H13A
C17
H17
C13
C14
O1
C18
H8
C19
H18
H19
C8
C1
N1
H7
C7
C9
C2
H11B
H11A
C11
C6
C10
C3
H6
H20A
C5
C4
C20
H5
O2
H12B
H26
C21
H22
C26
C22
C25
C23
H25
C24
H23
H24

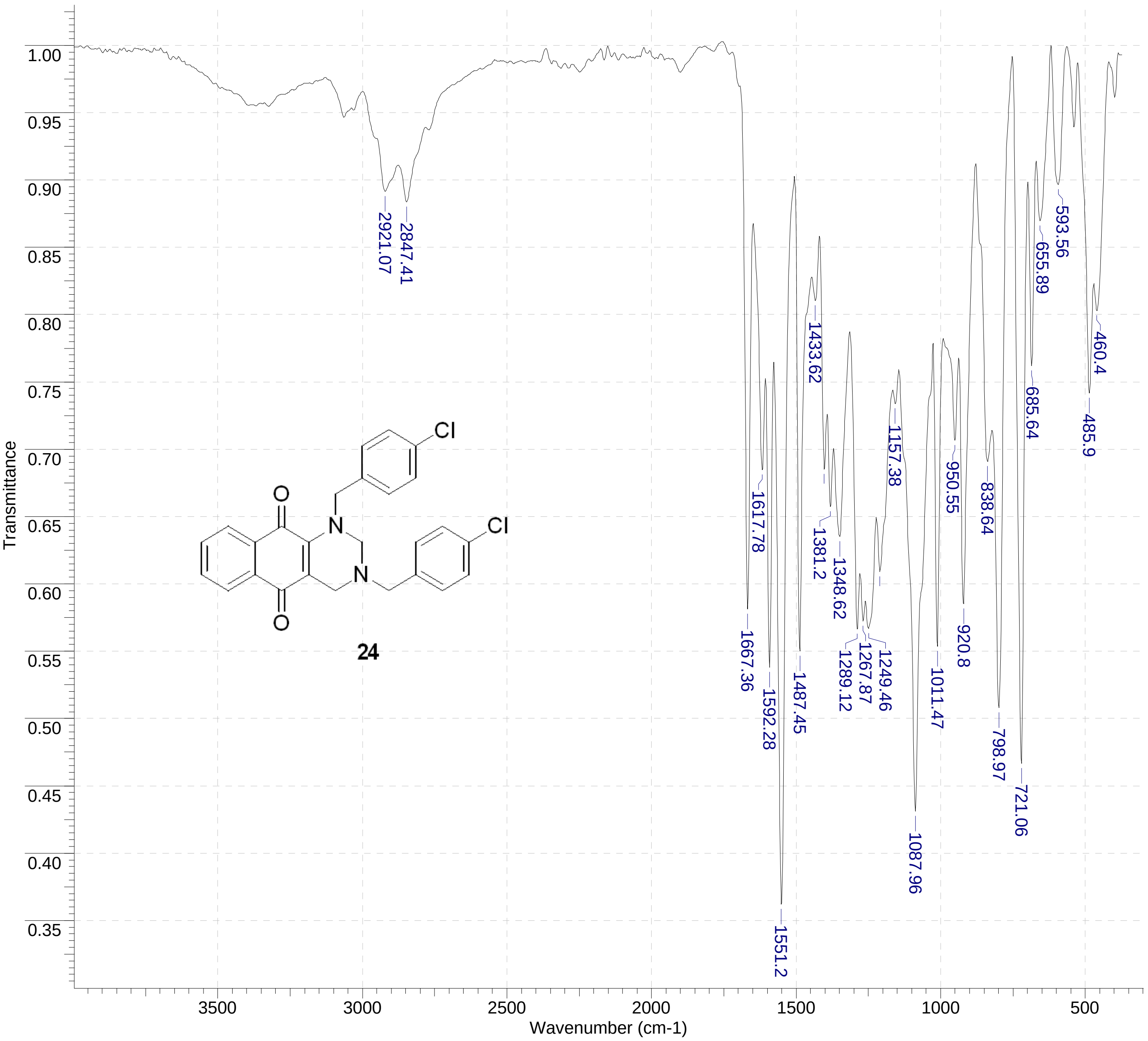

No	cm-1	T	FWHH	Asym	Intensity	No	cm-1	T	FWHH	Asym	Intensity
1	460.40	0.803	-	-	M	15	1249.46	0.567	-	-	S
2	485.90	0.742	-	-	M	16	1267.87	0.572	-	-	S
3	593.56	0.897	-1.00	0.00	W	17	1289.12	0.567	-	-	S
4	655.89	0.870	-	-	W	18	1348.62	0.635	-	-	M
5	685.64	0.762	-1.00	0.00	M	19	1381.20	0.657	-	-	M
6	721.06	0.467	-1.00	0.00	S	20	1402.45	0.685	-	-	M
7	798.97	0.508	-1.00	0.00	S	21	1433.62	0.810	-	-	M
8	838.64	0.691	-	-	M	22	1487.45	0.550	-1.00	0.00	S
9	920.80	0.585	-1.00	0.00	S	23	1551.20	0.362	-1.00	0.00	VS
10	950.55	0.706	-	-	M	24	1592.28	0.538	-1.00	0.00	S
11	1011.47	0.554	-1.00	0.00	S	25	1617.78	0.685	-	-	M
12	1087.96	0.431	-1.00	0.00	S	26	1667.36	0.581	-1.00	0.00	S
13	1157.38	0.734	-	-	M	27	2847.41	0.884	-	-	W
14	1209.79	0.609	-	-	S	28	2921.07	0.892	-	-	W

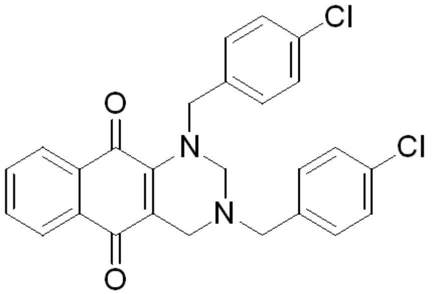

24

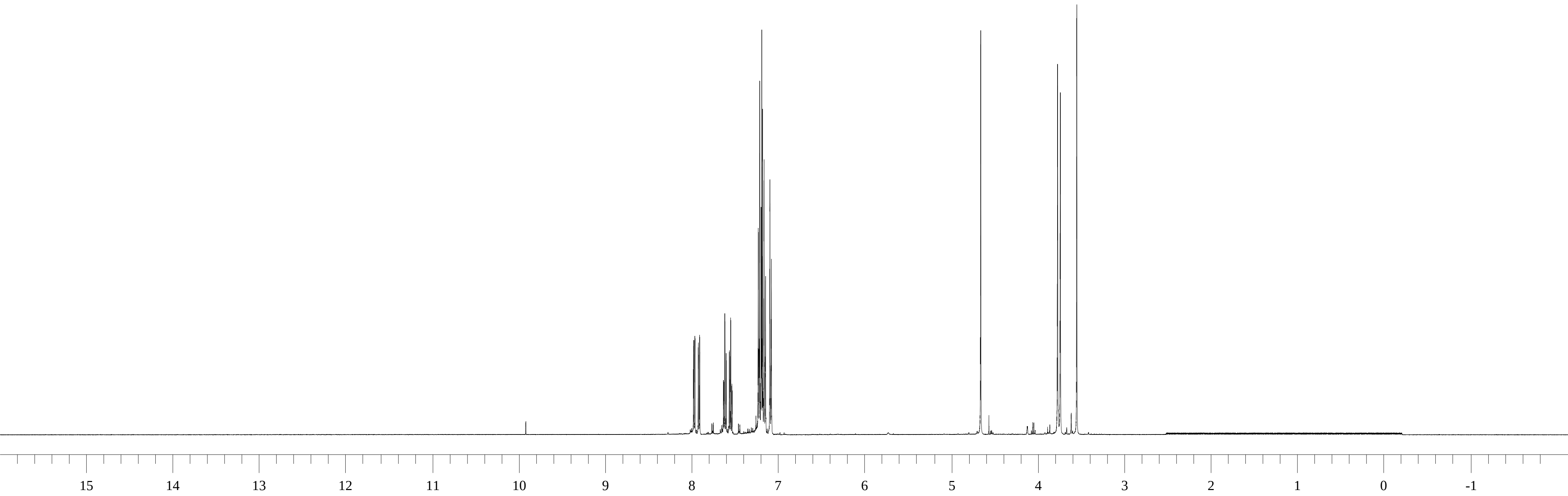

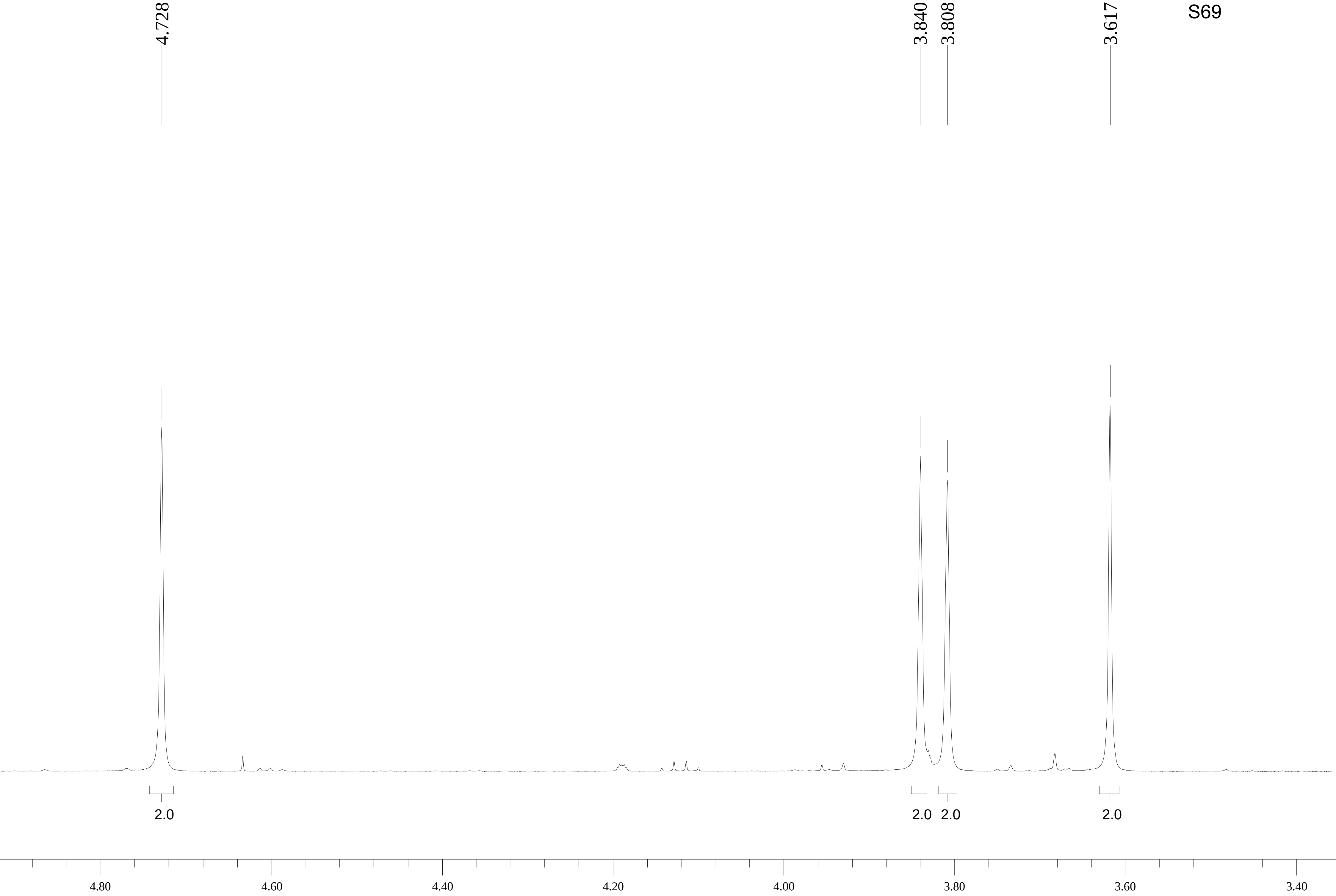
4.728
3.840
3.808
3.617
2.0
2.0
2.0
2.0
4.80
4.60
4.40
4.20
4.00
3.80
3.60
3.40

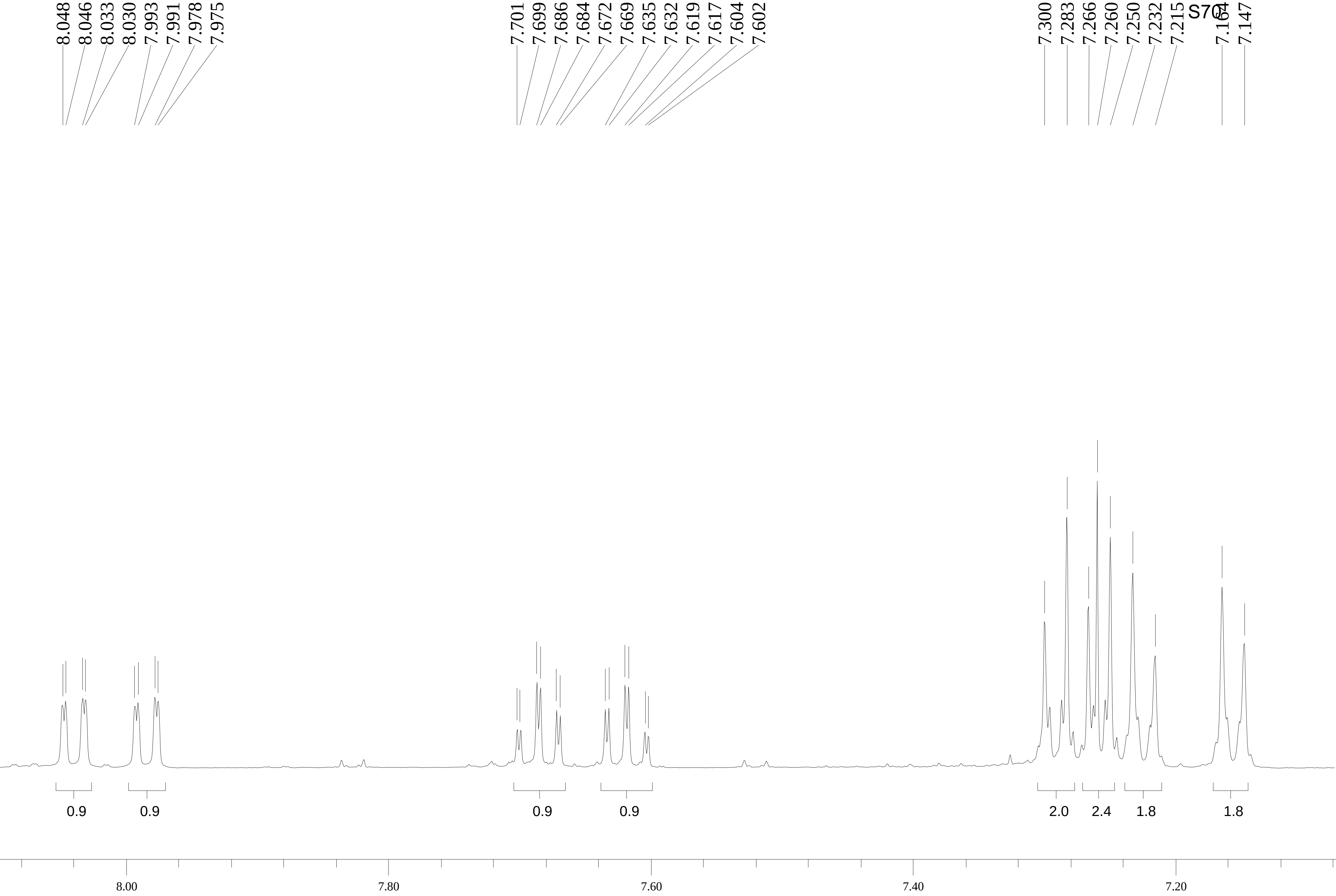
8.048
8.046
8.033
8.030
7.993
7.991
7.978
7.975
7.701
7.699
7.686
7.684
7.672
7.669
7.635
7.632
7.619
7.617
7.604
7.602
7.300
7.283
7.266
7.260
7.250
7.232
7.215
7.147
0.9
0.9
0.9
0.9
2.0
2.4
1.8
1.8
8.00
7.80
7.60
7.40
7.20

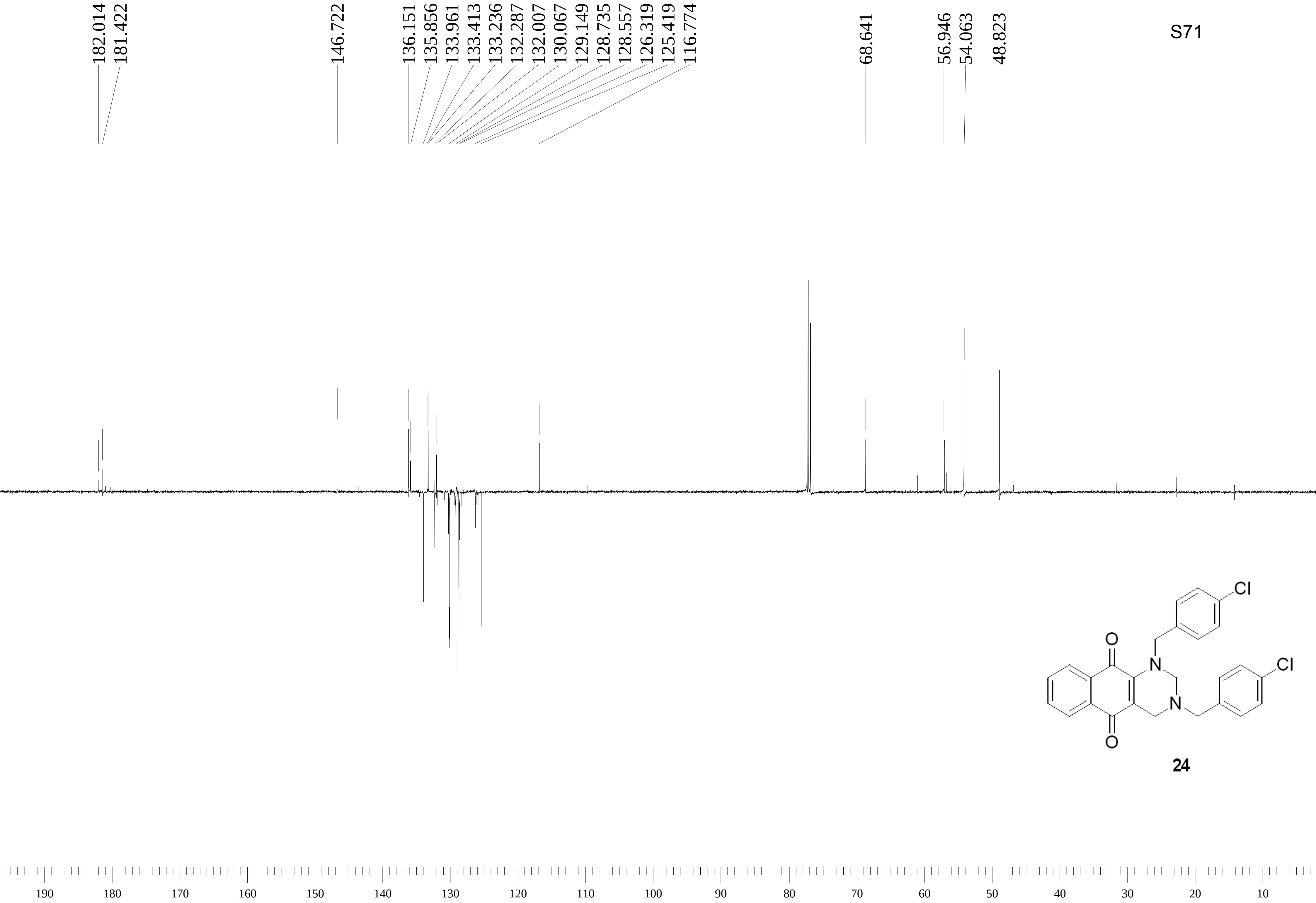
182.014
181.422
146.722
136.151
135.856
133.961
133.413
133.236
132.287
132.007
130.067
129.149
128.735
128.557
126.319
125.419
116.774
68.641
56.946
54.063
48.823
O
N
N
Cl
Cl
O
190 180 170 160 150 140 130 120 110 100 90 80 70 60 50 40 30 20 10

24

HELVECIO

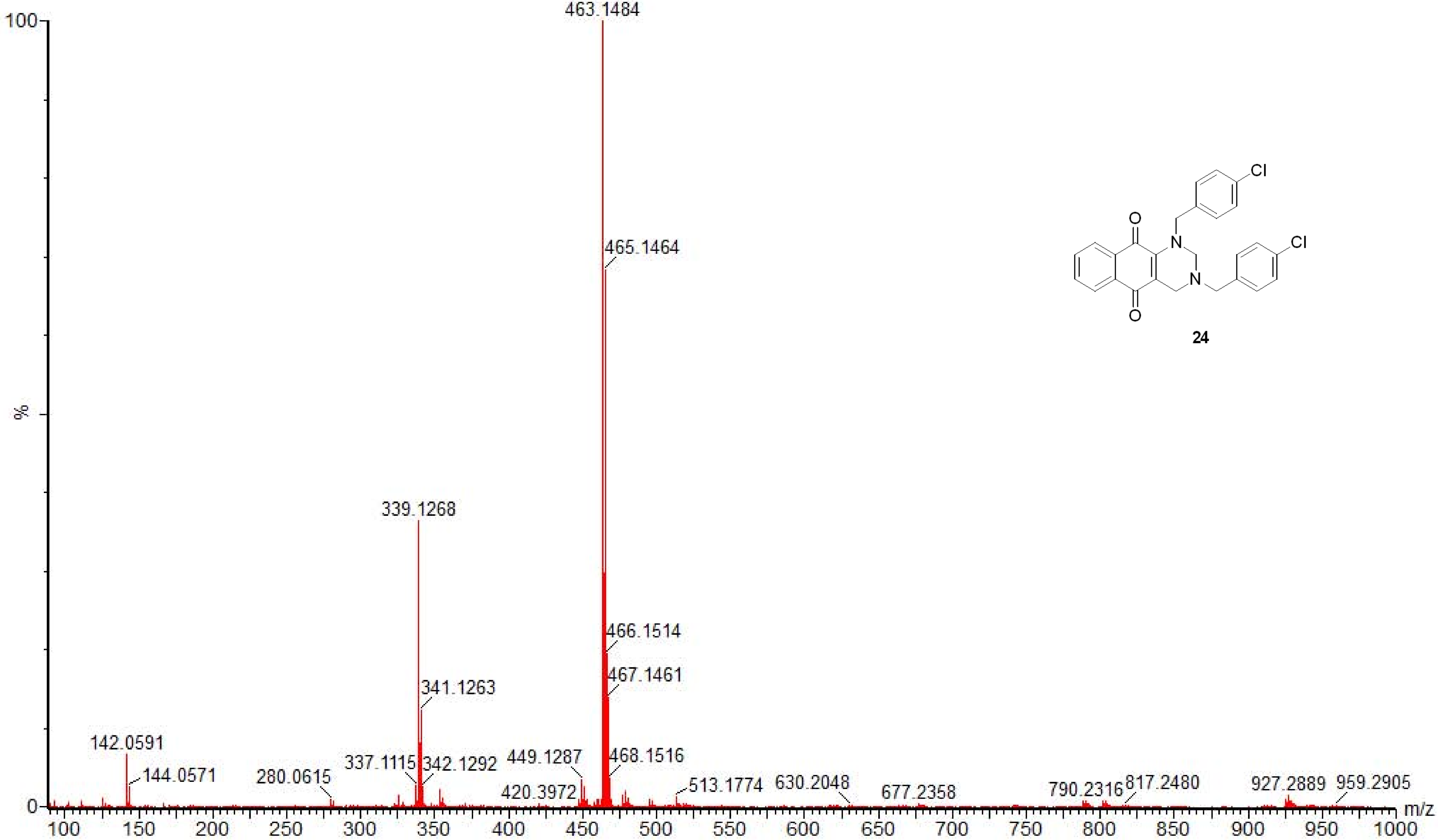
463.1484
465.1464
339.1268
466.1514
467.1461
341.1263
142.0591
144.0571
280.0615
337.1115
342.1292
449.1287
468.1516
420.3972
513.1774
630.2048
677.2358
790.2316
817.2480
927.2889
959.2905
100
%
0
m/z
100 150 200 250 300 350 400 450 500 550 600 650 700 750 800 850 900 950 1000
O
O
N
N
Cl
Cl

24

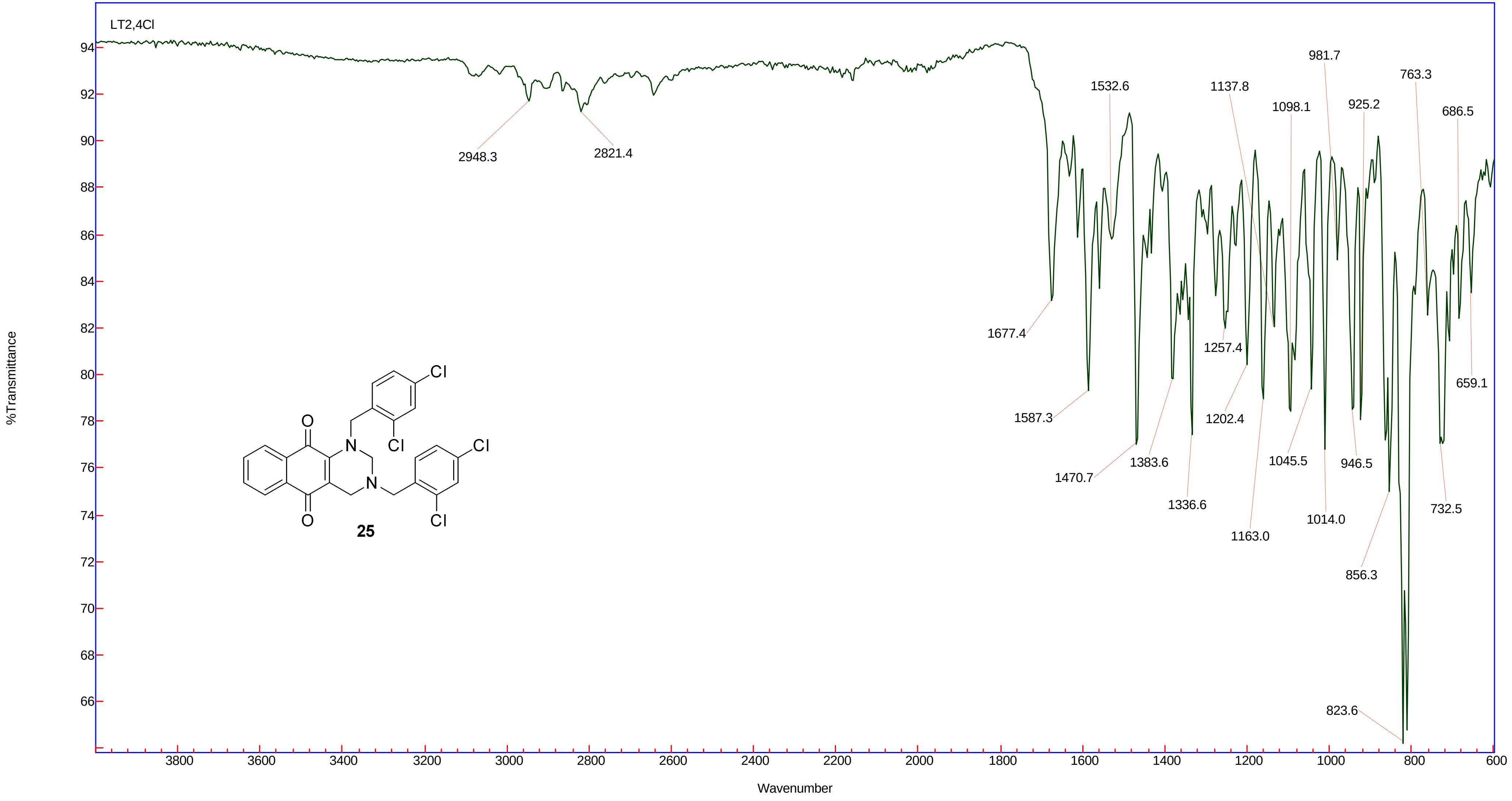
LT2,4Cl
%Transmittance
Wavenumber
2948.3
2821.4
1677.4
1587.3
1532.6
1470.7
1383.6
1336.6
1257.4
1202.4
1163.0
1137.8
1098.1
1045.5
1014.0
981.7
946.5
925.2
856.3
823.6
763.3
732.5
686.5
659.1
Cl
O
N
25

Name
LT2,4Cl

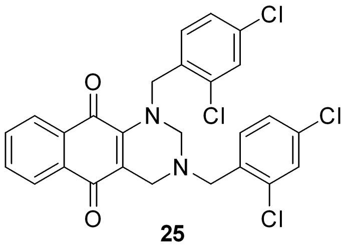

25

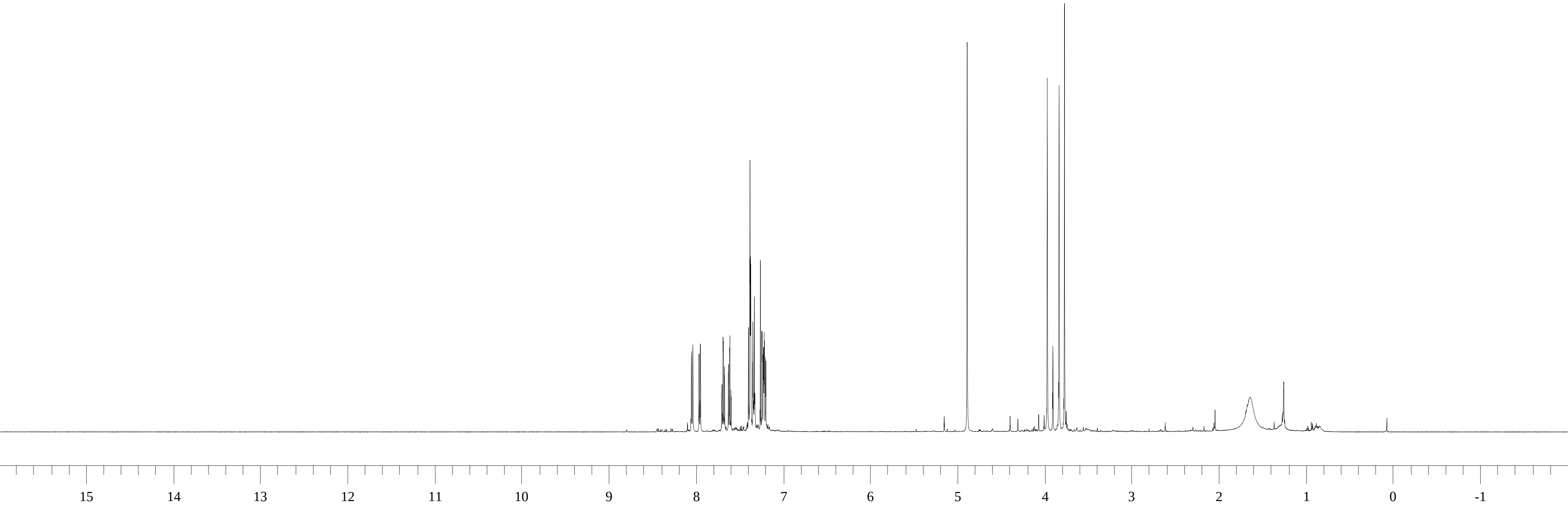

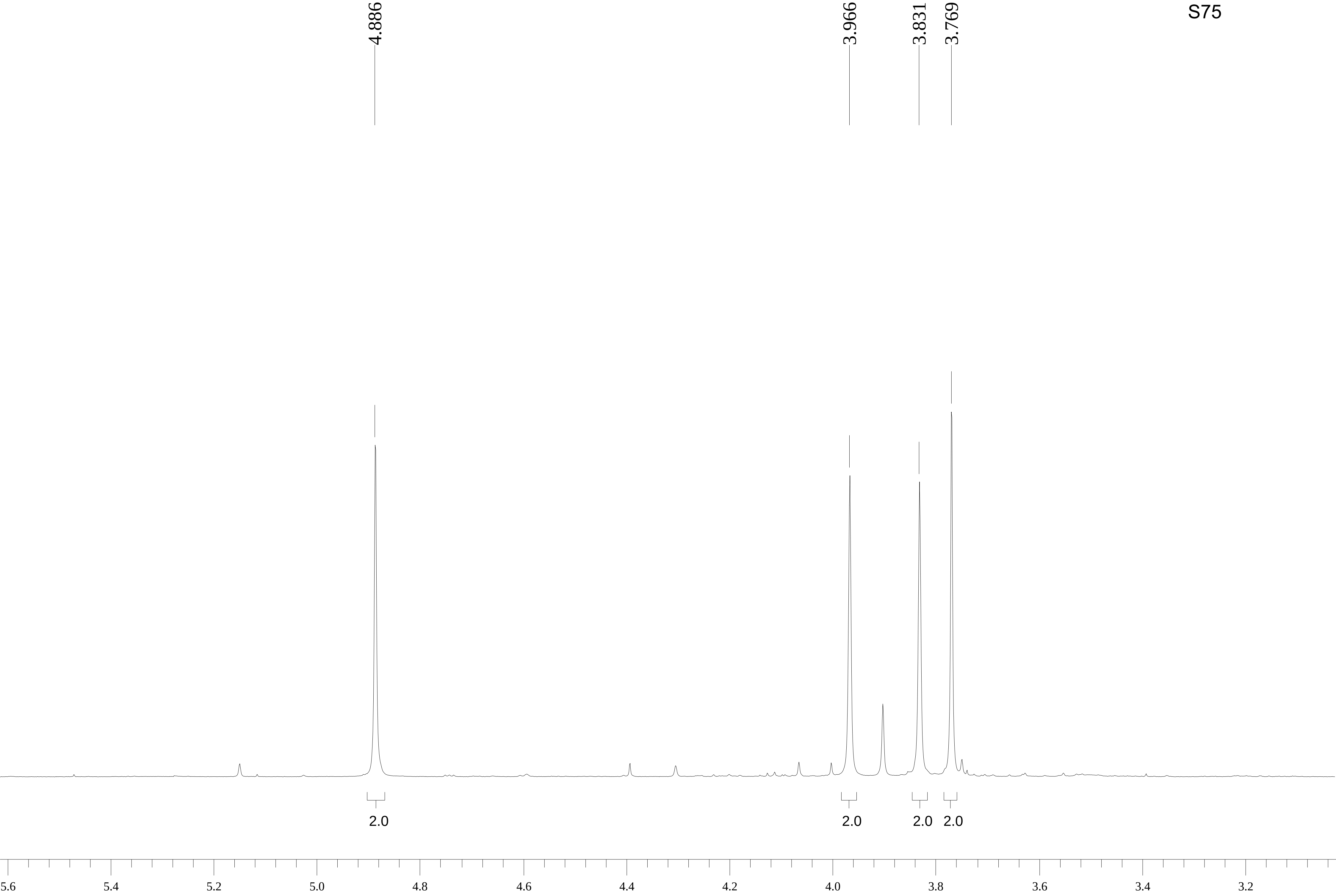
4.886
3.966
3.831
3.769
2.0
2.0
2.0
2.0
5.6
5.4
5.2
5.0
4.8
4.6
4.4
4.2
4.0
3.8
3.6
3.4
3.2

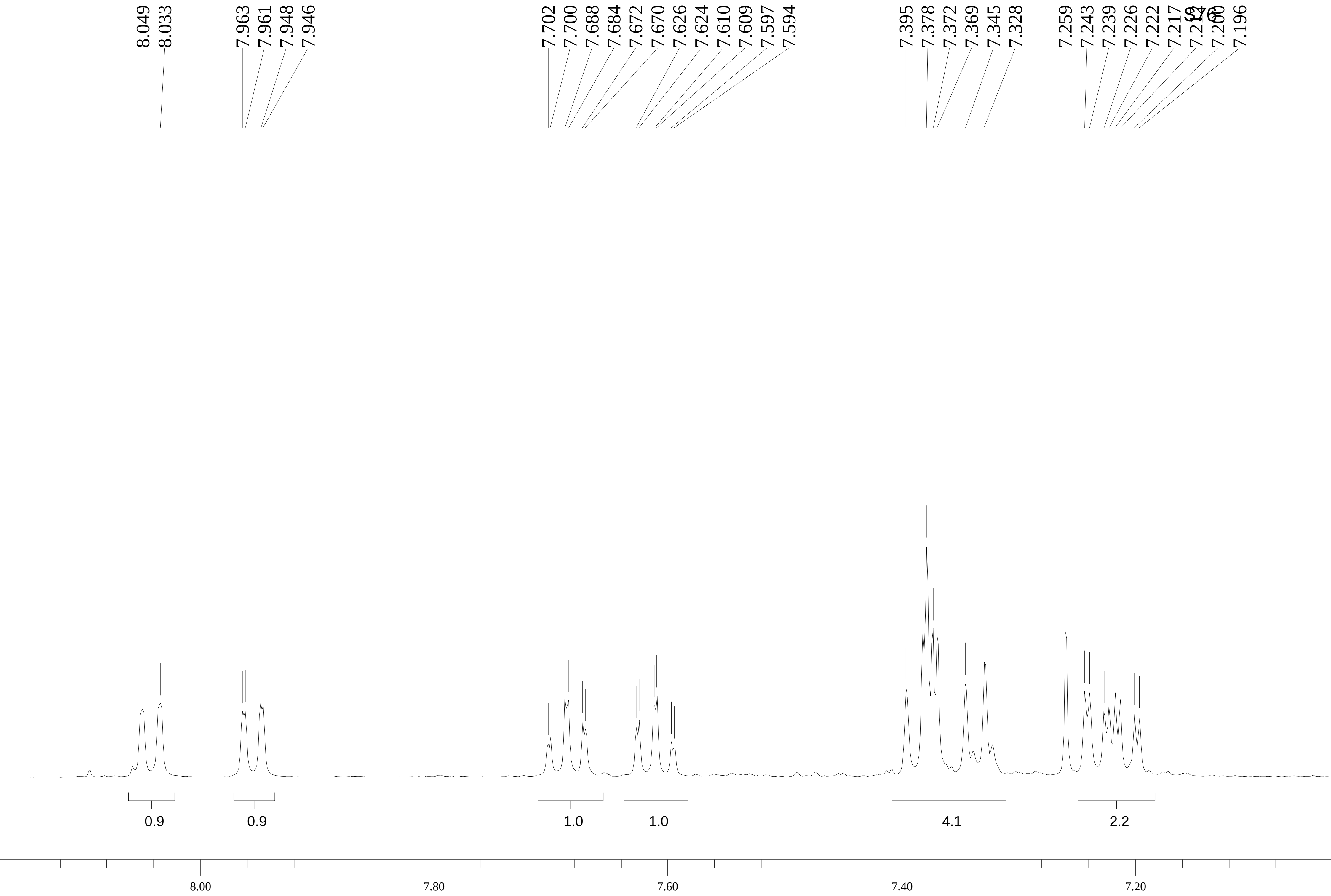

8.049
8.033
7.963
7.961
7.948
7.946
7.702
7.700
7.688
7.684
7.672
7.670
7.626
7.624
7.610
7.609
7.597
7.594
7.395
7.378
7.372
7.369
7.345
7.328
7.259
7.243
7.239
7.226
7.222
7.217
7.196
0.9
0.9
1.0
1.0
4.1
2.2
8.00
7.80
7.60
7.40
7.20

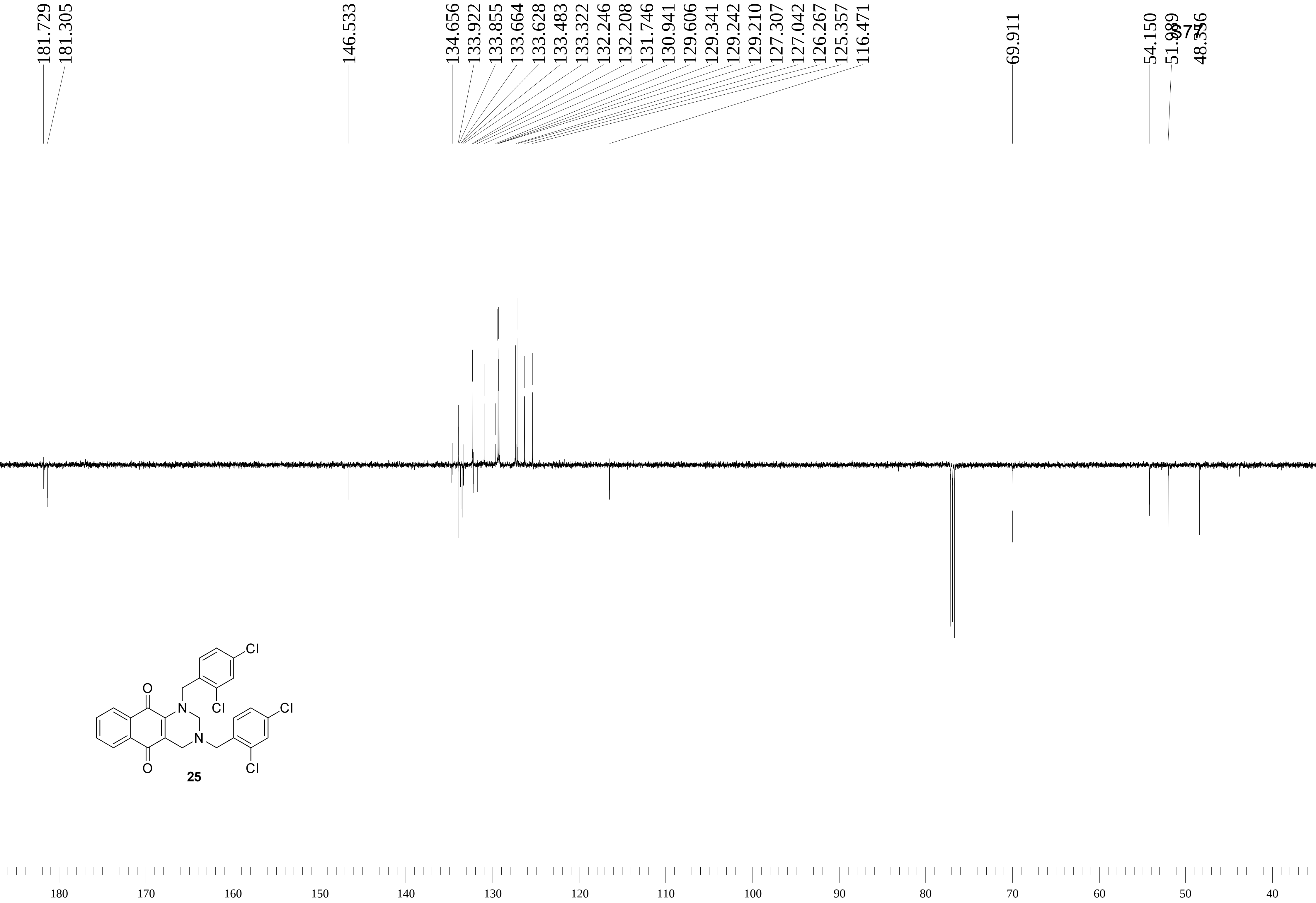

181.729
181.305
146.533
134.656
133.922
133.855
133.664
133.628
133.483
133.322
132.246
132.208
131.746
130.941
129.606
129.341
129.242
129.210
127.307
127.042
126.267
125.357
116.471
69.911
54.150
Cl
O
N
Cl
Cl
N
O
Cl
25
180
170
160
150
140
130
120
110
100
90
80
70
60
50
40

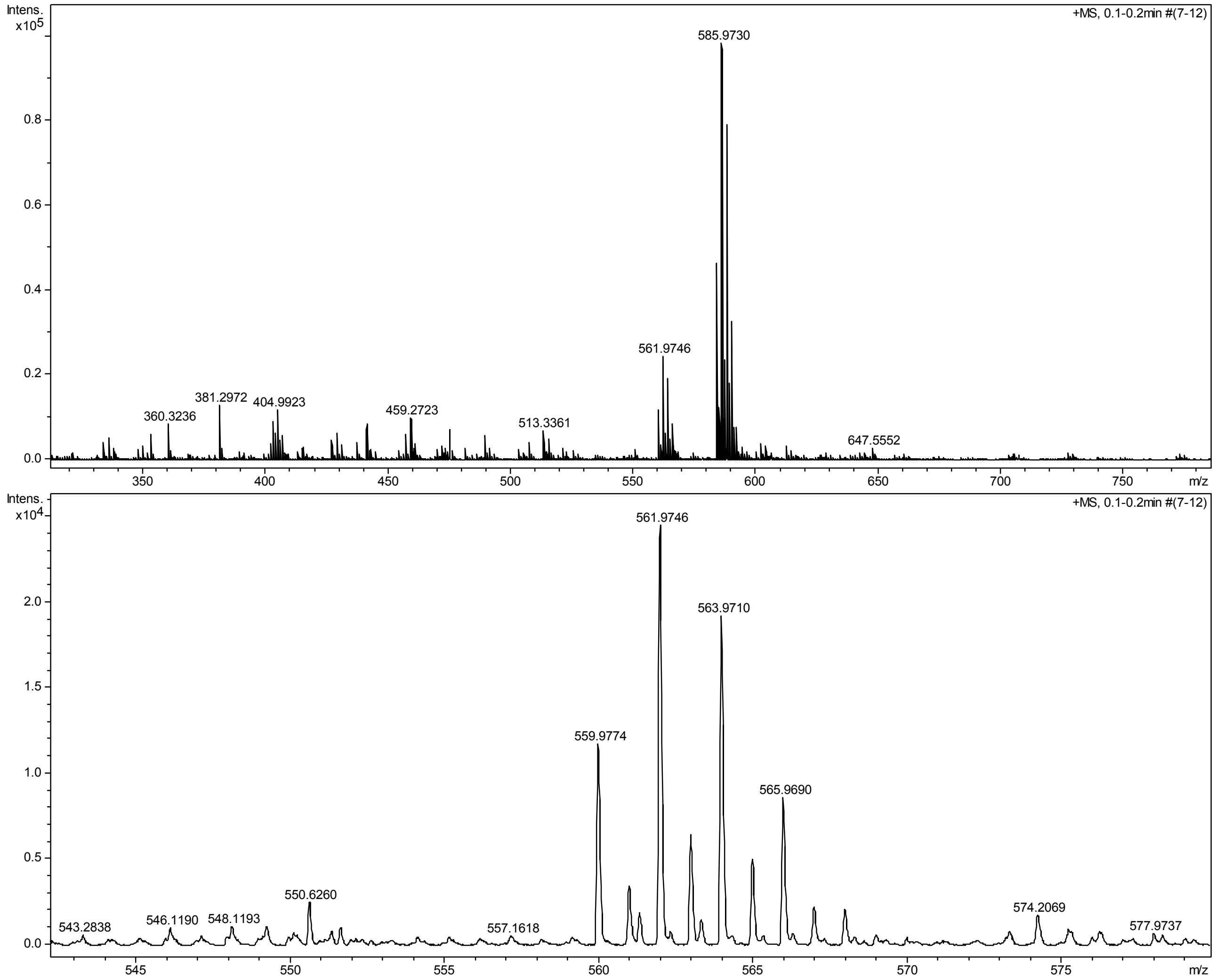

Intens.
x10^5
+MS, 0.1-0.2min #(7-12)
585.9730
561.9746
381.2972
404.9923
360.3236
459.2723
513.3361
647.5552
0.8
0.6
0.4
0.2
0.0
350
400
450
500
550
600
650
700
750
m/z
Intens.
x10^4
+MS, 0.1-0.2min #(7-12)
561.9746
563.9710
559.9774
565.9690
550.6260
574.2069
543.2838
546.1190
548.1193
557.1618
577.9737
2.0
1.5
1.0
0.5
0.0
545
550
555
560
565
570
575
m/z

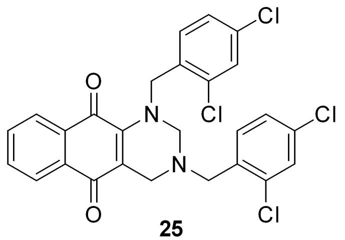

O
N
N
O
Cl
Cl
Cl
Cl

25
